# Data on the docking of millet-derived secondary metabolites as multi-target ligands for diabetes

**DOI:** 10.1016/j.dib.2025.111290

**Published:** 2025-01-11

**Authors:** Komal G. Lakhani, Rasmieh Hamid, Poojaben Prajapati, Kirankumar P. Suthar, Sheetal Gupta, Visha Rathod, Saumya Patel

**Affiliations:** a Department of Plant Molecular Biology and Biotechnology, N. M. College of Agriculture, Navsari Agricultural University, Navsari, Gujarat, India; b Department of Plant Breeding, Cotton Research Institute of Iran (CRII), Agricultural Research, Education and Extension Organization (AREEO), Gorgan, Iran; c Department of Botany, Bioinformatics, and Climate Change Impacts Management, School of Sciences, Gujarat University, Ahmedabad 380009, India; d ICAR-Indian Agricultural Research Institute, New Delhi 110012, India; e National Forensic Science university, Gandhinagar, India

**Keywords:** Metabolic diseases, Diabetes, Obesity, Molecular docking, Plant bioactive compounds

## Abstract

The deterioration of human health due to unhealthy lifestyle and dietary habits has led to a worldwide increase in various metabolic diseases that significantly affect public health. Diabetes is one of the most serious health problems, is caused by abnormal metabolic processes and is becoming increasingly common. According to World Health Organisation (WHO) reports, a significant proportion of the world's population suffers from these diseases and their incidence continues to rise at an alarming rate. These metabolic disorders are characterised by elevated blood sugar levels, which serve as a warning sign for a variety of other health problems. Factors contributing to these diseases include a high-fat diet, insufficient physical activity, genetic predisposition, lack of exercise and underlying diseases. Diabetes mellitus, a fast-growing chronic metabolic disease, is characterised by insufficient insulin production by the pancreas or the body's inability to use insulin action. Various strategies are recommended by health and nutrition experts to manage this condition, including lifestyle changes, exercise, low-carbohydrate and low-fat diets and intermittent fasting. In cases where these measures prove insufficient, medication may be prescribed. However, the development of multi-drug therapies for metabolic disorders has proven to be an attractive field for pharmacists as they address several diseases simultaneously. Despite the promising effects of multi-drug therapies, the high costs and potential side effects associated with recently developed drugs necessitate alternative approaches. The utilisation of natural bioactive compounds from plant extracts represents a promising high-throughput strategy. This approach utilises network pharmacology and screening methods to identify potential ligands that act as inhibitors for the treatment of complex, interconnected diseases. In the current investigation, we used a molecular docking approach to investigate secondary metabolites from millet as potential multi-target ligands for the treatment of diabetes and obesity.

Specifications Table

**Table:** 

Subject	Computational biology
Specific subject area	Interdisciplinary field includes organic chemistry, biochemistry, and biology. Drug design and discovery from plant sources.
Type of data	Raw and Analysed data including Figures and Tables
Data collection	Bioactive compounds present in seed extract of millet were analysed using GC–MS and used as a Ligand Ligand based molecular docking bind against target protein using YASARA software version and visualized by Biovia Discovery Studio Visualizer 2020
Data source location	Institute: Department of Botany, Bioinformatics, and Climate Change Impacts Management, School of Sciences, Gujarat University, Ahmedabad, City/Town/Region: Ahemdabad, Gujarat Country: India
Data accessibility	Repository name: Dataset for millet derived compound fight against diabietic protein alpha-amylase and human maltase gluco‑amylase Data identification number: 10.17632/5nnsfvzz3m.1 Direct URL to data https://data.mendeley.com/datasets/5nnsfvzz3m/1
Related research article	Lakhani, K. G., Hamid R, Prajapati P, Patel S, Dwivedi A. and Suthar KP (2024) From Millets to Medicine: ADMET Insights into Diabetes Management with *P. sumatrense* Compounds. Biocatalysis and Agricultural Biotechnology, 61: 103,396. 10.1016/j.bcab.2024.103396. [1]

## 1 Value of the Data

• This dataset provides insight of metabolites present in grains millet identified by Gas Chromatography–Mass Spectroscopy (GC–MS) act as potent anti-diabetic agent by inhibiting two important enzymes such as alpha-amylase (2QMK) and maltase gluco‑amylase (2QMJ).
• This screening process provides valuable insights into the active natural compounds contained in millet extracts that can influence certain biochemical metabolic pathways and thus contribute to the control and inhibition of metabolic disorders such as diabetes.
• In addition, computational biologists and pharmaceutical scientists can use this information as a basis for virtual analyses aimed at developing new synthetic analogues with reduced side effects and improved bioactivity against these targets.
• This *In silico* studies serve as an invaluable resource, empowering experimental researchers by seamlessly bridging computational insights with experimental validation.
• By expediting the identification of promising compounds and elucidating their interactions with anti-diabietic targets, these studies substantially accelerate the research timeline, thereby revolutionizing and optimizing the drug discovery pipeline.
• Although the exact mechanisms of action and identification of key ingredients in herbal remedies and dietary supplements that facilitate the control of glucose metabolism are still largely unexplored, the dataset enables the identification of novel drugs for diabetes.
• The binding interactions of little millet derived compounds with two different diabetes-related target proteins facilitate the effective utilisation of this data. This in turn provides important information to drive organic synthesis and the search for analogue pairs in the context of virtual drug design.

## 2 Background

Diabetes is a chronic, hyperglycaemic disease that poses a major public health challenge and is characterised by persistently elevated blood glucose levels [2]. The pancreatic islet cells, particularly the beta cells within them, are often damaged in diabetes, leading to resistance or impaired insulin secretion, which in turn triggers a hyperglycaemic state [3]. In underdeveloped countries with inadequate access to treatment, diabetes is increasing at an alarming rate. The International Diabetes Federation (IDF) predicts that the number of diabetes cases will rise to 693 million by 2045, making it a potentially fatal chronic disease. There are many medications available to control blood sugar levels that do not completely cure diabetes, but focus on controlling or adjusting insulin secretion. Long-term use of therapeutic diabetes medications is often associated with various adverse effects and suboptimal patient compliance [4].

Globally, the increase in diabetes cases can be attributed to changes in dietary habits, particularly the increased consumption of street or packaged foods that are high in saturated fats and sugars but low in essential nutrients [5]. Effective diabetes management relies heavily on lifestyle therapy, which emphasises regular exercise, weight control and the consumption of nutrient-rich foods that support the gradual release of sugars [6]. Meals that are high in fibre and unsaturated fatty acids and have a low glycaemic index are particularly beneficial for controlling diabetes as they help to reduce the rate of sugar absorption into the bloodstream.

Millet, an ancient group of grains, has established itself as a nutrient-rich alternative to staple foods such as rice and wheat. With their nutrient-rich profile, including higher levels of protein, fibre and essential minerals, millets outperform many conventional grains when it comes to meeting nutritional needs and treating various lifestyle-related diseases [7]. Little millets, a group of minor millets often referred to as “nutra-cereals',” are widely consumed by the population in the tribal areas of developing countries in Africa and Asia. The declaration of 2023 as the International Year of Millets by the United Nations has drawn attention to the potential of underutilised crops like little millet to address global challenges [8,9]. This grain is a rich source of various phytochemical compounds, including polyunsaturated fatty acids, flavonoids, organic acids and phenolic compounds [10]. Regular consumption of little millets is associated with the treatment of various diseases such as obesity, diabetes, hypertension and cardiovascular diseases [1]. Little Millet, have unique interactions with alpha-amylase and maltase-glucoamylase due to their composition. Their role in modulating these enzymes can influence carbohydrate digestion and blood sugar control, making them beneficial for managing diabetes. This data description presents the bioactive compounds identified in the grains of little millets that act as ligands to inhibit the activity of the target proteins α-amylase and human maltase-glucoamylase and thus contribute to the treatment of metabolic disorders such as diabetes.

## 3 Data Description

Alpha-amylases and maltase gluco‑amylase are important digestive enzymes that control glucose levels in the treatment of diabetes by lowering postprandial hyperglycaemia (PPHG) levels in animals and humans. To maintain metabolic functions and digestion in the body, potent inhibitors of alpha-amylase and maltase‑gluco-amylase need to be identified to prevent an increase in blood sugar levels due to slow release of glucose due to slow digestion [11]. Pancreatic α-amylase and maltase glucoamylase inhibitors, often referred to as potent starch or carbohydrate blockers, are considered an effective intervention to mitigate hyperglycaemia by curbing the influx of glucose into the bloodstream by attenuating the hydrolysis and absorption of starch from food [12]. In carbohydrate metabolism, these two enzymes are involved in a stepwise process in which maltose is formed from polysaccharide molecules by hydrolysis, which is facilitated by alpha-amylase [13] Through the activity of maltase, glucoamylase is an exo-type enzyme that hydrolyses polymeric carbohydrates by facilitating their subsequent absorption through hydrolysis of complex carbohydrates. The pharmaceutical industry offers a plethora of antidiabetic drugs. However, excessive inhibition of blood glucose-lowering enzymes by these agents can lead to gastrointestinal complications, including diarrhoea [14]. There are many home remedies to control blood glucose levels, which are very popular among patients. However, many patients avoid their consumption due to their taste and unpleasant odour.

Millets are nutrient-rich grains known for their richness in bioactive compounds such as alkaloids, phenols and flavonoids. These metabolites show remarkable efficacy in regulating blood sugar levels and reducing the risks associated with prediabetes. Despite their nutritional and therapeutic potential, the use of small millets, particularly small millets, is still limited and often considered a pseudo-cereal. The discovery of the anti-diabetic properties of small millets increases their value, especially in the development of functional millet-based foods. As these grains can be easily integrated into the daily diet and do not require special preparation, they are a good choice for health-conscious consumers. This study aims to investigate the inhibitory effect of secondary metabolites in millet grains on alpha-amylase and human metabolism to scientifically substantiate their role in combating diabetes and to support millet producers.

Tables 1 and 2 present the binding free energy data obtained from docking experiments investigating the interactions of bioactive compounds from millet with the proteins α-amylase and human maltase-glucoamylase, respectively. These proteins are important in the context of diabetes-related metabolic disorders. The interactions between the ligands and the active sites of the target proteins are elucidated by the amino acid residues listed in Tables 3 and 4 (see suppl. Table 1 and suppl. Table 2). The result of molecular docking of top five compounds for both proteins by involvement of amino acids illustrated in Fig. 1, Fig. 2, Fig. 3, Fig. 4, Fig. 5, Fig. 6, Fig. 7, Fig. 8, Fig. 9, Fig. 10, Fig. 11, Fig. 12, Fig. 13, Fig. 14, Fig. 15, Fig. 16, Fig. 17, Fig. 18, Fig. 19, Fig. 20, Fig. 21, Fig. 22, Fig. 23, Fig. 24, Fig. 25, Fig. 26, Fig. 27, Fig. 28, Fig. 29, Fig. 30 for 2QMK and Fig. 31, Fig. 32, Fig. 33, Fig. 34, Fig. 35, Fig. 36, Fig. 37, Fig. 38, Fig. 39, Fig. 40, Fig. 41, Fig. 42, Fig. 43, Fig. 44, Fig. 45, Fig. 46, Fig. 47, Fig. 48, Fig. 49, Fig. 50, Fig. 51, Fig. 52, Fig. 53, Fig. 54, Fig. 55, Fig. 56, Fig. 57, Fig. 58, Fig. 59, Fig. 60 for 2QMJ protein.

**Table 1:** The calculated binding energy of co-crystal ligand and natural compounds derived from Little Millet with Alpha-amylase (PDB ID: 2QMK) using YASARA structure.

Ligand	Binding energy [kcal/mol]	Dissociation constant [pM]	Pubchem Id	Efficiency [kcal/(mol*Atom)]	Con. Surf [A^2]
Actinobolin	8.0090	1,346,868.3750	54,688,606	3814	228.91
Xanthosine	7.6420	2,502,302.0000	64,959	3821	217.72
Arbutin	7.2870	4,555,732.0000	440,936	3835	242.47
Sinapic acid	7.2440	4,898,663.5000	637,775	4527	224.22
Maltose	7.0630	6,648,939.0000	6255	3071	245.87
L-Tryptophan	6.8230	9,969,530.0000	6305	4549	229.05
Caffeic acid	6.8020	10,329,229.0000	689,043	5232	174.32
Pyrido[3,4-d] imidazole	6.7150	11,962,990.0000	541,512	4477	177.65
Prosta 5,13‑dien-1-oic acid	6.3420	22,451,862.0000	21,145,257	2883	344.20
L-Tyrosine	6.3090	23,737,866.0000	6057	4853	211.59
4-Fluoro-3-[1‑hydroxy-2-(methylamino)ethyl] phenol	6.1690	30,065,128.0000	183,823	4745	192.98
D-Fructopyranose	6.1210	32,602,246.0000	2,723,872	5101	141.20
3-Ethoxy-4-hydroxymandelic acid	6.1150	32,934,082.0000	193,792	4077	212.79
Gluconic acid	6.0640	35,894,596.0000	10,690	4665	157.06
2, 5-Dimethoxy-4-ethylamphetamine	6.0430	37,189,664.0000	27,402	3777	251.56
Gulonic acid	6.0360	37,631,656.0000	152,304	4311	183.82
1,2 O-Isopropylidene-alpha-d- glucofuranose	6.0320	37,886,576.0000	87,704	4021	198.08
Phenylphrine	6.0240	38,401,612.0000	6041	5020	185.62
p-hydroxynorephedrine	5.9890	40,738,476.0000	11,099	4991	176.04
Synephrine	5.9120	46,392,368.0000	7172	4927	185.58
Mannoic acid	5.8590	50,733,644.0000	3,246,006	4507	162.35
5-(2-Aminopropyl)−2-methylphenol	5.8570	50,905,192.0000	167,995	4881	217.63
I-Guanidinosuccinimide	5.8130	54,829,528.0000	541,546	5813	147.59
Cathine	5.7270	63,394,756.0000	441,457	5206	142.79
L-histidine	5.6290	74,797,712.0000	6274	5117	196.36
2-Propenoic acid, n-pentadecyl ester	5.4370	211,554,512.0000	543,579	3198	263.54
Heptanedioic acid	5.4230	105,898,128.0000	385	4930	177.57
2-Butenedioic acid	5.3410	12,167,224.0000	44,972	6676	114.06
10-Octadecenoic acid	5.3140	127,287,672.0000	445,639	2657	170.37
L- Alanine ethylamide, (S)	4.5230	483,723,328.0000	7,128,993	5654	151.43
11-Eicosenoic acid	4.1950	841,442,112.0000	5,282,768	1907	284.23

**Table 2:** The calculated binding energy of co-crystal ligand and natural compounds derived from Little Millet with Human Maltase Gluco-amylase (PDB ID: 2QMJ) using YASARA structure.

Human Maltase Glucoamylase	Binding energy [kcal/mol]	Dissociation constant [pM]	Pubchem id	Efficiency [kcal/(mol*Atom)]	Con. Surf [A^2]
Actinobolin	6.7980	10,399,200.0000	54,688,606	3237	238.31
Xanthosine	6.7940	10,469,645.0000	64,959	3397	192.37
Arbutin	6.8410	9,671,203.0000	440,936	3601	216.81
Sinapic acid	6.8150	10,105,057.0000	637,775	4259	255.18
Caffeic acid	6.6320	13,761,936.0000	689,043	5102	203.76
Tyrosine	6.4910	17,459,584.0000	6057	4993	202.60
Maltose	6.4390	19,061,208.0000	6255	2800	242.82
4-Fluoro-3-[1‑hydroxy-2-(methylamino)ethyl] phenol	6.3740	1,271,398.0000	183,823	4903	221.19
p-hydroxynorephedrine	6.3140	23,538,384.0000	11,099	5262	198.00
Cathine	6.2080	28,149,822.0000	441,457	5644	210.68
L-Histidine	6.1330	31,948,568.0000	6274	5575	202.80
Gluconic acid	6.1320	32,002,536.0000	10,690	4717	153.44
L-Tryptophan	6.0990	33,835,588.0000	6305	4066	224.03
L-phenylephrine	6.0760	35,174,908.0000	6041	5063	194.01
Synephrine	6.0570	36,321,196.0000	7172	5048	188.81
Pyrido[3,4-d] imidazole, 1,6-dicarboxylic acid	5.9910	40,601,192.0000	541,512	3994	177.39
2,5-Dimethoxy-4-ethylamphetamine	5.8900	48,147,392.0000	27,402	3681	242.07
1,2 O-Isopropylidene-alpha-d- glucofuranose	5.7480	61,187,136.0000	87,704	3832	173.91
Prosta 5,13‑dien-1-oic acid	5.6080	341,086,112.0000	21,145,257	2150	266.44
3-Ethoxy-4-hydroxymandelic acid	5.4560	100,161,072.0000	193,792	3637	202.09
I-Guanidinosuccinimide	5.4440	102,210,400.0000	541,546	5444	170.97
D-Fructopyranose	5.3570	118,376,896.0000	2,723,872	4464	151.02
Gulonic acid	5.3120	127,718,080.0000	152,304	4086	163.09
5-(2-Aminopropyl)−2-methylphenol	5.1960	155,339,360.0000	167,995	4330	203.73
11-Eicosenoic acid	5.0260	206,963,216.0000	5,282,768	2285	277.81
Mannoic acid	4.9290	243,778,336.0000	3,246,006	3792	158.91
2-Butenedioic acid	4.9140	250,028,912.0000	444,972	6143	130.29
Heptanedioic acid	4.7170	348,652,800.0000	385	4288	186.80
L- Alanine ethylamide (S)	4.3040	700,045,312.0000	7,128,993	5380	173.21
10-Octadecenoic acid	4.2210	805,315,328.0000	445,639	2111	269.31
2-Propenoic acid, n-pentadecyl ester	3.5670	2,428,590,848.0000	543,579	1783	241.97

**Table 3:** Interacting amino acids of the Alpha-amylase (2QMK) with the ligands derived from Little Millet.

Ligands	Hydrogen Bonds	Alkyl and Pi bonds	Attractive Charges	Unfavourable bonds
Actinobolin	**Conventional HB:** 6; Arg252(2.47), Gln8(1.18), Asp402(2.09), Arg421(2.73), Gly403(2.46), Pro332(2.45)	**Alkyl:** 1; Arg398(3.94)		**Unfavourable Donor-Donor:** 1; Gln8(1.18)
Xanthosine	**Conventional HB:** 2; Arg10(2.43), Gln7(2.13) **Carbon HB:** 6; Pro4(2.56), Pro4(2.97), Gln8(2.71). Thr11(2.87), Gly334(2.33), Pro332(3.08)	**Pi-alkyl:** 2; Pro4(4.84), Pro4(4.86) **Amide Pi stacked:** 1; Arg10(4.83)		**Unfavourable Donor-Donor:** 1; Arg398(2.37)
Arbutin	**Conventional HB:** 3; Gly403(2.23); Gln7(2.83); Gln8(2.41) **Carbon Hydrogen Bond:** 2; Asp402(2.13); Gly334(2.12)	**Pi-Alkyl**: 1; Pro4(4.09)		
Sinapic acid	**Conventional HB:** 5; Thr6(2.53), Arg10(2.82), Arg252(2.68), Arg398(3.06), Ser289(3.07) **Carbon Hydrogen Bond:** 2; Gln8(2.60), Ser289(2.90)			**Unfavourable Negative-Negative:**1; Asp402(5.26)
Maltose	**Conventional HB:** 6; Thr6(2.71), Thr6(2.50), Gln7(2.24), Ser289(2.43), Arg10(2.96), Arg398(2.54) **Carbon HB:** 4; Gln8(2.75), Gly334(2.36), Pro4(2.60), Pro332(2.46)			**Unfavourable Acceptor-Acceptor:** 1; Pro332(2.93)
L-Tryptophan	**Conventional HB:** 2; Arg195(2.54), Asn298(2.50) **Carbon Hydrogen Bond:** 2; Glu233(2.78), Asp300(2.61)	**Pi-Pi stacked:** 2; Tyr62(5.00), Tyr62(3.89)	**Attractive Charge:** 2; Arg195(4.80), Glu233(4.27) **Salt-Bridge Attractive Charge:** 2; Asp300(2.90), Asp300(2.16)	
Caffeic acid	**Conventional HB:**2; Arg252(2.76), Arg421(2.35) **Carbon Hydrogen Bond:** 2; Pro332(2.70), Asp402(2.26)			
Pyrido[3,4-d]imidazole	**Conventional HB:**2; Arg252(2.44), Gly334(2.57), **Carbon Hydrogen Bond:** 2; Pro332(3.03), Thr11(2.56)	**Pi-alkyl:** 1; Arg398(5.38) **Pi cation:** 1; Arg398(4.32) **Pi-Anion:** 1; Asp402(4.14)	**Attractive Charge:** 1; Asp252(4.57), Asp252(4.60), Arg398(5.29)	
Prosta 5,13‑dien-1-oic acid		**Alkyl:** 4 Ala198(4.35), Ile235(5.09), Leu162(4.02), Leu165(5.23), **Pi alkyl:** 2 Tyr62(5.42), Tyr62(4.00)		
L-Tyrosine	**Conventional HB:** 2 Arg195(2.64), Asn298(2.76)	**Pi-Pi stacked:** 1; Tyr62(4.43)	**Attractive charge:** 3 Arg195(4.57), Asp300(3.18), Glu233(4.48) **Salt-Bridge:**1 Asp300(2.56)	
4-Fluoro-3-[1‑hydroxy-2-(methylamino)ethyl] phenol	**Conventional HB:** 2; Gln8(2.36), Thr11(2.69) **Halogen:** 1; Gly334(3.56) **Carbon HB:** 4; Phe335(2.98), Phe335(2.55), Pro332(3.03), Gly334(2.90)	**Pi alkyl:** 1; Pro4(5.33) **Pi-Pi T shaped:** 1; Phe335(4.95)		**Unfavourable Donor-Donor:** 1; Arg398(1.11) **Unfavourable Acceptor-Acceptor:** 1; Gly334(2.91)
D-Fructopyranose	**Conventional HB:** 3; Arg398(2.49), Arg421(2.19), Pro332(2.45) **Carbon HB:** 2; Phe335(2.42), Thr11(2.85)			**Unfavourable Donor-Donor:** 1 Arg398(0.95)
3-Ethoxy-4-hydroxymandelic acid	**Conventional HB:** 2; Arg398(2.69), Gln8(1.84) **Carbon HB:** 2; Asp402(2.88), Thr11(2.60)	**Alkyl:** 1; Arg398(4.49),		**Unfavorable Negative-Negative:** 1 Asp402(3.58)
Gluconic acid	**Conventional HB:** 8; Arg398(1.91), Arg398(2.73), Arg421(2.86); Pro332(2.41); Gly334(3.03); Pro332(2.60); Asp402(2.52); Arg421(1.75)		**Attractive Charge:** 1; Arg252(4.54)	**Unfavourable donor-donor**: 1; Arg421(2.07),
2,5-Dimethoxy-4-ethylamphetamine	**Carbon HB:** 2; Arg10(3.02), Ser289(2.38)	**Alkyl:** 1; Pro4(4.32), **Pi-Alkyl:**1; Phe335(5.06)	**Salt-Bridge Attractive Charge:** 1; Asp402(2.04)	**Unfavourable Positive- Positive:** 1; Arg421(4.87)
Gulonic acid	**Conventional HB:** 1; Asp402(2.80) **Carbon HB:** 1; Pro332(2.31)	**Salt bridge:** 1; Arg252(2.78)		**Unfavourable donor-donor**: 2; Arg421(2.07), Arg421(2.07) **Unfavourable Acceptor – Acceptor:** 1; Gly334(2.73)
1,2 O-Isopropylidene-alpha-d- glucofuranose	**Conventional HB:** 4; Arg398(2.84), Pro332(2.63), Gly334(2.61), Gly403(2.85) Carbon HB: 2; Thr11(2.68), Ser289(2.54)	**Alkyl:** 2; Arg398(4.25), Pro332(5.24)		
Phenylphrine	**Conventional HB:** 3; Arg398(2.40), Asp402(2.44), Gly334(2.20) **Carbon HB:** 3; Arg10(2.59), Gln8(2.71), Thr6(2.96)	**Pi-Pi T shaped:** 1; Phe335(4.76)		
p-hydroxynorephedrine	**Conventional HB:** 4; Arg398(1.86), Pro332(2.76); Gly334(2.93); Gly334(2.42) **Carbon HB:** 1; Thr11(2.90)	**Alkyl:** 1; Arg398(4.53) **Pi-Alkyl:**1; Pro4(5.34) **Pi-Pi T shaped:** 1; Phe335(4.72)		
Synephrine	**Conventional HB:** 2; Asp402(2.79), Gln7(2.24) **Carbon HB:** 3; Arg10(2.34), Gln8(2.73), Thr6(3.04)	**Pi-Pi T shaped:** 1; Phe335(4.65)	**Attractive charge:** 1; Asp402(4.69)	
Mannoic acid	**Conventional HB:** 4; Arg398(2.32), Pro332(2.41); Gly334(2.97); Val401(2.16) **Carbon HB:** 2; Pro332(2.31), Phe335(2.92)		**Attractive charge:**2; Arg262(4.66), Arg398(5.10)	
5-(2-Aminopropyl)−2-methylphenol	**Carbon HB:**1; Glu233(2.94)	**Alkyl:** 1; Leu165(4.53) **Pi-Pi stacked:** 1; Tyr62(4.26)	**Salt Bridge Attractive Charge:** 2; Asp300(2.64), Asp300 (2.34) **Attractive Charge:** 1; Glu233(4.23)	
I-Guanidinosuccinimide	**Conventional HB:** 4; Arg303(2.58), Asn301(2.71), Ile312(2.74), Gly309(2.61)			
Cathine	**Conventional HB:** 2; Glu484(2.91), Gln390(2.39) **Carbon HB:** 1; Gln390(2.94)	**Alkyl:** 1; Arg389(4.54) **Pi-alkyl:** 1; Ala318(5.47) **Pi-Pi stacked:** 1; Trp388(3.80)		
Histidine	**Conventional HB:** 3; Asp300(2.44), Asn298(2.72), Arg195(2.57) **Carbon HB:** 1; Glu233(3.00)	**Pi-Pi stacked:** 1; Tyr62(4.17)	**Salt-Bridge Attractive charge:** 2; Asp300(2.80), Asp300(2.32), **Attractive charge:**2; Arg195(4.66), Glu233(2.53)	
2-Propenoic acid, n-pentadecyl ester	**Conventional HB:** 1; Gln390(2.33) **Carbon HB:** 2; Gln390(2.87), Glu484(2.59)	**Alkyl:** 5; Lys322(5.36), Val383(4.79), Arg343(4.78), Val383(4.49), Arg389(4.21) **Pi-Alkyl:** 7; Phe315(4.85), Trp316(4.30), Trp388(5.20), Trp388(5.38), Trp388(4.09), Trp388(5.04), Trp388(3.83)		
Heptanedioic acid	**Conventional HB:** 3; Gln7(2.33), Gln8(2.47), Asp402(2.92) **Carbon HB:** 1; Asp402(2.49)		**Salt Bridge:** 1; Arg398(4.76) **Attractive charge:** 1 Arg421(2.67)	
2-Butenedioic acid	**Conventional HB:** 2; Arg398(2.58), Arg421(2.51) **Carbon HB:** 1; Asp402(2.76) Pro332(2.67)		**Attractive charge:** 1; Arg398(4.97)	**Unfavourable Donor-Donor:** 1; Arg421(1.64)
10-Octadecenoic acid	**Conventional HB:** 1; Gln63(2.13)	**Alkyl:** 6; Ala198(4.52), Ala198(5.10), Leu162(4.70), Leu165(4.94), Lys200(4.22), Ile235(3.81) **Pi-alkyl:** 4; Tyr62(4.57), Tyr62(4.70), His201(4.98), His305(4.99) **Pi-Anion:** 1; Trp59(3.87)		10-Octadecenoic acid
L- Alanine ethylamide, (S)	**Conventional HB:** 6; Ala310(2.94), Asn301(3.07), Ile312(2.32), Gly309(2.12), Arg267(2.88), Arg346(2.68)	**Alkyl:** 1; Ala310(4.39) **Pi-Alky:** 1; Phe348(5.28)		L- Alanine ethylamide, (S)
11-Eicosenoic acid	**Conventional HB:** 2; Gly403(2.42), Arg421(2.87) **Carbon HB:** 1; Asp402(2.59)	**Alkyl:** 1; Pro4(4.96) **Pi-Alkyl:** 1; Phe335(5.38)	**Salt Bridge:** 1; Arg421(2.32)	11-Eicosenoic acid

**Table 4:** Interacting amino acids of the Human Maltase Gluco-amylase (2QMJ) with the ligands derived from Little Millet.

Ligands	Hydrogen Bonds	Alkyl and Pi bonds	Attractive Charges	Unfavourable bonds
Actinobolin	**Conventional HB:** 3; Arg471(1.98), Ser40(2.78), Thr196(2.98)	**Alkyl:** 1; Val244(4.11) **Pi-Alkyl:** 1; Trp194(4.98)		
Xanthosine	**Conventional HB:** 2; Asp759(1.97), Glu760(2.69) **Carbon HB:** 3; Ala764(2.56), Gly731(2.62), Glu760(2.52)	**Pi-alkyl:** 1; Lys765(4.78)		**Unfavourable Donor-Donor:** 1; Val699(1.96)
Arbutin	**Conventional HB:** 2; Arg647(2.78), Glu767(2.19) **Carbon HB:** 2; Asp649(2.56), Pro736(3.01)	**Pi-Pi T shaped:** 1; Tyr636(4.97) **Pi-Anion**:1; Glu767(4.00)		**Unfavourable Donor-Donor:** 1; Arg653(1.28)
Sinapic acid	**Conventional HB:** 5; Arg252(2.68), Arg10(2.82), Arg398(2.84), Ser289(3.07), Thr6(2.53)		**Attractive charge:** 1; Arg520(4.49)	**Unfavourable Negative- Negative:** 1; Asp402(5.26)
Caffeic acid	**Conventional HB:** 1; Arg520(2.70) **Carbon HB:** 1; Asp777(2.88)	**Pi-Alkyl:** 3; Ala285(3.43), Arg520(5.11) **Pi-Cation:** 1; Lys776(4.28)		
Tyrosine	**Conventional HB:** 2; Arg647(2.71), Glu767(1.97) **Carbon HB:** 1; Pro767(2.65)	**Pi-Pi T shaped:** 1; Tyr636(4.94) **Pi-Anion:** 1; Glu767(4.05)	**Salt Bridge Attractive Charge:** 4; Arg653(2.43), Arg653(1.87), Glu767(2.70), Glu767(2.52) **Attractive Charge:** 2; Arg647(4.10), Asp649(5.03)	**Unfavourable Donor-Donor:** 1; Arg647(1.80) **Unfavourable Acceptor-Acceptor:** 1; Ile734()
Maltose	**Conventional HB:** 6; Ile734(5.63), Arg647(6.41), Asp649(5.27), Glu658(5.02), Glu658(5.38), Tyr733(6.42) **Carbon HB:** 3; Glu767(3.18), Lys766(3.69) Lys765(5.40)			**Unfavourable Donor-Donor:** 1; Arg653(6.34)
4-Fluoro-3-[1‑hydroxy-2-(methylamino)ethyl] phenol	**Conventional HB:** 3; Arg520(2.54), Arg520(2.34), Arg520(2.03), **Carbon HB:** 3; Ser521(2.95), Phe535(2.42), Phe535(2.77)	**Pi-Alkyl:** 3; Ala285(4.32), Ala537(4.56), Pro287(5.21) **Pi-Sulfur:** 1; Met567(4.64)		**Unfavourable Positive-Positive:** 1; Lys776(5.15)
p-hydroxynorephedrine	**Conventional HB:** 1; Ile734(2.17),	**Alkyl:** Pro676(4.00) **Pi-Anion:** 1; Glu767(3.85) **Pi-Pi T shaped:** 1; Tyr636(5.03)		**Unfavourable Donor-Donor:** 1; Arg647(1.26)
Cathine	**Conventional HB:** 1; Arg520 (2.70) **Carbon HB:** 1; Asp777(2.88)	**Alkyl:** 1; Ala285(3.73) **Pi-Alkyl:** 3; Arg520 (5.10), Ala285(3.43) Lys776(4.69) **Pi-Cation:** 1; Lys776(4.22)		
L-Histidine	**Conventional HB:** 4; Asn543(4.13), His183(6.20), Trp552(4.46), Asp549(2.98) **Carbon HB:** 1; Ser553(4.17)	**Pi-alkyl:** 1; Leu540(5.60) **Pi-sigma:**1; Val184(3.85)	**Attractive charge:** 1; Asp549(3.75)	**Unfavorable Negative-Negative:** 1; Asp549(5.98)
Gluconic acid	**Conventional HB:** 3; Gln92(2.33), Phe119(2.02), Gln117(2.45) **Carbon HB:** 1; His98(2.83), Asp261(2.61)	**Pi-Anion:** 1; His98(3.41)		
L-Tryptophan	**Conventional HB:** 1; Arg653(2.27)	**Pi-Alkyl:** 2; Pro676(4.43), Pro676(4.86) **Pi-Anion:** 1; Glu767(3.50)	**Attractive charge:** 2; Arg647(2.96), Arg653(4.88) **Salt- Bridge Attractive charge:** 1; Glu658(3.01)	
L-phenylephrine	**Conventional HB:** 4; Arg526(2.39), Asp542(2.16), Asp542(2.65), His600(2.36),	**Pi-Anion:** 1; Asp443(3.84)		
Synephrine	**Conventional HB:** 3; Glu767(2.00), Glu767(2.06), Glu767(2.50)	**Pi-Anion:** 1; Glu767(3.86)		
Pyrido[3,4-d] imidazole, 1,6-dicarboxylic acid	**Conventional HB:** 4; Ser395(2.68), Ser395(2.20), Asp396(2.57), Ser459(2.07) **Carbon HB:** 1; Cys458(2.84)	**Pi-Alkyl:** 1; Leu401(5.46)		
2,5-Dimethoxy-4-ethylamphetamine	**Carbon HB:** 6; Gly753(2.64), Leu754(2.59), Glu815(2.72), Glu815(2.56), Thr632(2.75), Glu815(4.10)	**Alkyl:** 4; Ile755(4.88), Lys817(4.63), Pro740(4.50), Tyr703(4.94) **Pi-Anion:** 1; Ile755(5.47)		
1,2 O-Isopropylidene-alpha-d- glucofuranose	**Conventional HB:** 4; Asn14(2.39), Asn14(2.52), Ile16(2.57), Val41(2.07) **Carbon HB:** 1; Asn14(2.84)	**Alkyl:** 2; Pro42(3.82), Val244(4.93) **Pi-Alkyl:** 1; Trp194(4.69)		
Prosta 5,13‑dien-1-oic acid		**Alkyl:** 1; Ala576(4.46) **Pi-Alkyl:**7; Tyr299(5.12), Tyr299(5.11), Trp406(5.14), Trp406(5.43), Phe575(4.88), Phe575(4.91), Phe575(5.43)		
3-Ethoxy-4-hydroxymandelic acid	**Conventional HB:** 3; Thr737(2.11), Gln739(2.71), Glu815(2.85) **Carbon HB:** 2; Gln738(2.99), Gly753(2.81)	**Pi-Anion:** 1; Glu815(3.23)		
I-Guanidinosuccinimide	**Conventional HB:** 3; Phe535(2.28), Arg520(2.03), Ser521(2.87) **Carbon HB:** 1; Ala536(2.99),			
D-Fructopyranose	**Conventional HB:** 3; Leu752(1.97), Asn814(2.32), Asn814(2.20) **Carbon HB:** 2; Gln738(2.96), Glu815(3.07)			
Gulonic acid	**Conventional HB:** 6; Glu333(2.74), Arg334(2.35), Asp329(2.72), Asp343(1.80), Glu300(2.45), Gly302(3.06) **Carbon HB:**2; Gly302(3.06), Glu300(2.81)		**Attractive charge:** 1; Arg334(4.69)	**Unfavourable Donor-Donor:** 1; Glu333(1.25) **Unfavourable Negative- Negative:** 1; Glu333(4.86) **Unfavourable Acceptor- Acceptor:** 1; Asp343(2.82)
5-(2-Aminopropyl)−2-methylphenol	**Conventional HB:** 1; Asp348(2.47)	**Pi-Alkyl:** 2; Lys519(5.26), His432(4.50) **Pi-Cation:** 1; Phe516(4.14) **Pi - Pi T shaped:** 1; Phe516(4.75)	**Attractive charge:** 1; Asp348(4.28) Phe516()	**Unfavourable Donor-Donor:** 1; Ser521(2.32)
11-Eicosenoic acid	**Conventional HB:** 1; Gln603(2.05) **Carbon HB:** 1; Gly602(2.72)	**Pi-alkyl:** 10; Tyr299(5.00), Tyr299(4.21), Trp406(4.66), Trp406(5.45), Trp406(4.96), Trp406(4.69), Phe450(5.41), Phe450(4.32), Phe450(4.96), Phe575(4.87)		
Mannoic acid	**Conventional HB:** 4; Ser456(3.05), Ser395(2.49), Sr459(2.22), Asp485(2.79) **Carbon HB:** 3; Cys458(2.66), Ser454(2.71), Ser456(2.65)			**Unfavorable Negative-Negative:** 1; Asp485(4.56) **Unfavorable Acceptor-Acceptor:** 1; Ser394(2.82)
2- Butendioic acid	**Conventional HB:** 2; Phe535(6.41), Ile523(3.68) **Carbon HB:** 1; Pro287(3.81)			
Heptanedioic acid	**Conventional HB:** 3; Asn14(2.55), Arg254(2.25), Asn14(2.11) **Carbon HB:** 1; Pro17(2.92)	**Alkyl:** 1; Pro42(4.63) **Pi-Anion:** 1; Trp43(4.89)	**Attractive charge:** 2; Arg254(3.97) Arg471(3.18)	
L- Alanine ethylamide (S)	**Conventional HB:** 2; Thr821(3.46), Thr821 (3.61)	**Alkyl:** 4; Lys762(4.22), Leu758(4.90), Lys762(5.09), Val791(4.94) **Pi-alkyl:** 3; Trp868(5.22), Trp868(4.94), His870(3.99)		
10-Octadecenoic acid	**Conventional HB:** 2; Trp43(4.84), Ser40(4.14)	**Alkyl:** 4; Pro21(4.89), Pro21(5.14), Pro42(4.89), Val244(4.79) **Pi-Alkyl:** 1; Trp194(5.13) **Pi-Sigma:** 1; Trp43(2.46)		
2-Propenoic acid, n-pentadecyl ester		**Alkyl:** 1; Leu127(4.80) **Pi-alkyl:** 3; Tyr91(5.23), Phe65(5.10), Phe65(4.56)

**Fig. 1:**
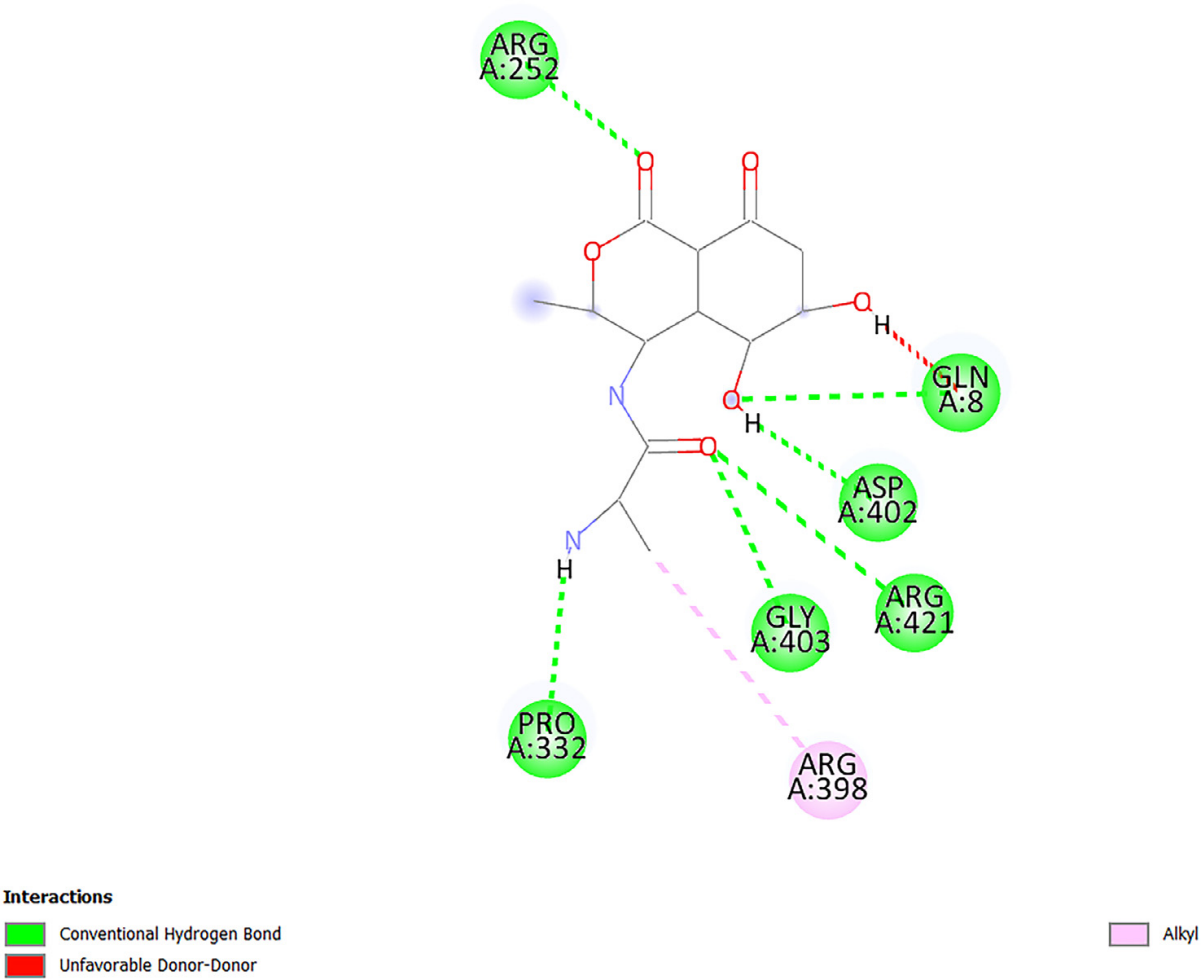
Two-dimensional interaction diagram of Actinobolin with the alpha-amylase enzyme (PDB ID: 2QMK), illustrating key binding interactions, including hydrogen bonds, hydrophobic contacts, and ionic interactions. The figure highlights the specific residues of alpha-amylase involved in the interaction, providing insights into the binding mechanism of Actinobolin and its potential inhibitory effects on enzymatic activity.

**Fig. 2:**
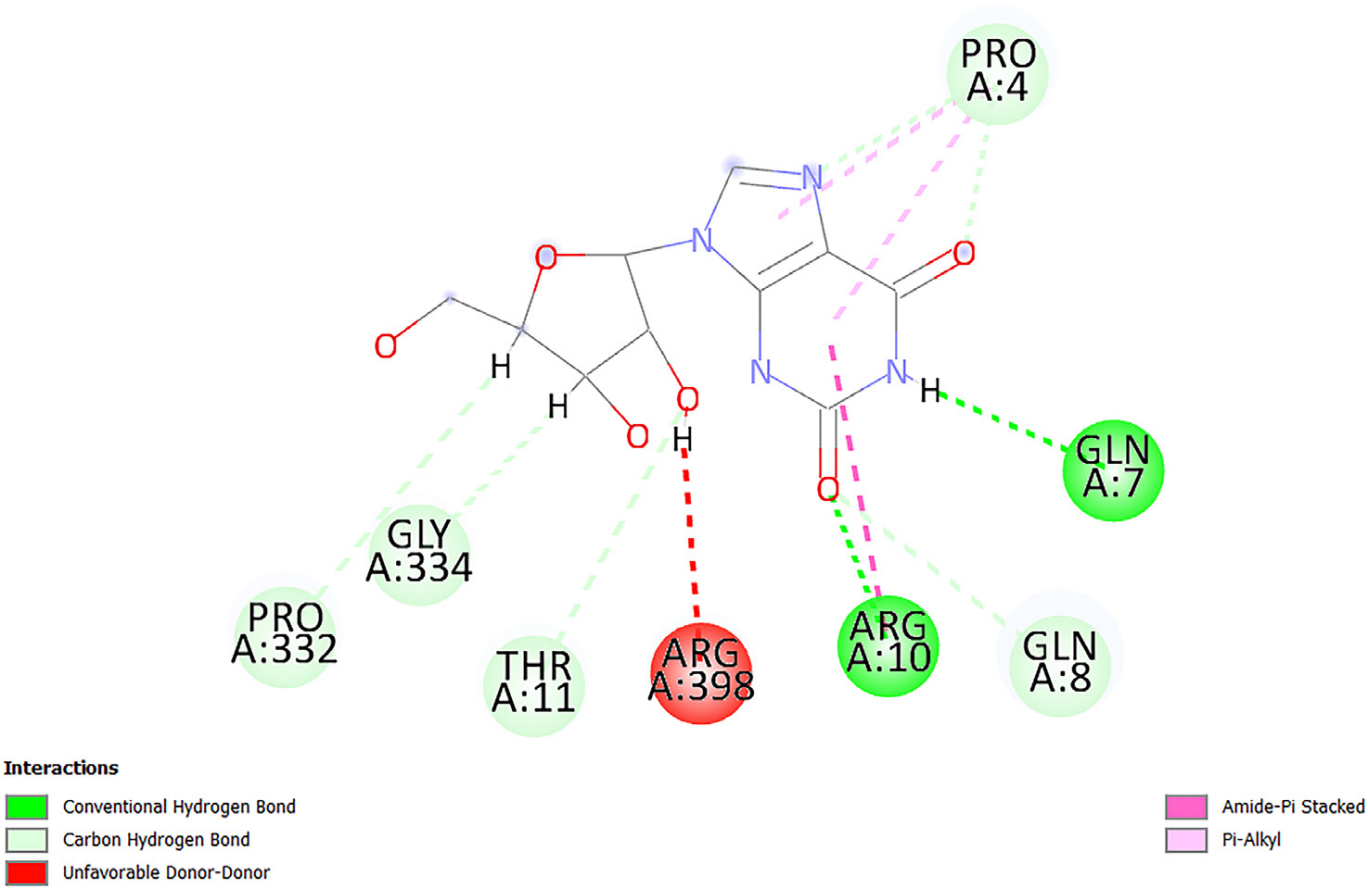
Two-dimensional interaction diagram of Xanthosine with the alpha-amylase enzyme (PDB ID: 2QMK), depicting the molecular interactions such as hydrogen bonding and van der Waals forces. This figure also identifies the active site residues interacting with Xanthosine, emphasizing its role in modulating enzymatic activity.

**Fig. 3:**
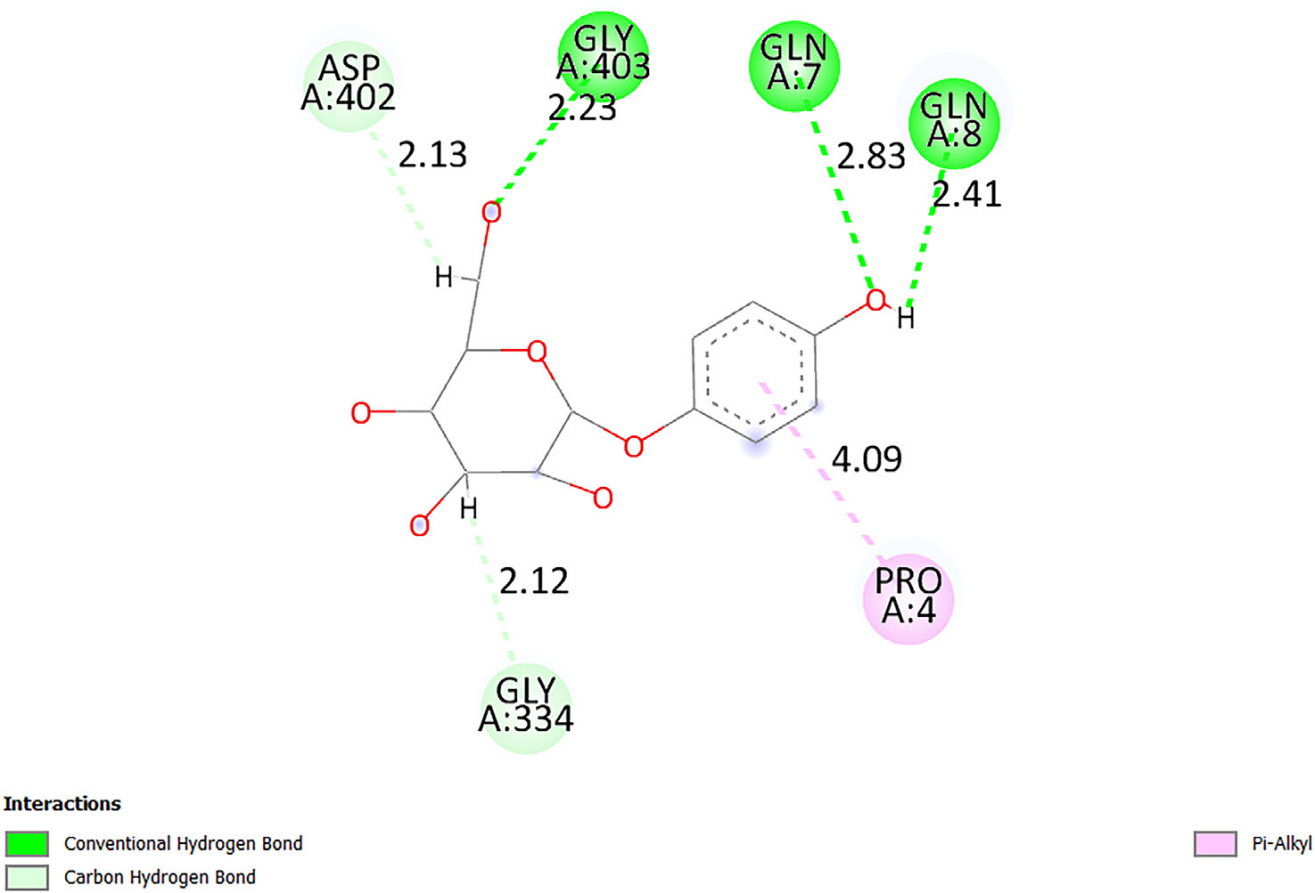
Two-dimensional interaction diagram of Arbutin with alpha-amylase (PDB ID: 2QMK), showing the interaction landscape, including hydrophobic pockets and polar residues. The visualization provides a clear understanding of Arbutin's binding affinity and specificity towards the enzyme.

**Fig. 4:**
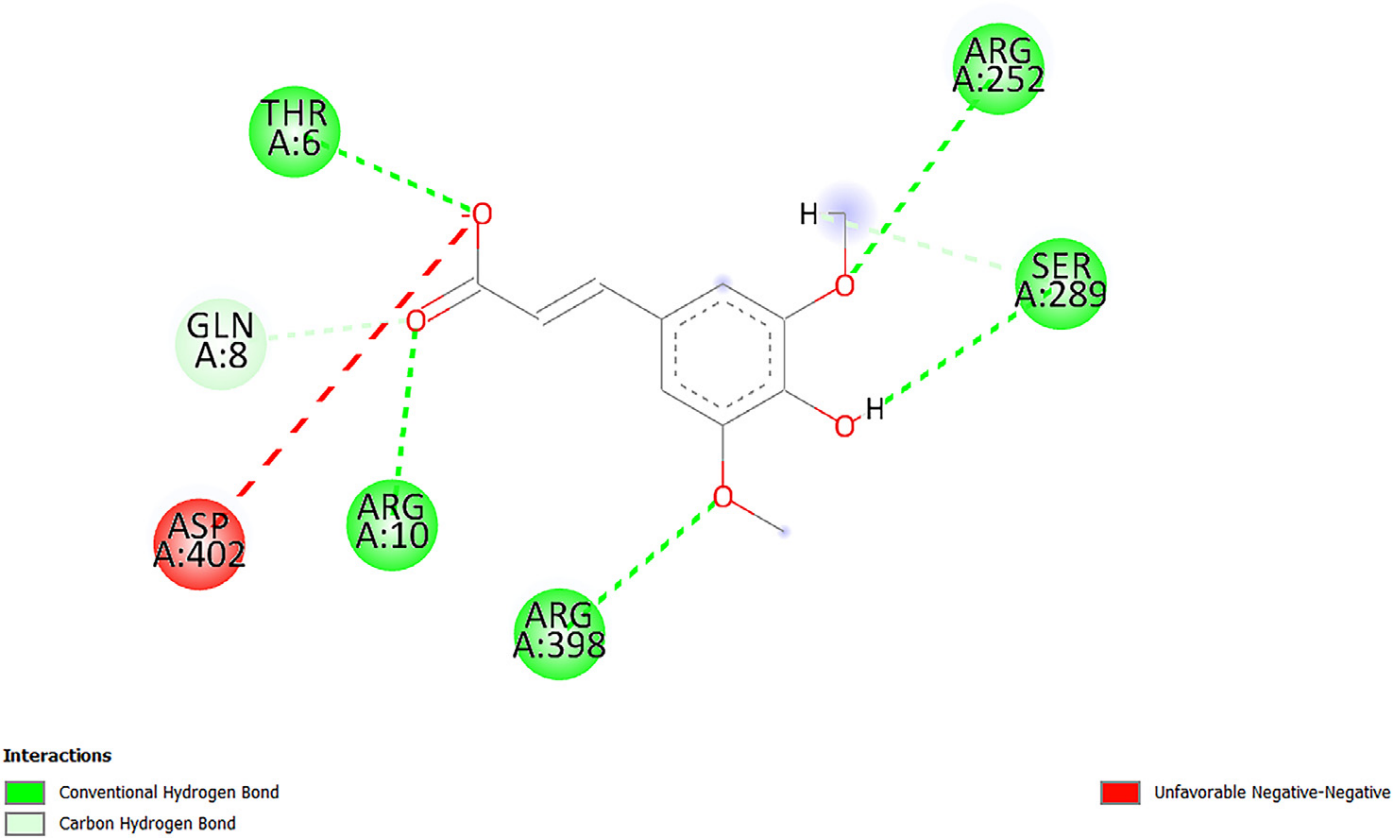
Two-dimensional interaction diagram of Sinapic acid with alpha-amylase (PDB ID: 2QMK). The figure details the types of interactions, including hydrophobic and hydrogen bonds, and their contribution to the binding energy and specificity of Sinapic acid.

**Fig. 5:**
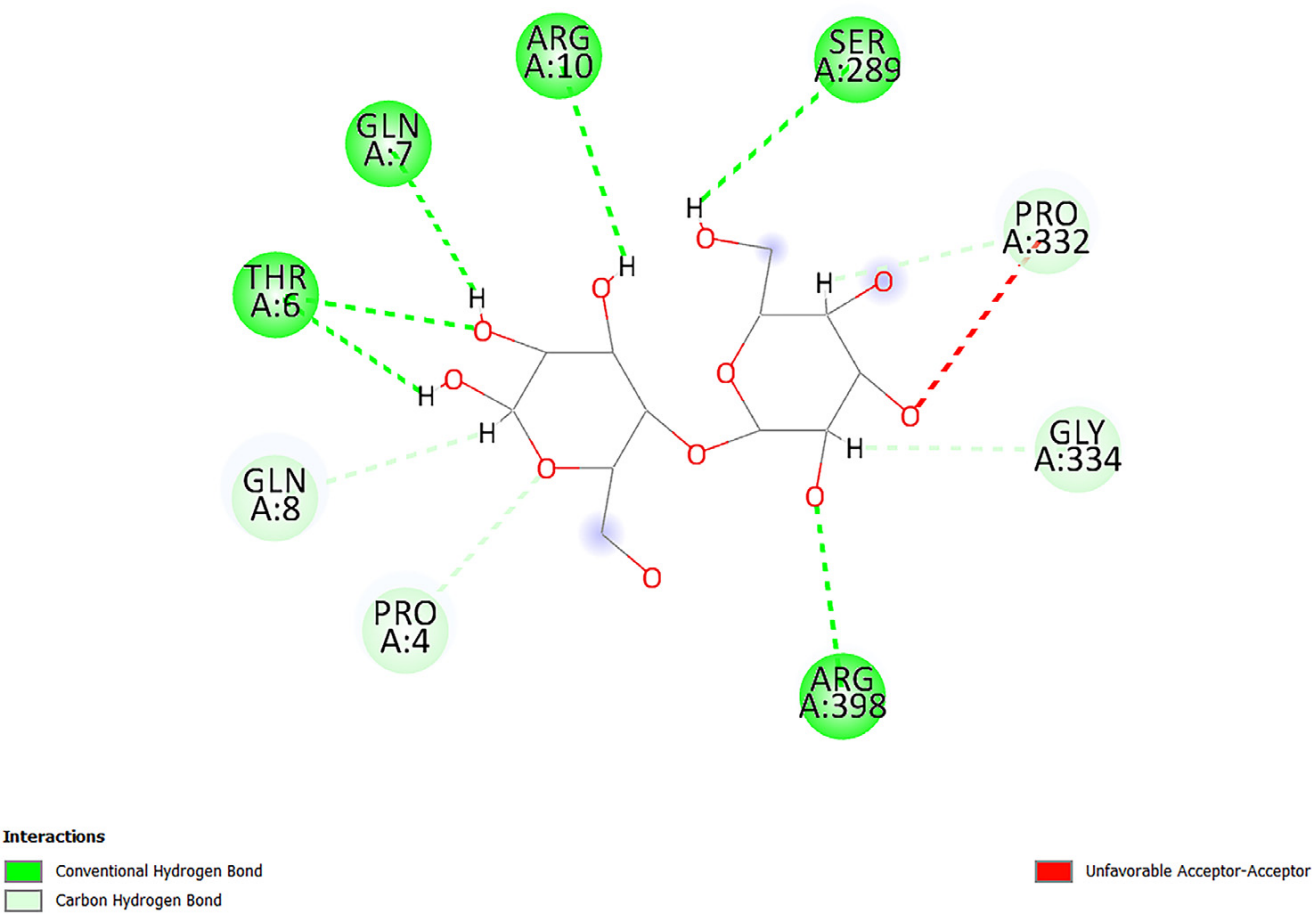
Two-dimensional interaction diagram of Maltose with alpha-amylase (PDB ID: 2QMK). This figure highlights the interaction network within the active site, revealing how Maltose aligns with the catalytic residues to facilitate enzymatic hydrolysis.

**Fig. 6:**
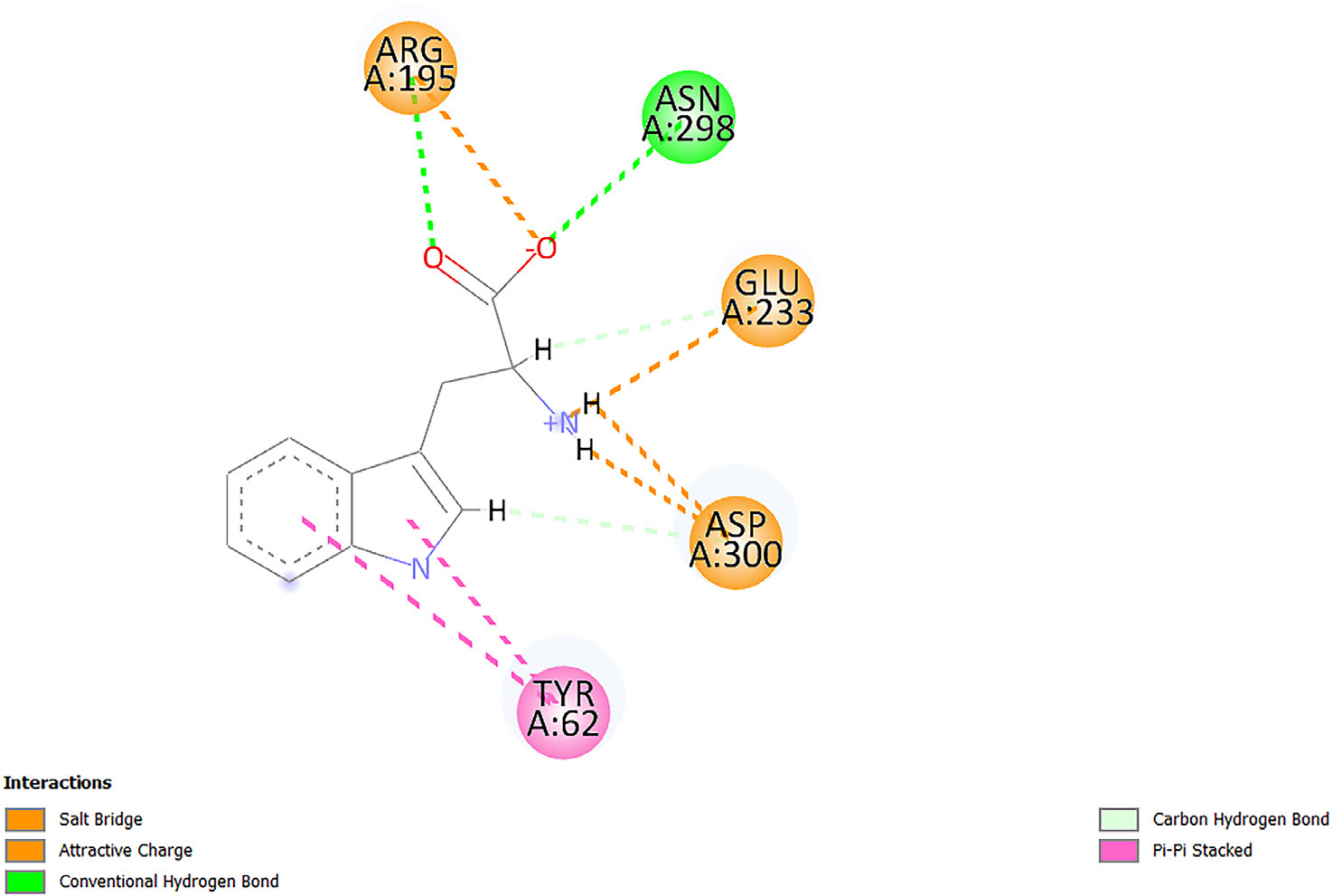
Two-dimensional interaction diagram of L-Tryptophan with alpha-amylase (PDB ID: 2QMK). The depiction includes key interactions, such as aromatic stacking and hydrogen bonds, explaining l-Tryptophan's potential inhibitory or modulatory role.

**Fig. 7:**
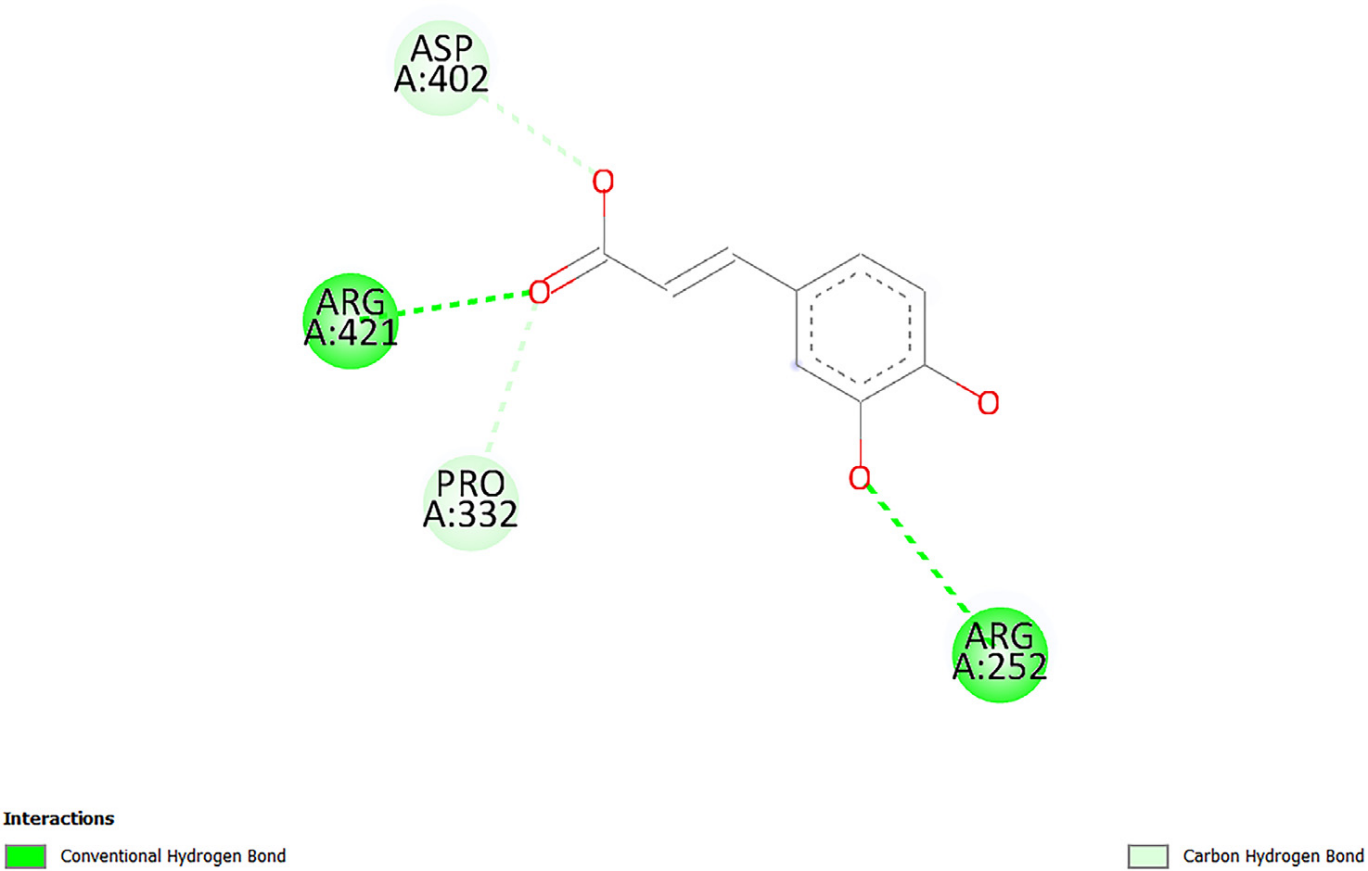
Two-dimensional interaction diagram of Caffeic acid with alpha-amylase (PDB ID: 2QMK), showing the binding interface and highlighting critical residues that stabilize the interaction. This provides insight into the role of Caffeic acid in influencing enzyme function.

**Fig. 8:**
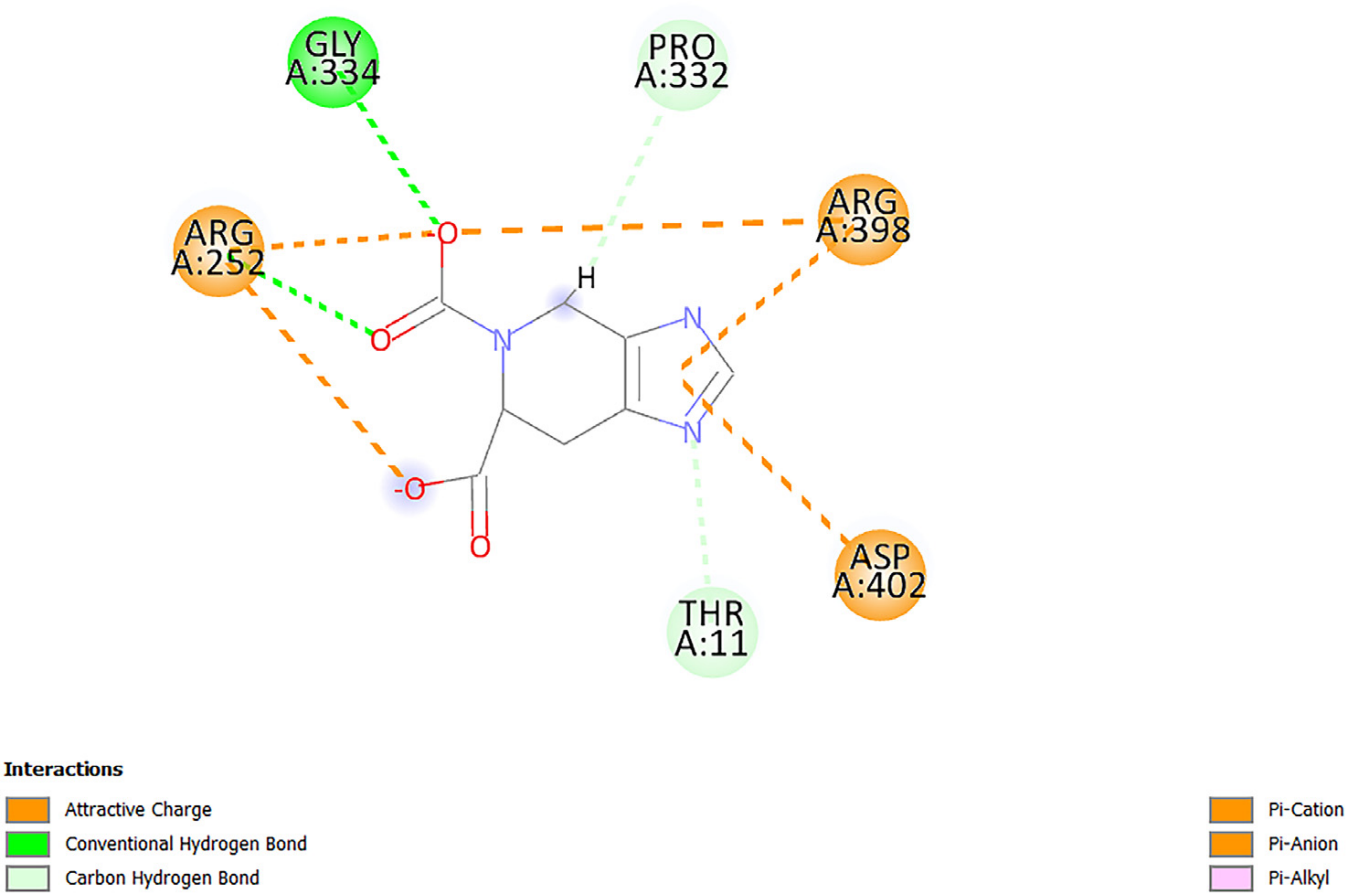
Two-dimensional interaction diagram of Pyrido[3,4-d]imidazole, 1,6-dicarboxylic acid with alpha-amylase (PDB ID: 2QMK). The figure illustrates the molecular interaction profile, emphasizing ionic bonds and hydrogen bonding networks.

**Fig. 9:**
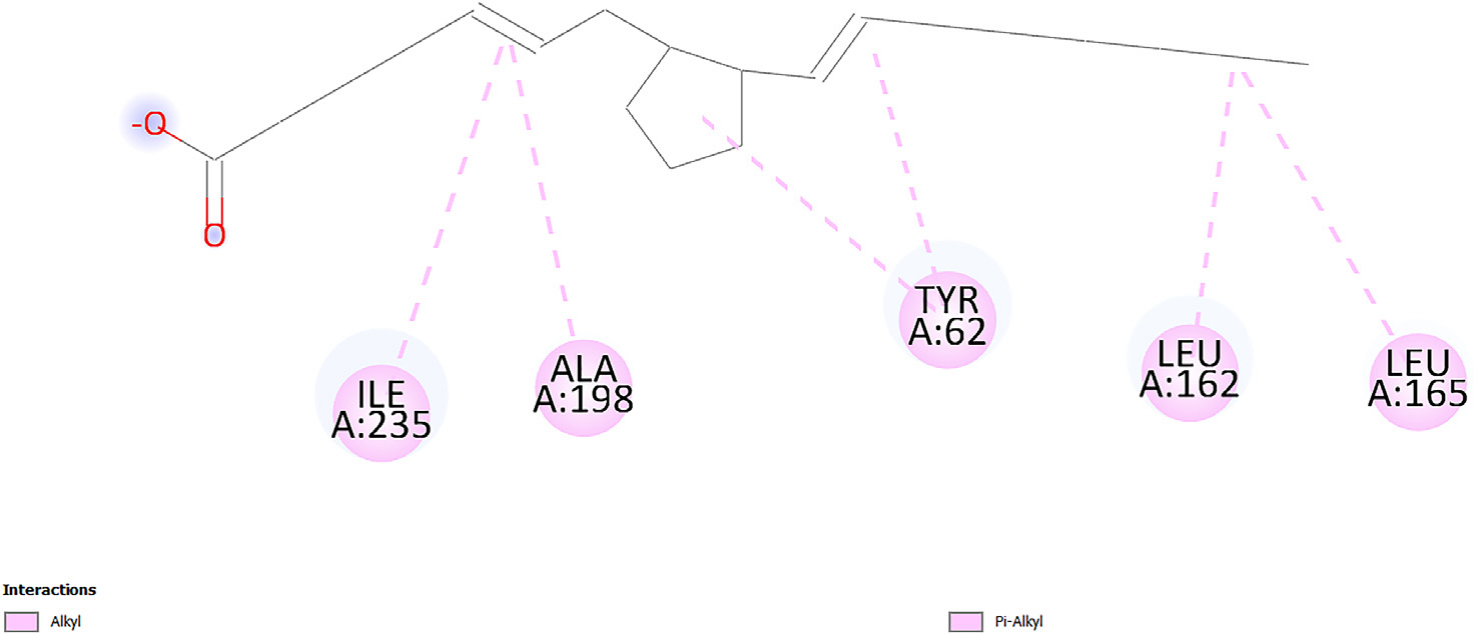
Two-dimensional interaction diagram of Prosta 5,13‑dien-1-oic acid with alpha-amylase (PDB ID: 2QMK). The representation focuses on the interaction hotspots within the enzyme's binding pocket, providing insights into Prosta 5,13‑dien-1-oic acid's potential as a ligand.

**Fig. 10:**
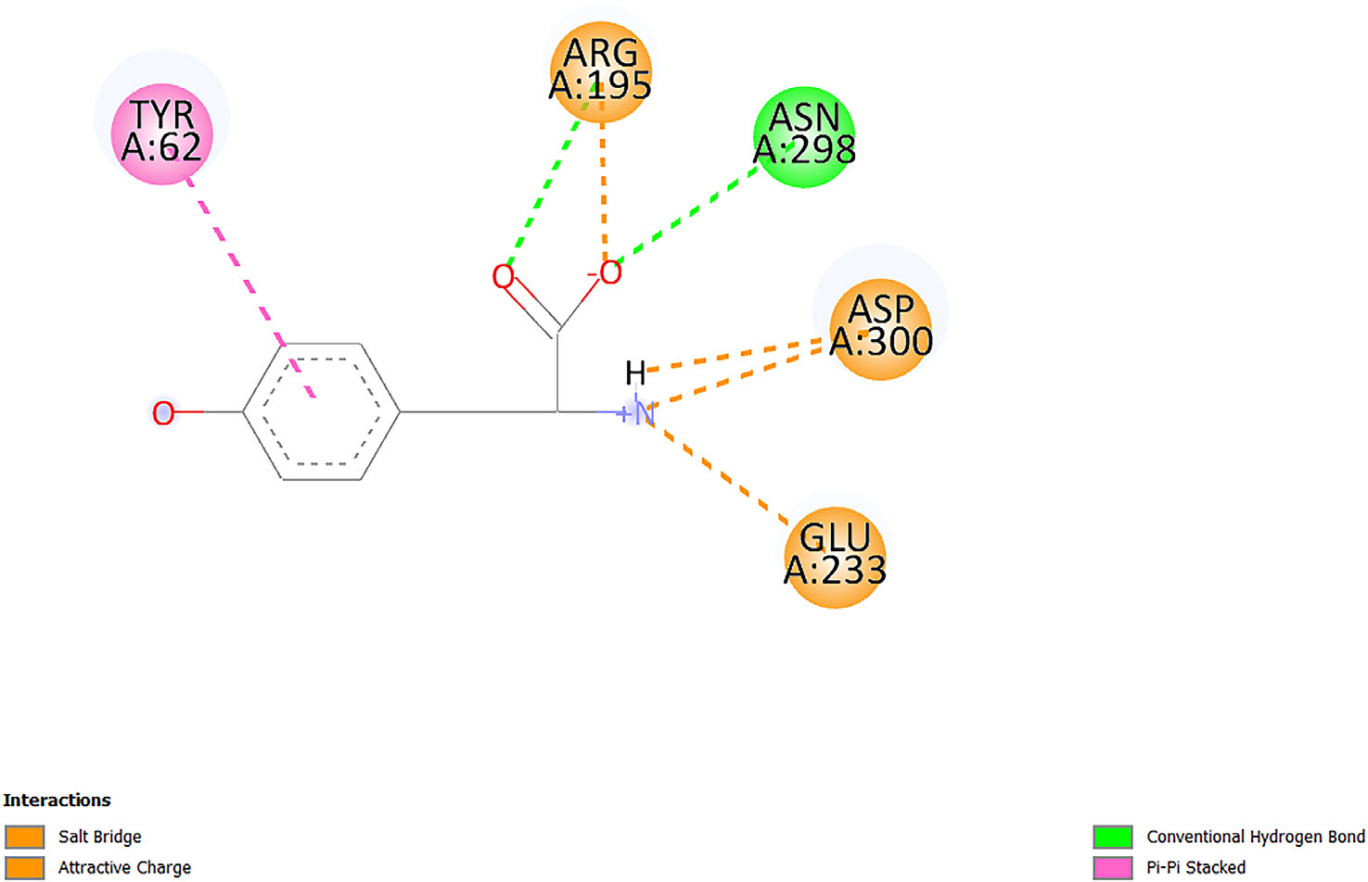
Two-dimensional interaction diagram of L-Tyrosine with alpha-amylase (PDB ID: 2QMK). The figure highlights specific interactions, including hydrogen bonds and hydrophobic contacts, that contribute to L-Tyrosine's binding affinity.

**Fig. 11:**
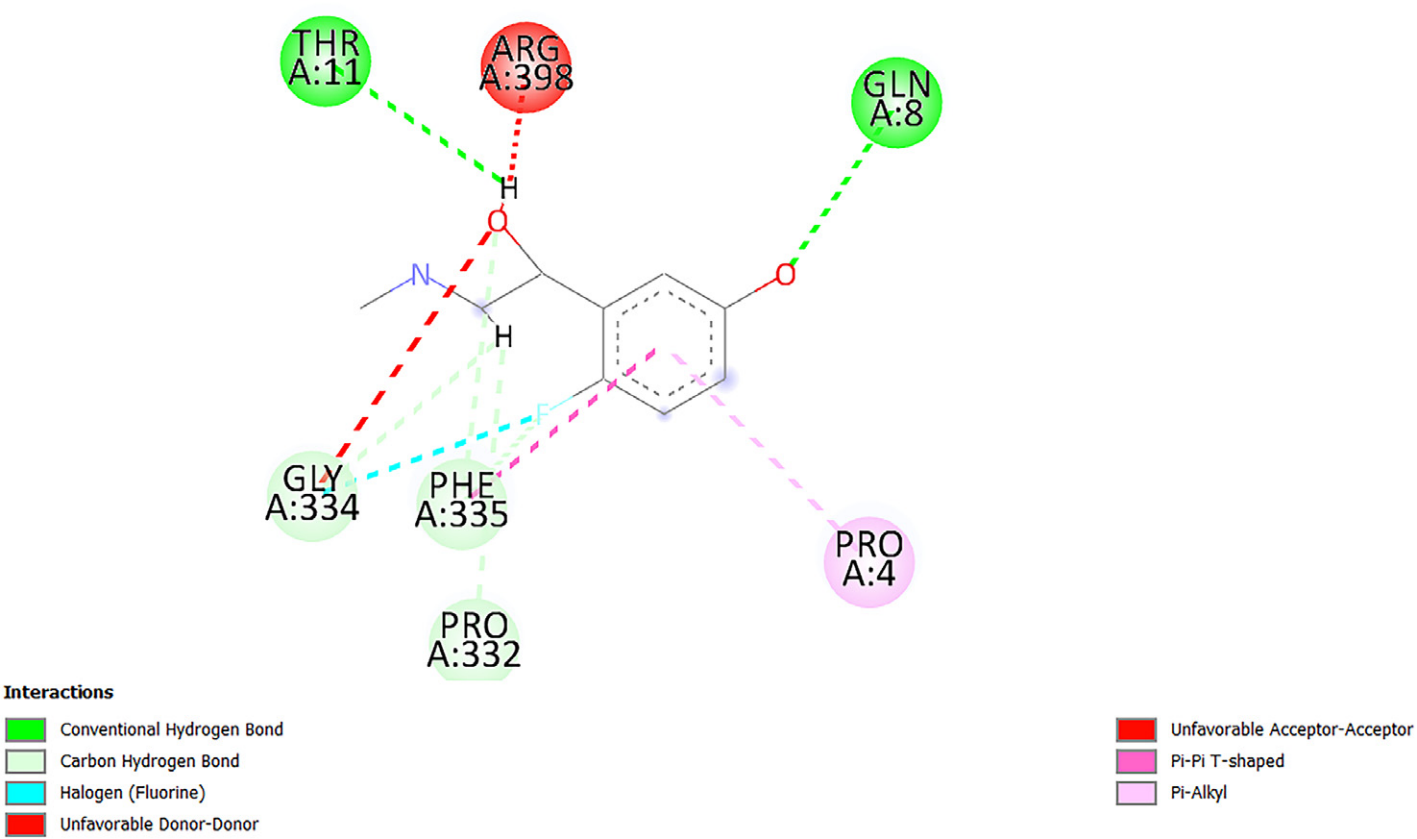
Two-dimensional interaction diagram of 4-Fluoro-3-[1‑hydroxy-2-(methylamino)ethyl] phenol with alpha-amylase (PDB ID: 2QMK). The interactions depicted include hydrogen bonds and polar contacts, providing insight into its potential enzymatic inhibition.

**Fig. 12:**
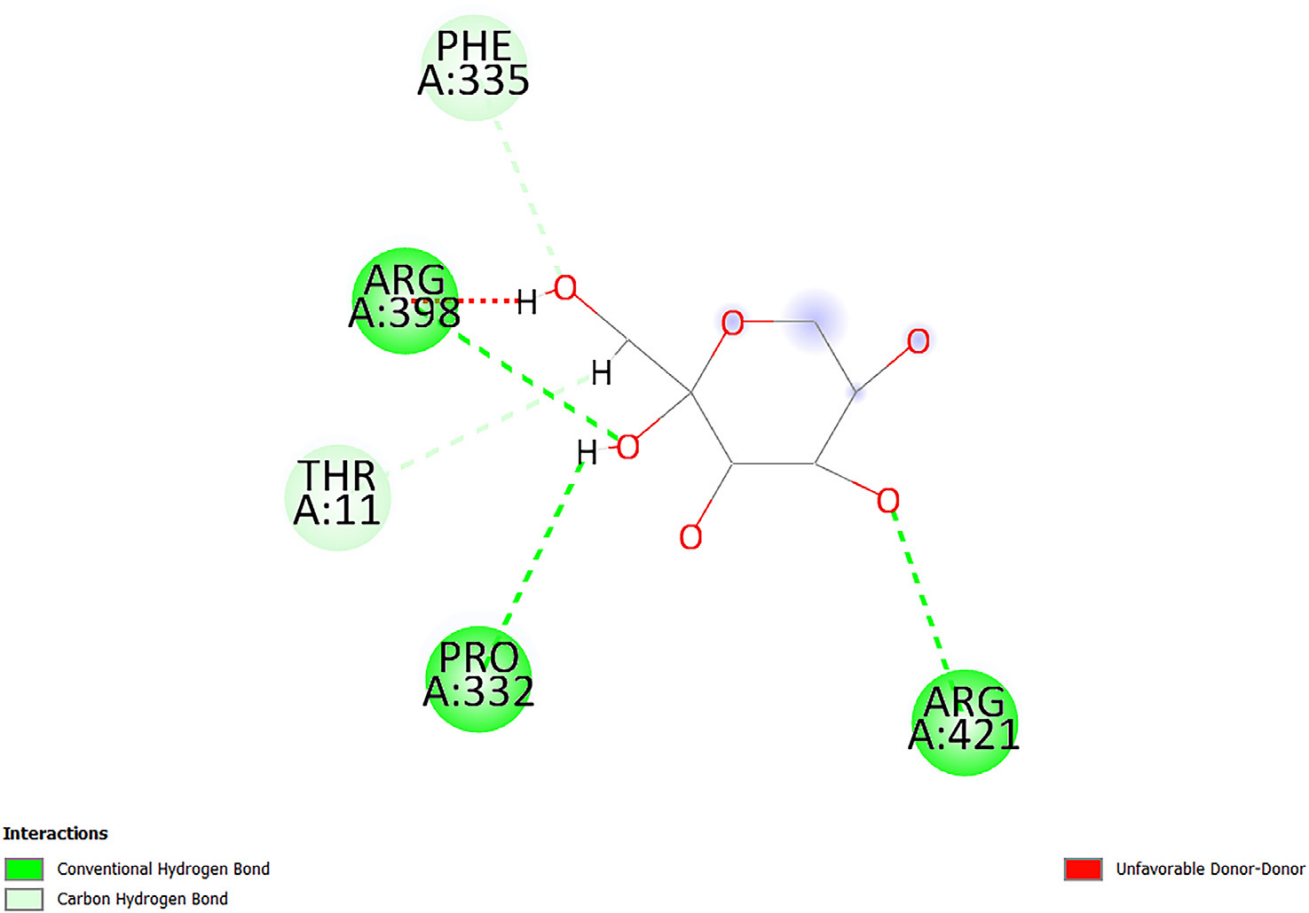
Two-dimensional interaction diagram of D-Fructopyranose with alpha-amylase (PDB ID: 2QMK), focusing on the active site interactions that facilitate carbohydrate recognition and hydrolysis.

**Fig. 13:**
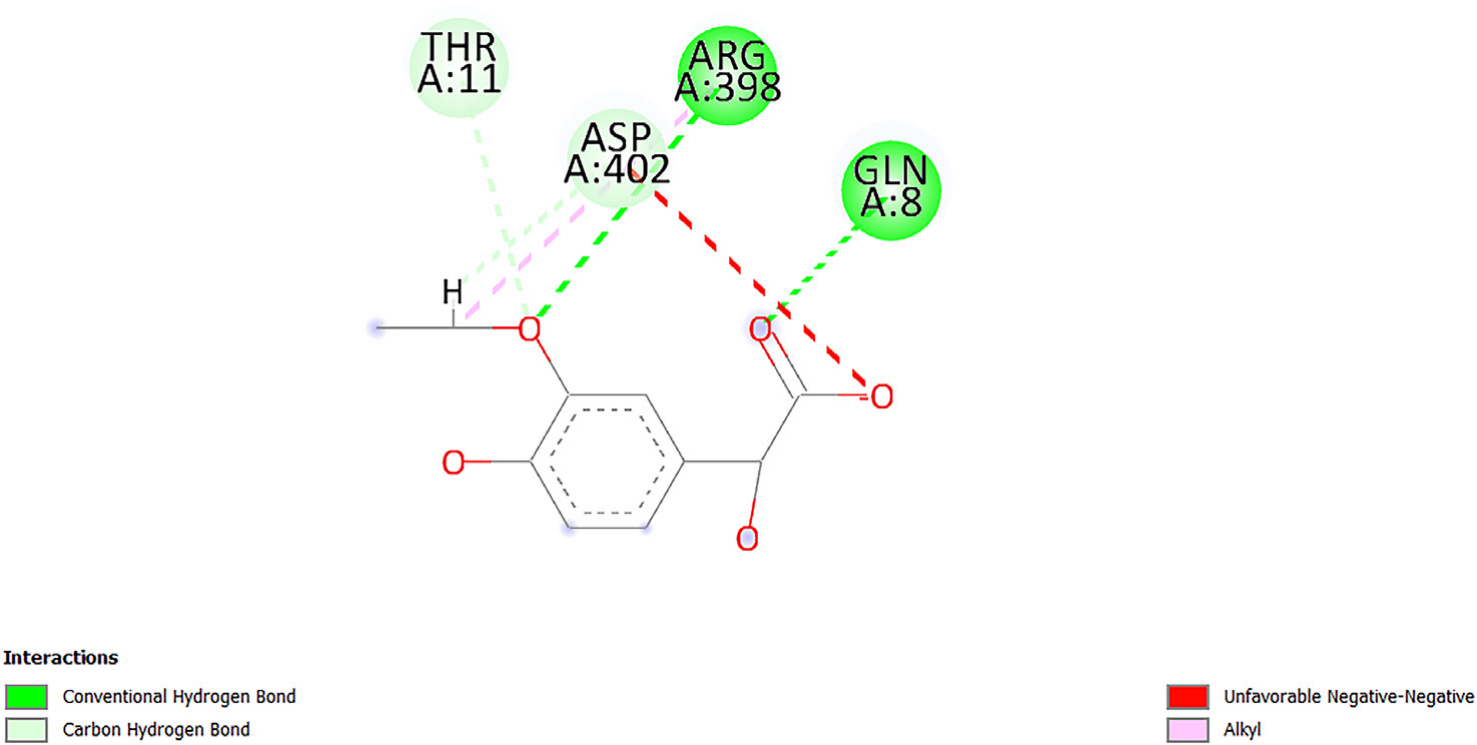
Two-dimensional interaction diagram of 3-Ethoxy-4-hydroxymandelic acid with alpha-amylase (PDB ID: 2QMK), illustrating the molecular binding landscape and key residues involved in the interaction.

**Fig. 14:**
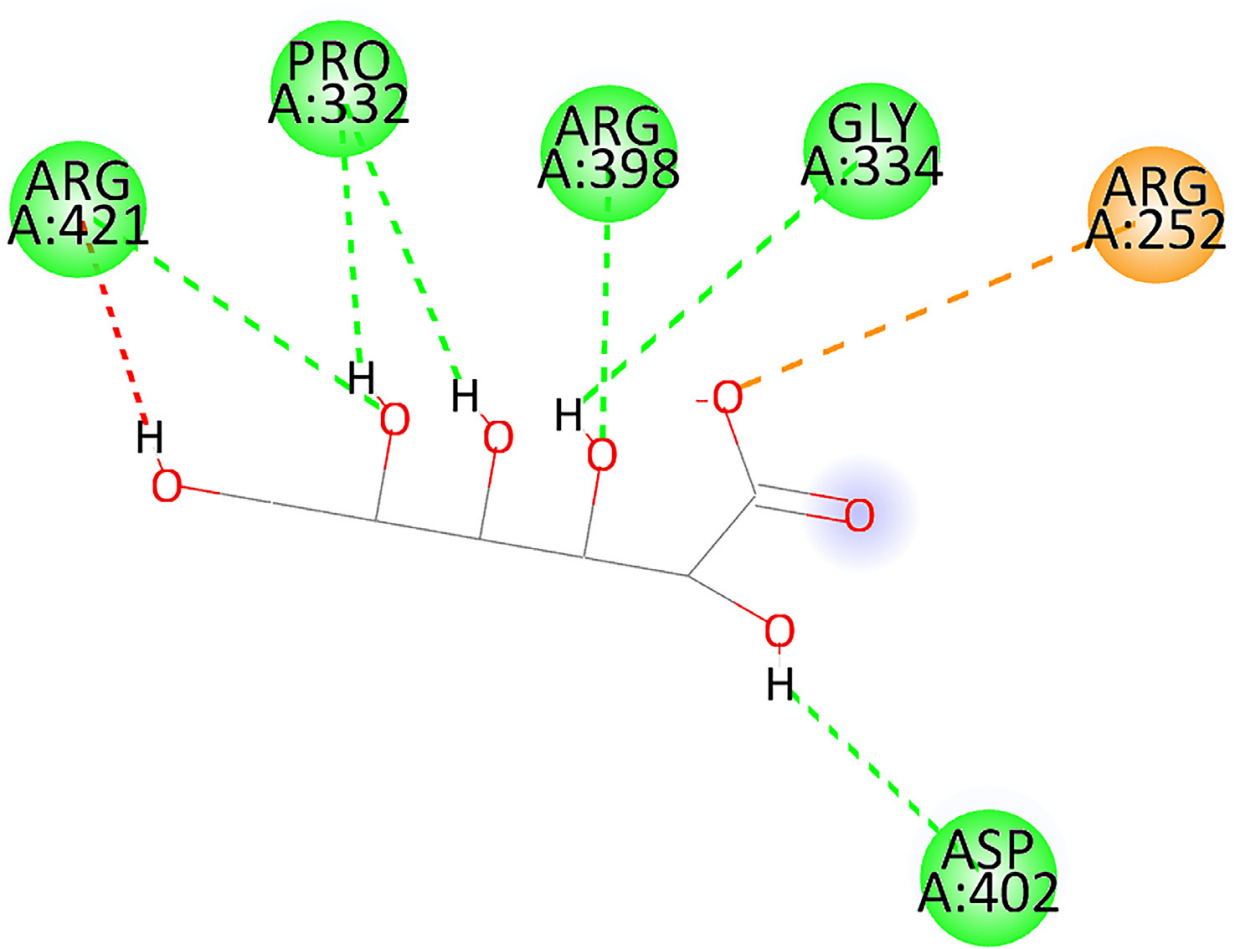
Two-dimensional interaction diagram of Gluconic acid with alpha-amylase (PDB ID: 2QMK). The figure highlights the interaction features, including hydrogen bonding and ionic interactions, within the enzyme's active site.

**Fig. 15:**
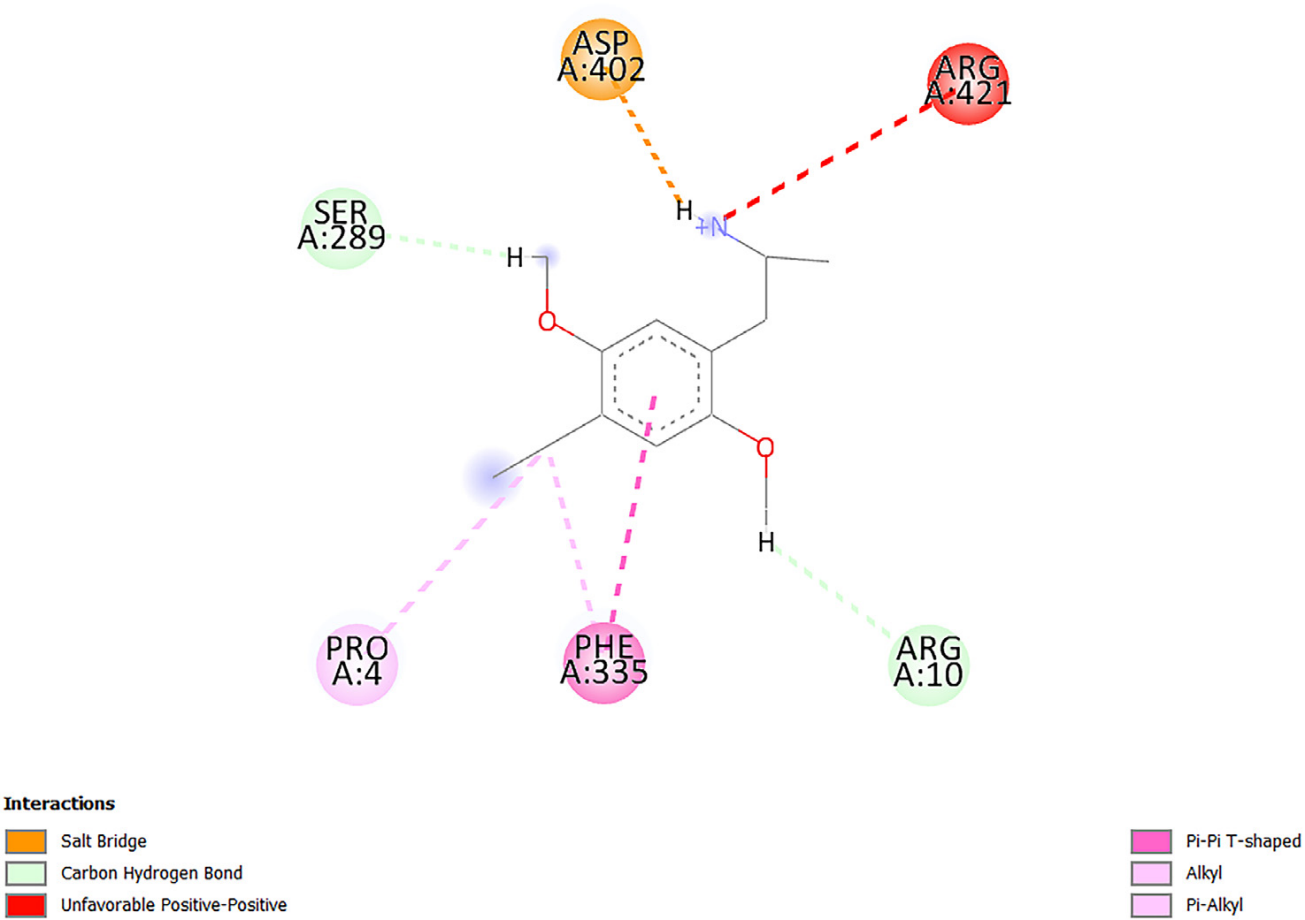
Two-dimensional interaction diagram of 2,5-Dimethoxy-4-ethylamphetamine with alpha-amylase (PDB ID: 2QMK). The binding interactions depicted highlight the molecular features that may influence enzymatic activity.

**Fig. 16:**
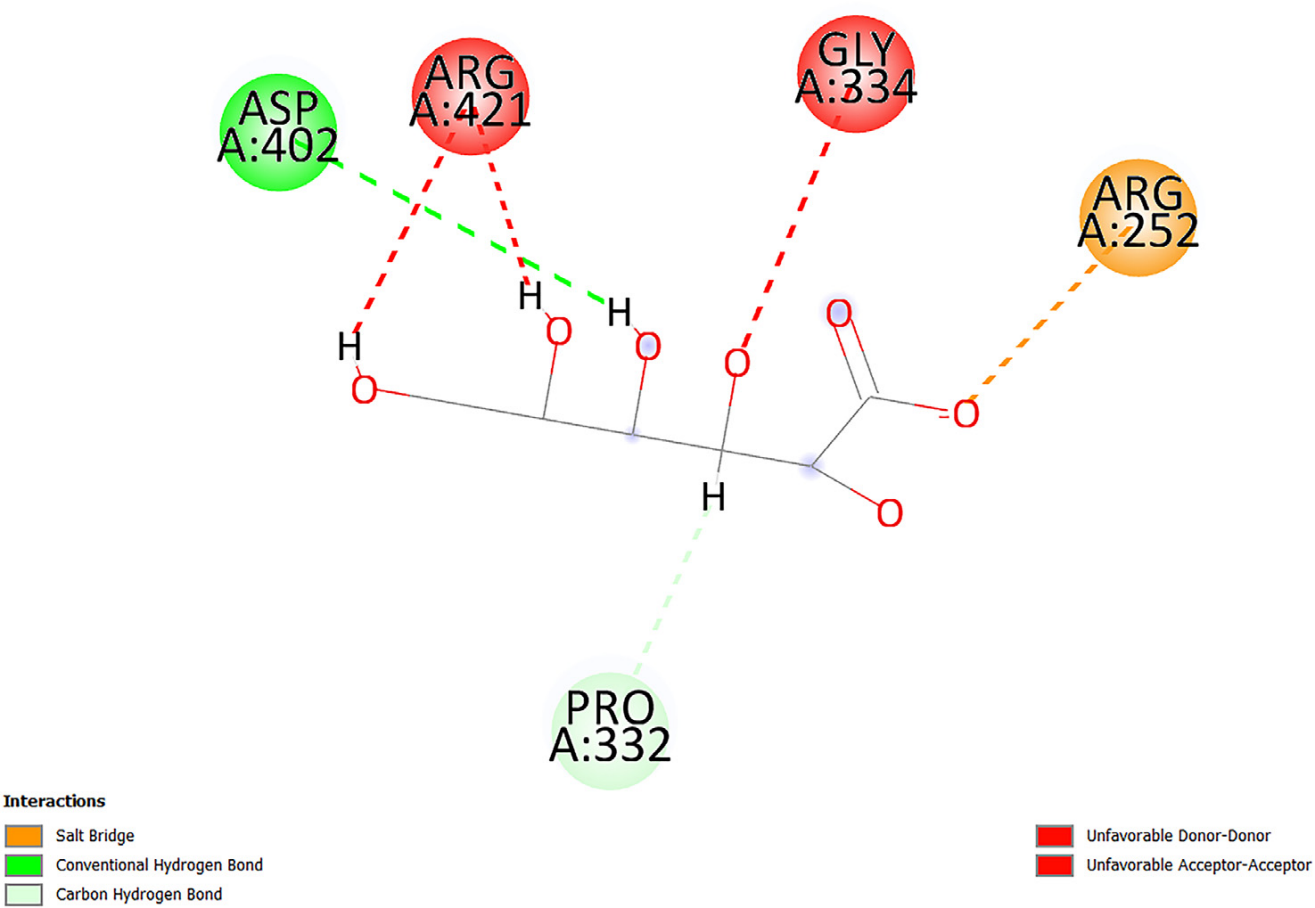
Two-dimensional interaction diagram of Gulonic acid with alpha-amylase (PDB ID: 2QMK), focusing on the interaction dynamics and their implications for enzyme-ligand affinity.

**Fig. 17:**
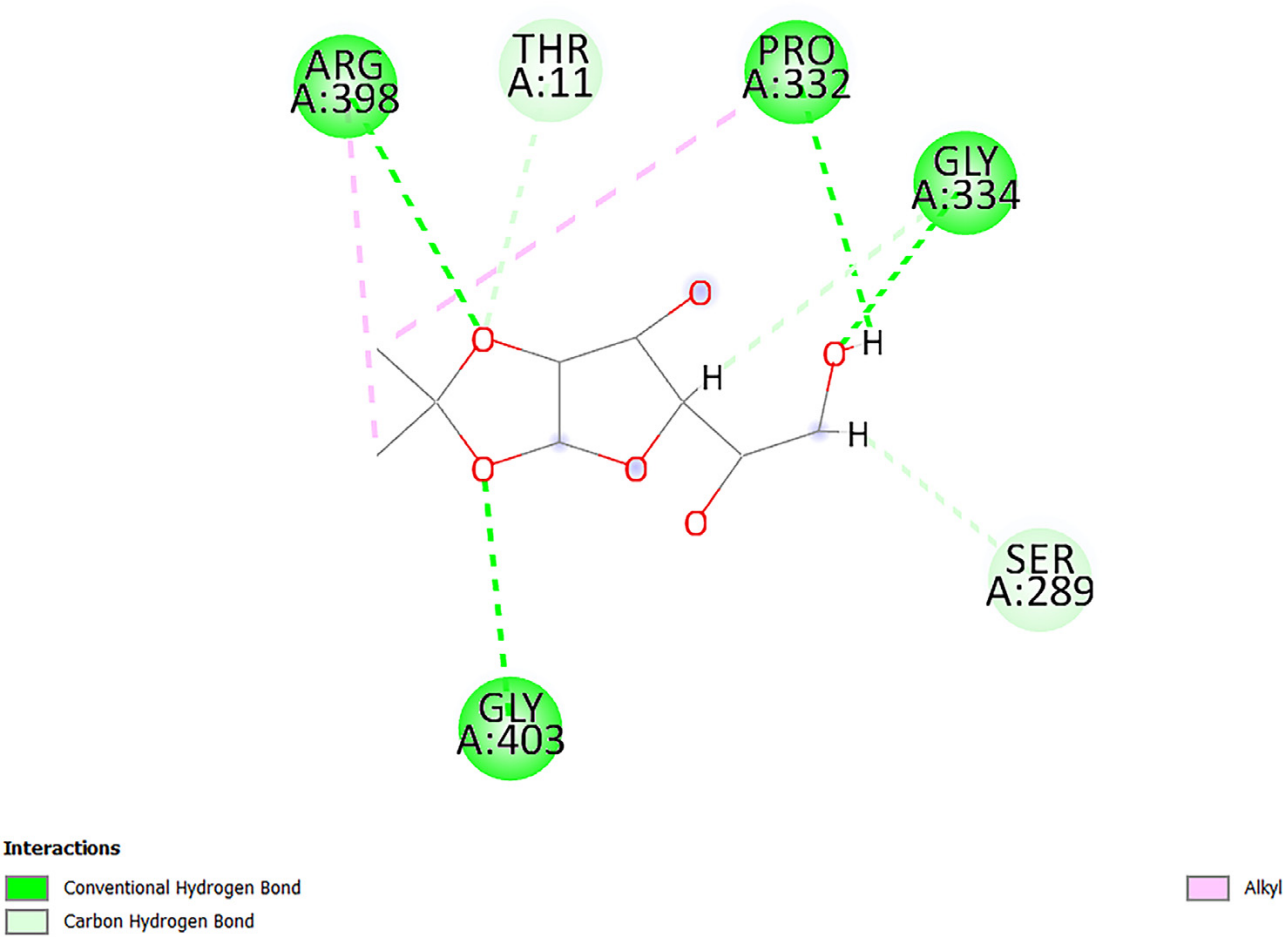
Two-dimensional interaction diagram of 1,2 O-Isopropylidene-alpha-D-glucofuranose with alpha-amylase (PDB ID: 2QMK). This figure emphasizes the role of hydrogen bonds and hydrophobic contacts in stabilizing the ligand within the binding pocket.

**Fig. 18:**
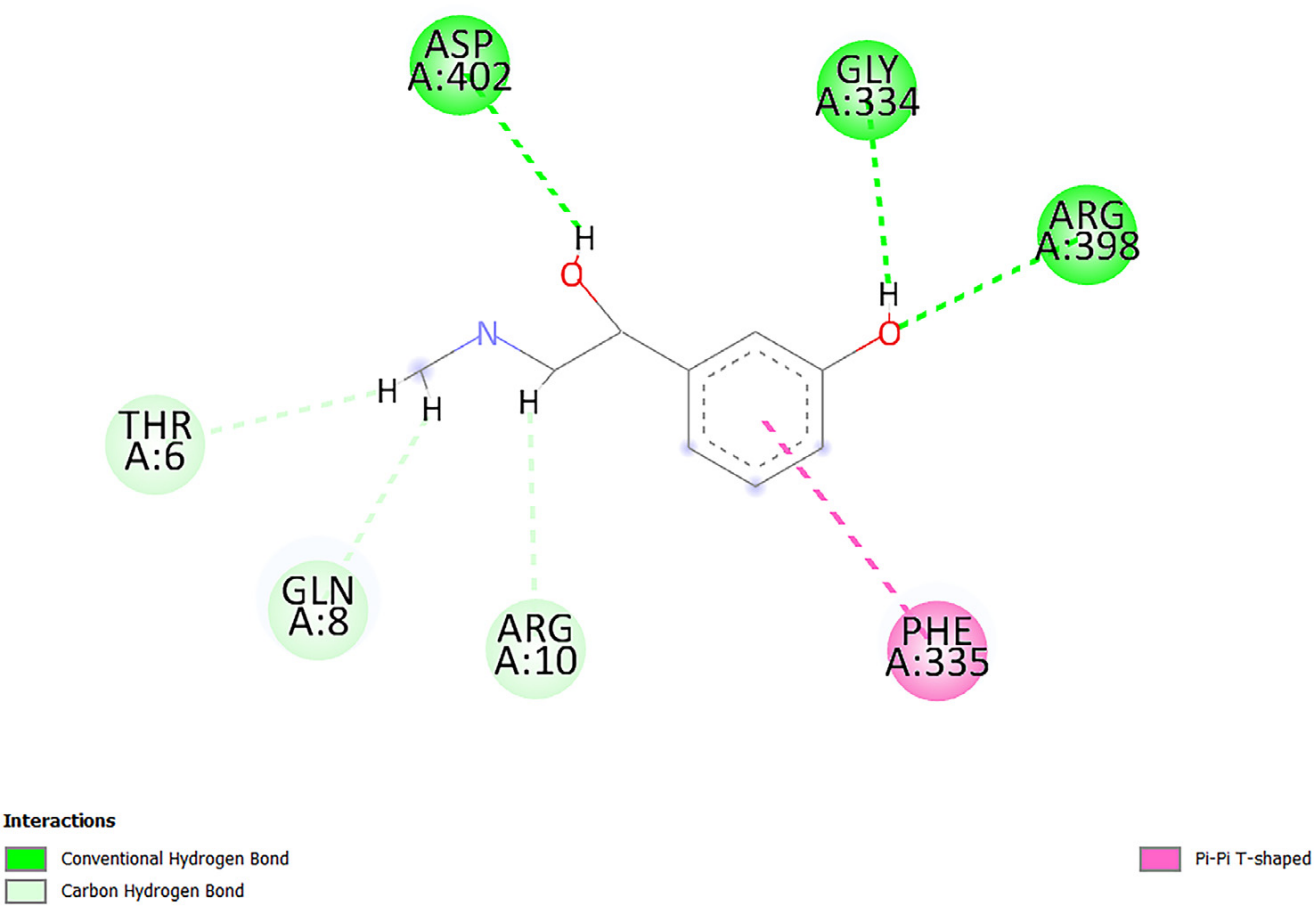
Two-dimensional interaction diagram of Phenylphrine with alpha-amylase (PDB ID: 2QMK), illustrating the structural basis for the interaction and its potential effects on enzymatic activity.

**Fig. 19:**
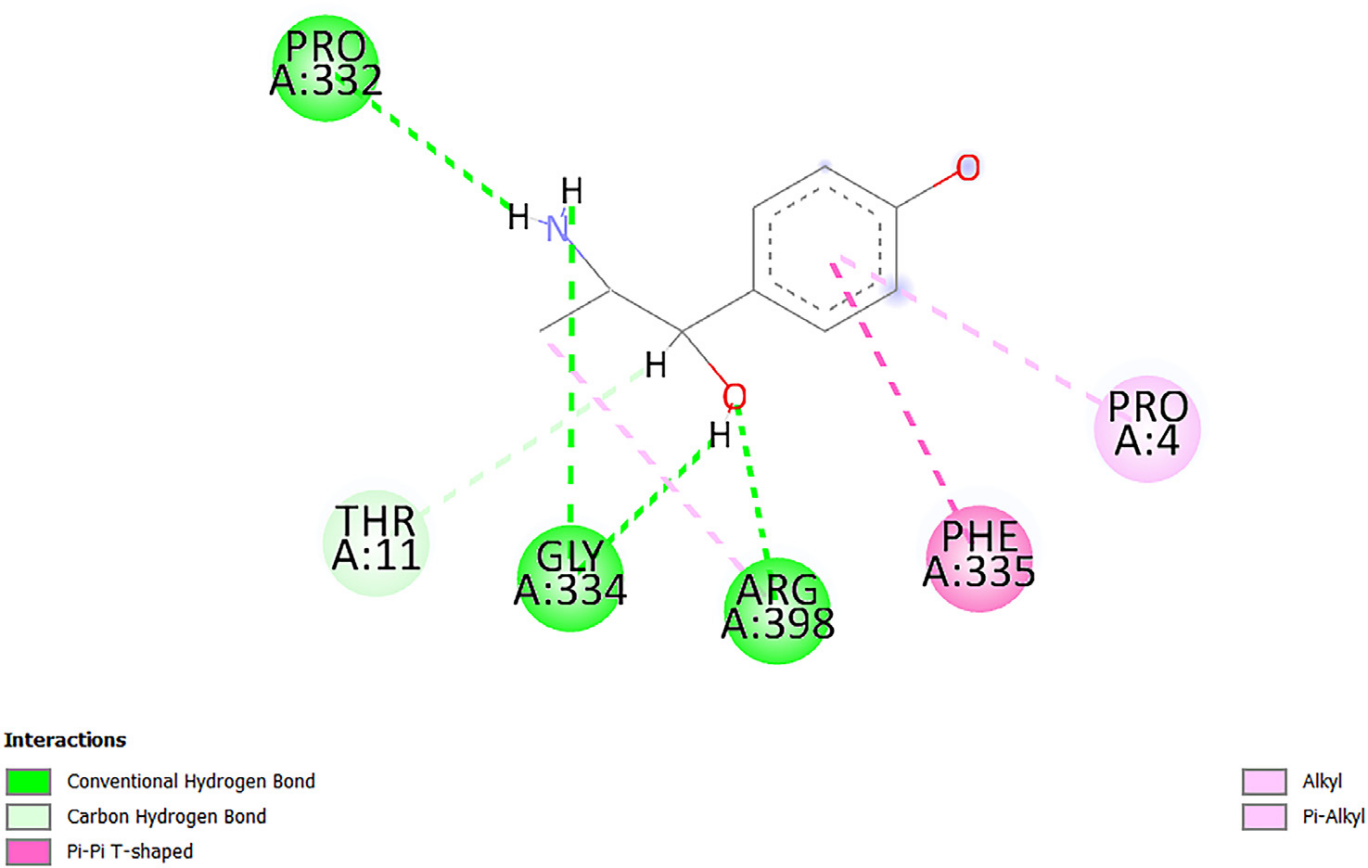
Two-dimensional interaction diagram of p-Hydroxynorephedrine with alpha-amylase (PDB ID: 2QMK), showing detailed interaction features, including ionic and hydrogen bonding.

**Fig. 20:**
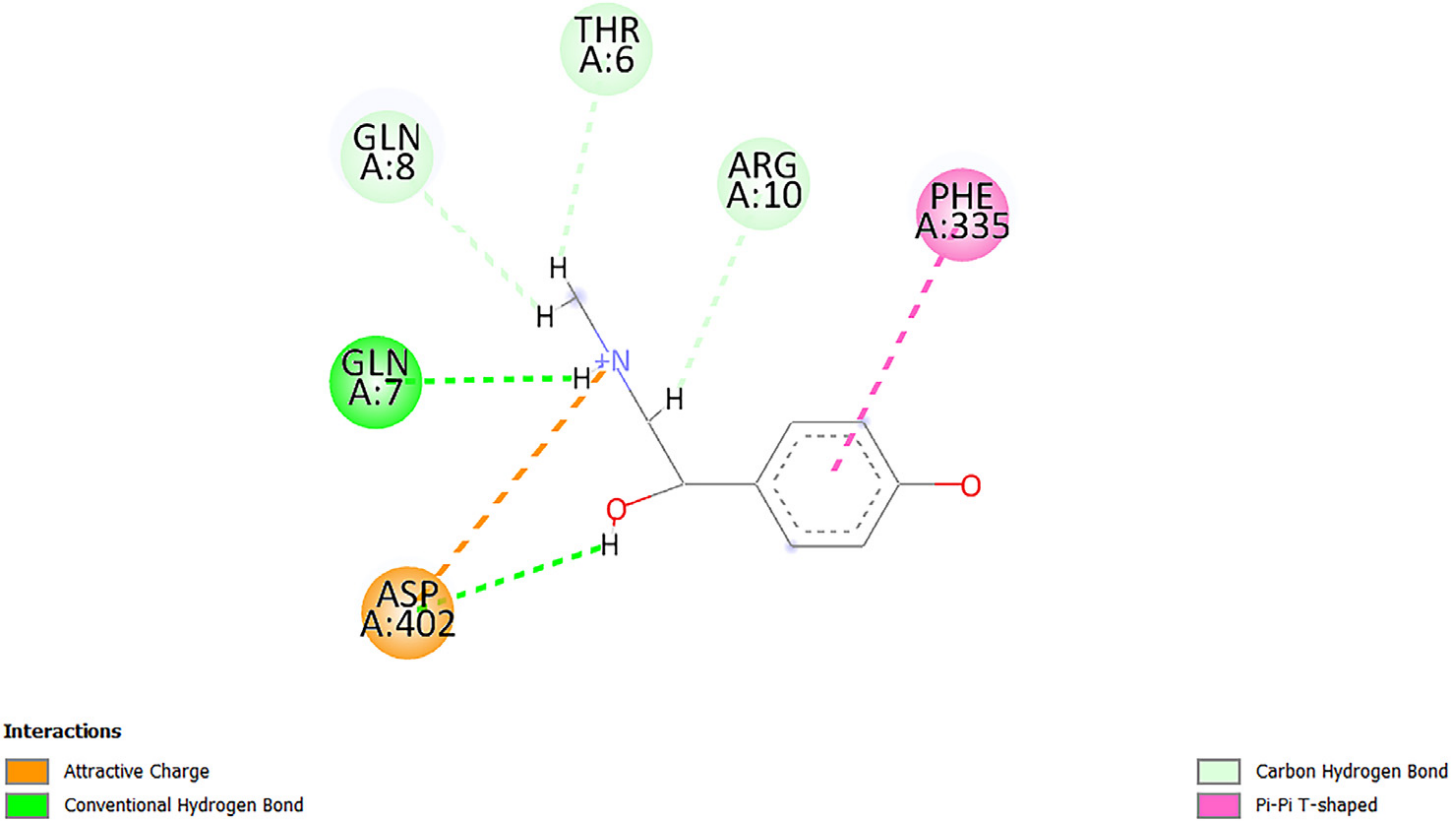
Two-dimensional interaction diagram of Synephrine with alpha-amylase (PDB ID: 2QMK). This figure highlights critical binding interactions and their potential role in modulating enzymatic function.

**Fig. 21:**
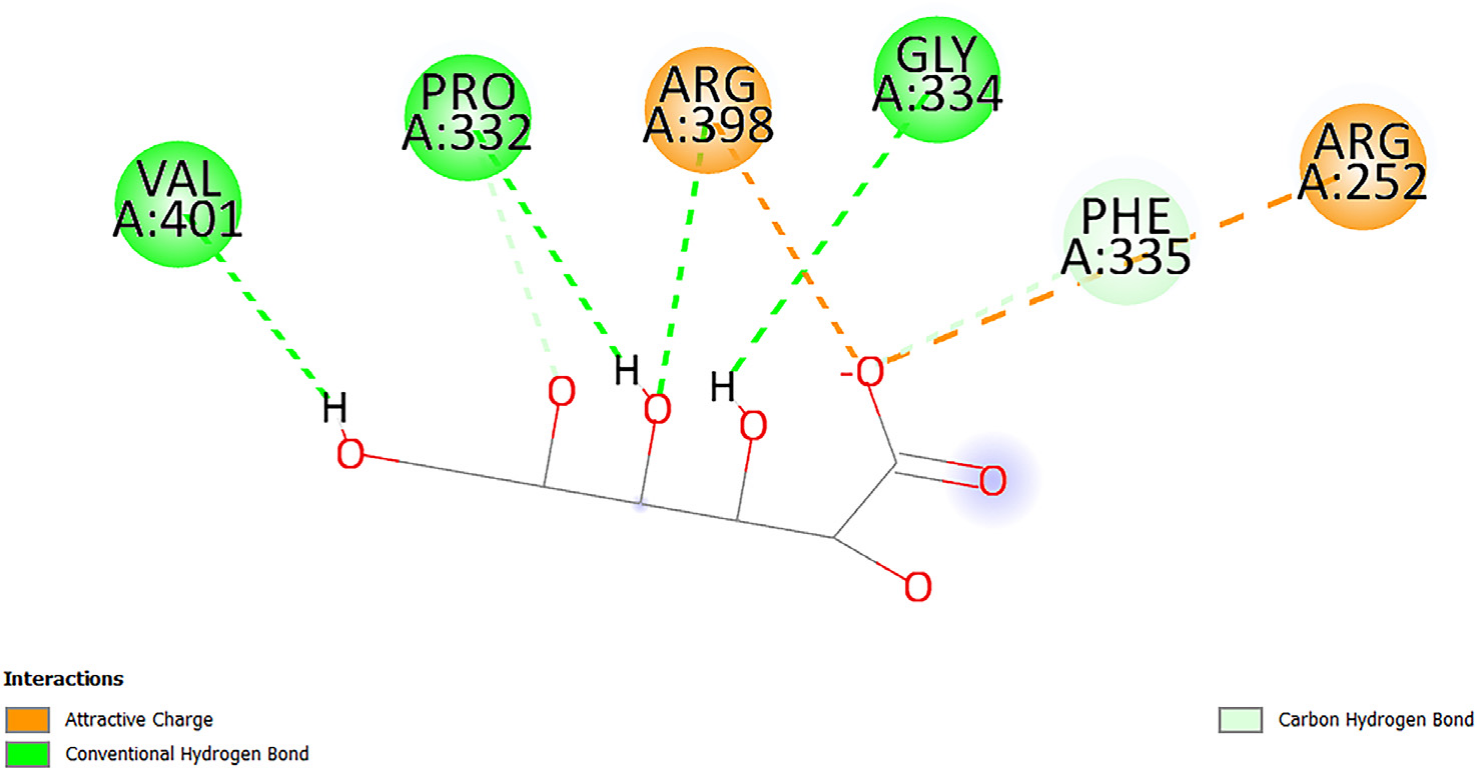
Two-dimensional interaction diagram of Mannoic acid with alpha-amylase (PDB ID: 2QMK), representation focus on active site residues interacting with hydrogen bond and attractive charge, emphasizing its role in modulating enzymatic activity.

**Fig. 22:**
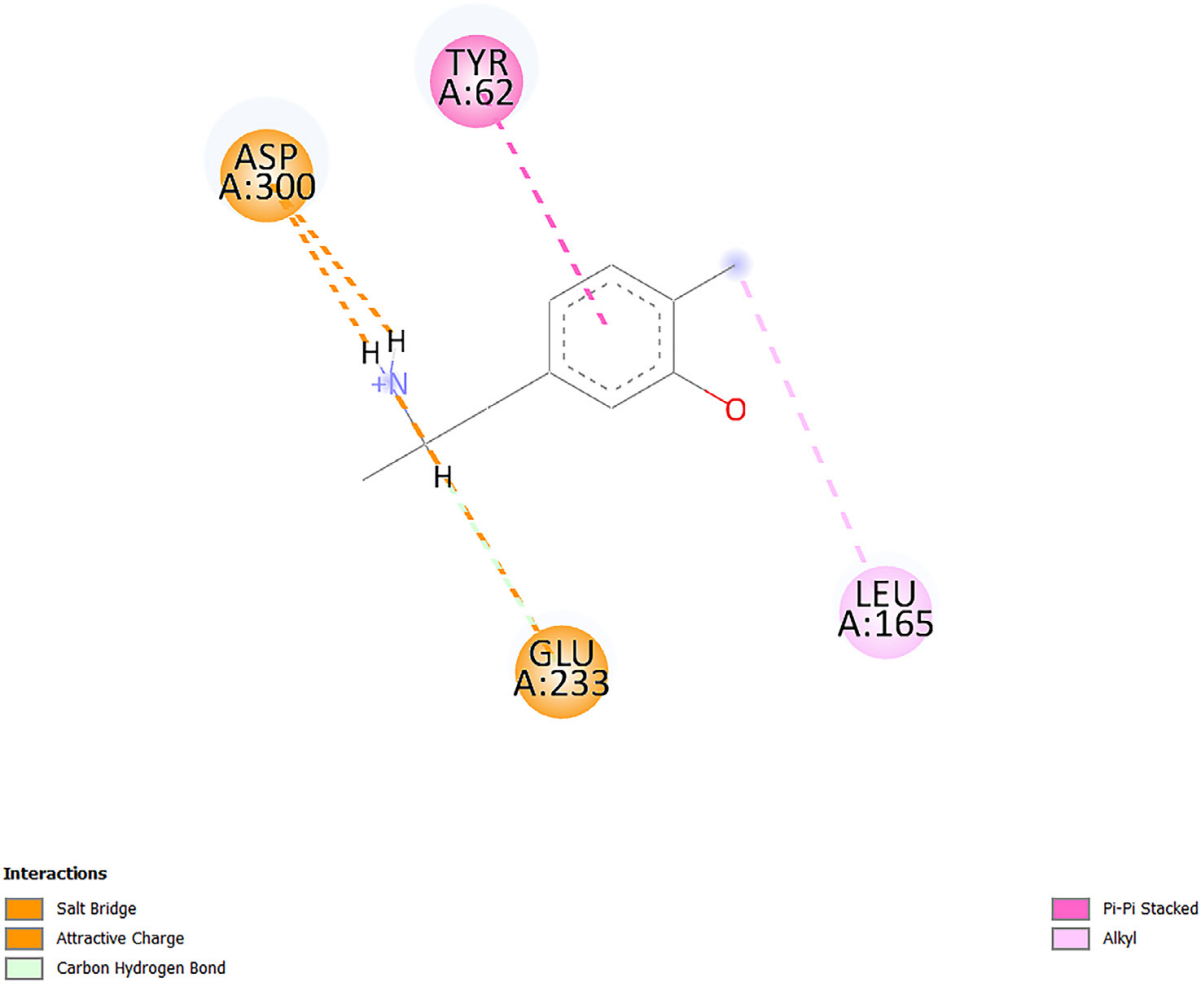
Two-dimensional interaction diagram of 5-(2-Aminopropyl)−2-methylphenol with alpha-amylase (PDB ID: 2QMK), illustrating the structural basis for the interaction and its potential effects on enzymatic activity.

**Fig. 23:**
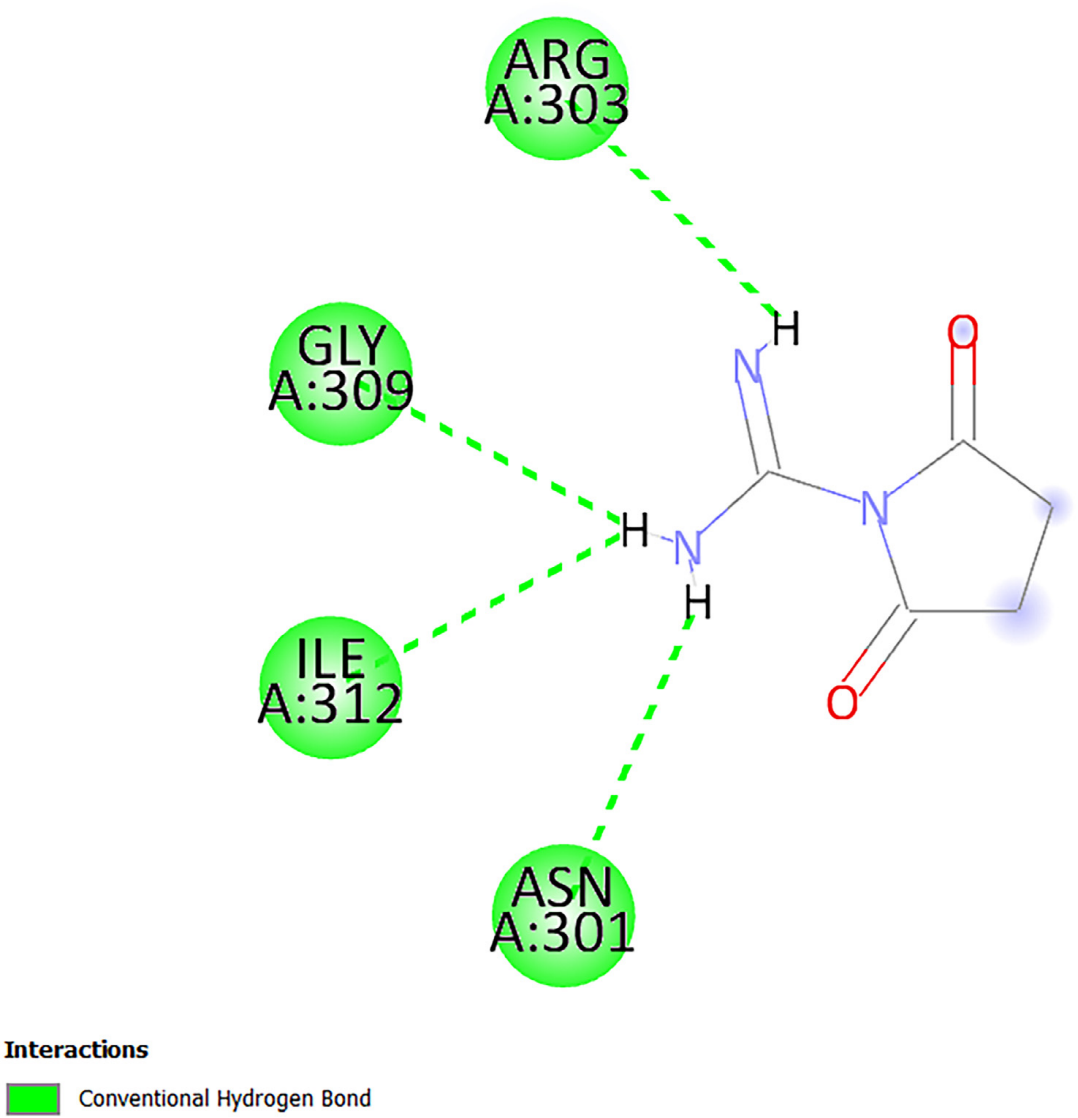
Two-dimensional interaction diagram of Guanidinosuccinimide with alpha-amylase (PDB ID: 2QMK). The figure illustrates the molecular interaction profile, emphasizing hydrogen bonding networks.

**Fig. 24:**
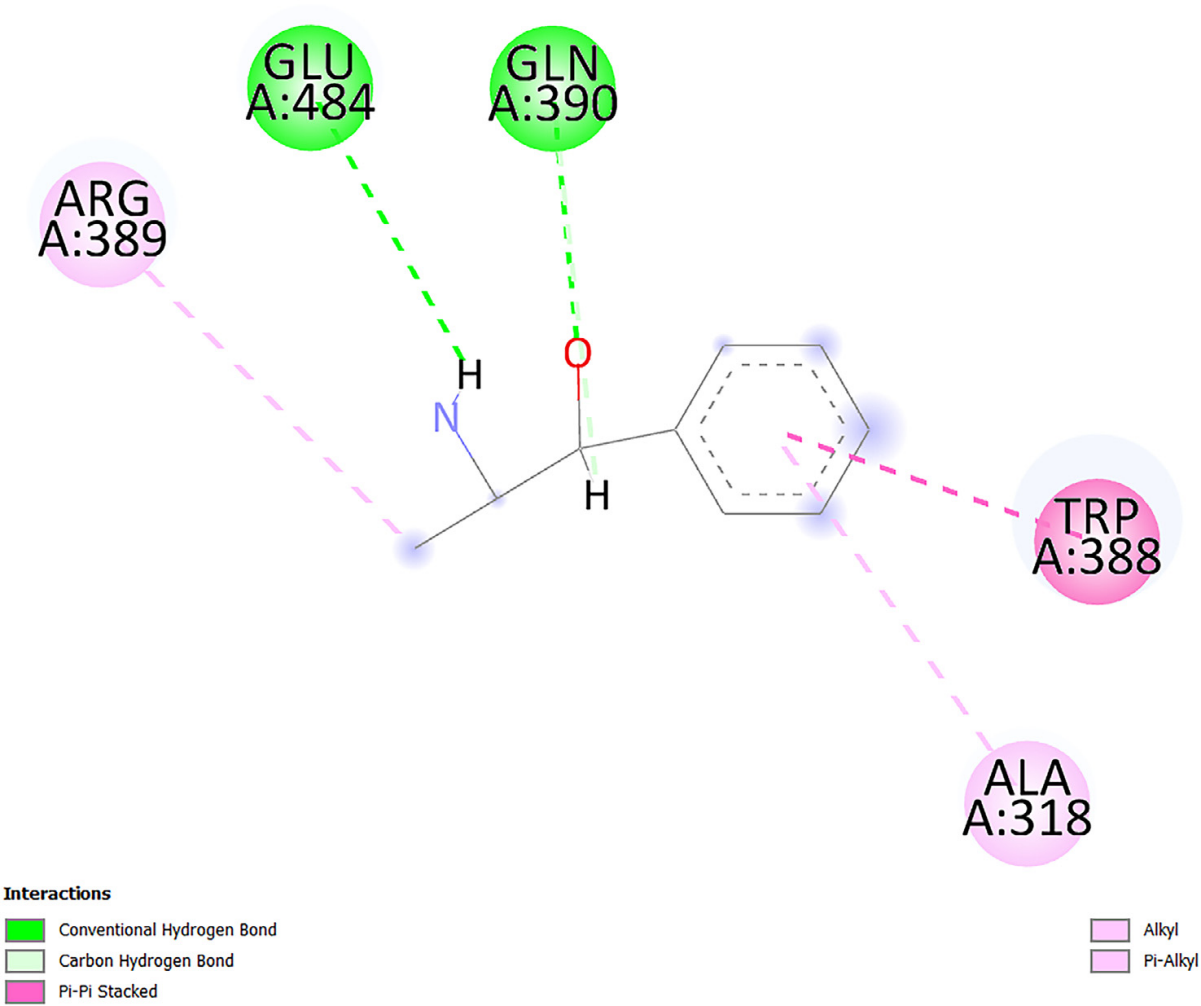
Two-dimensional interaction diagram of Cathine with alpha-amylase (PDB ID: 2QMK) illustrating key binding interactions, including hydrogen bonds, hydrophobic contacts, and ionic interactions, and their contribution to the binding energy and specificity of Cathine.

**Fig. 25:**
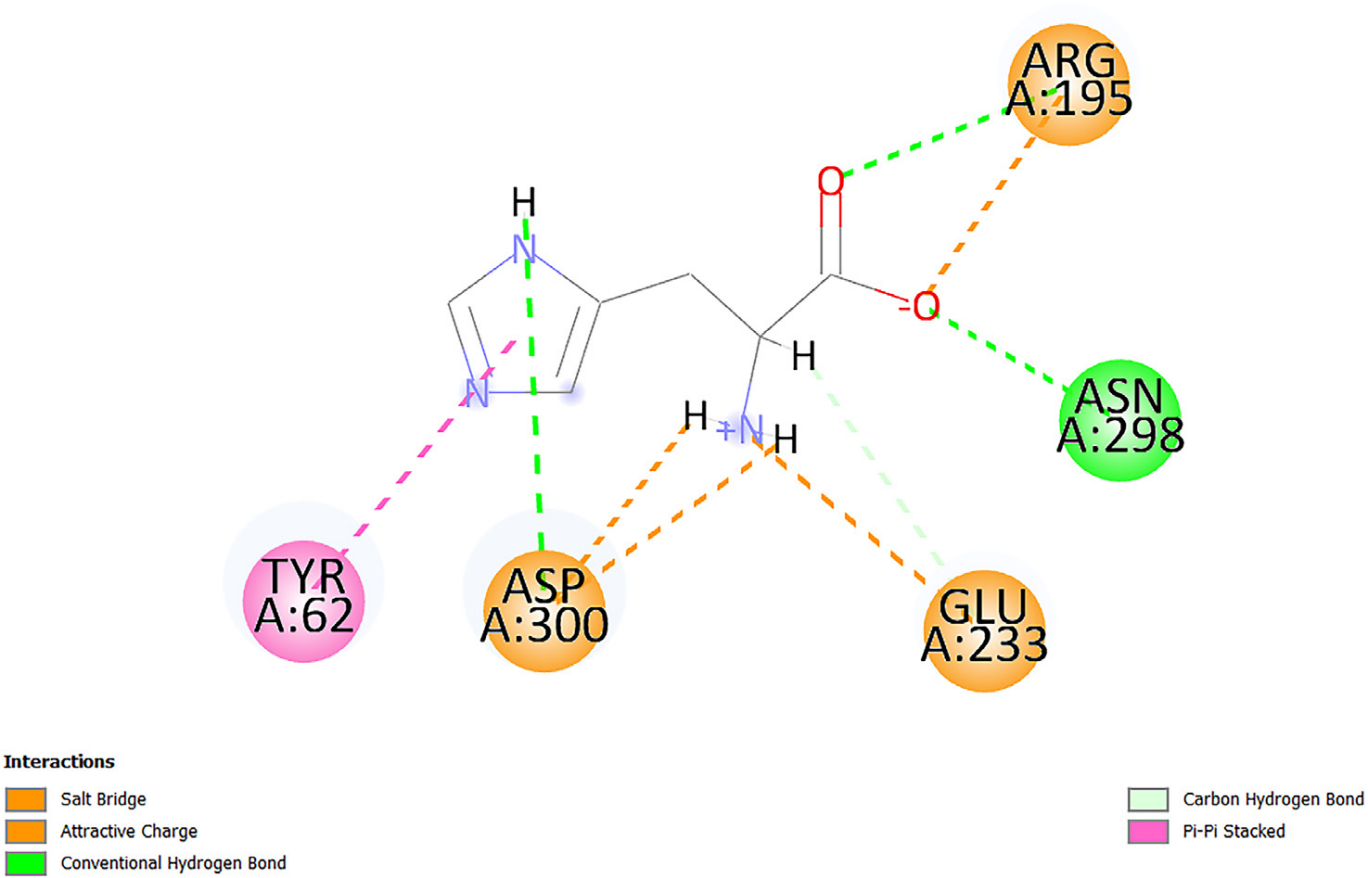
Two-dimensional interaction diagram of L-histidine with alpha-amylase (PDB ID: 2QMK). This figure highlights the interaction network by hydrogen bonds, alkyl contacts, and electrostatic interactions within the active site, revealing how L-histidine aligns with the catalytic residues to facilitate enzymatic hydrolysis.

**Fig. 26:**
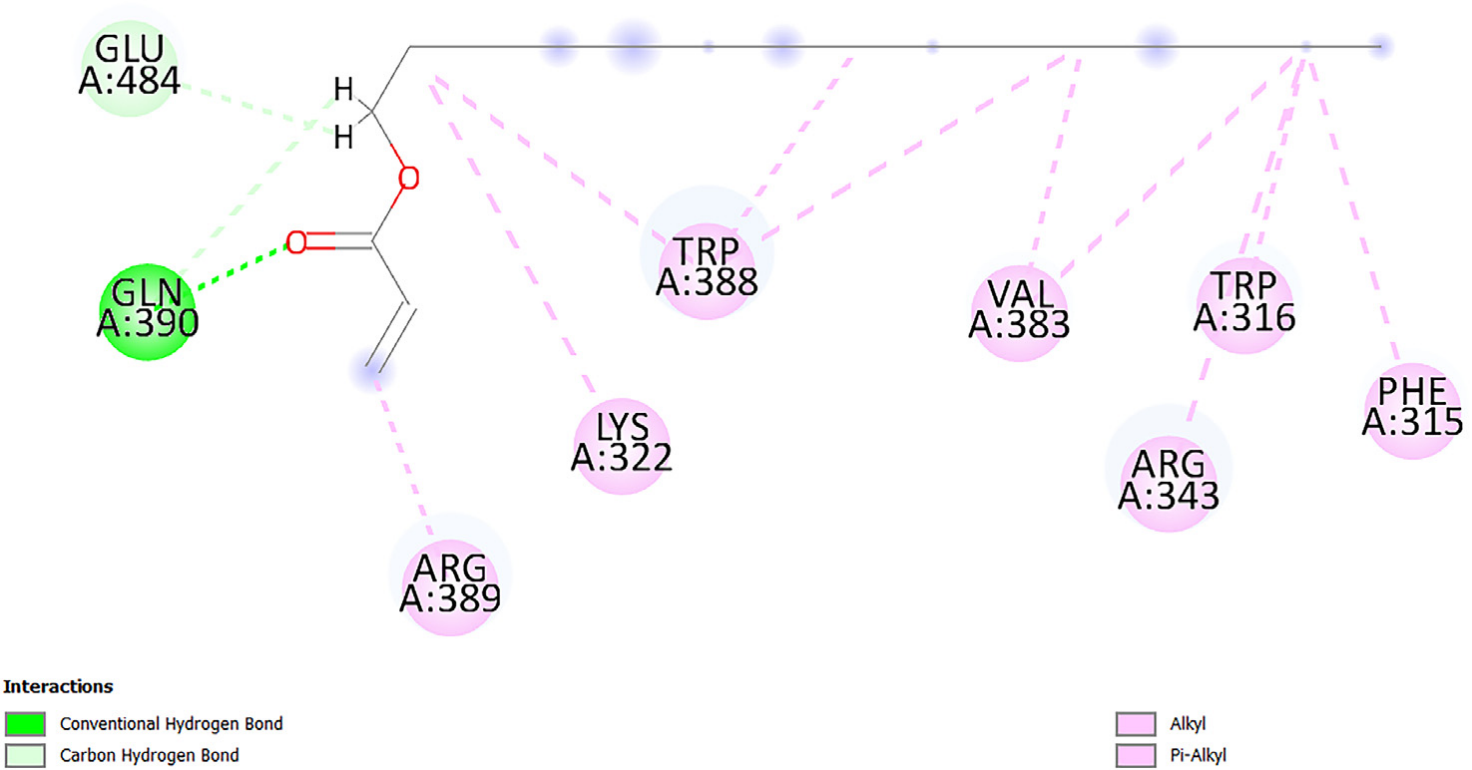
Two-dimensional interaction diagram of 2-Propenoic acid, n-pentadecyl ester with alpha-amylase (PDB ID: 2QMK) showing detailed interaction features, including hydrogen bonding and ionic intereactions.

**Fig. 27:**
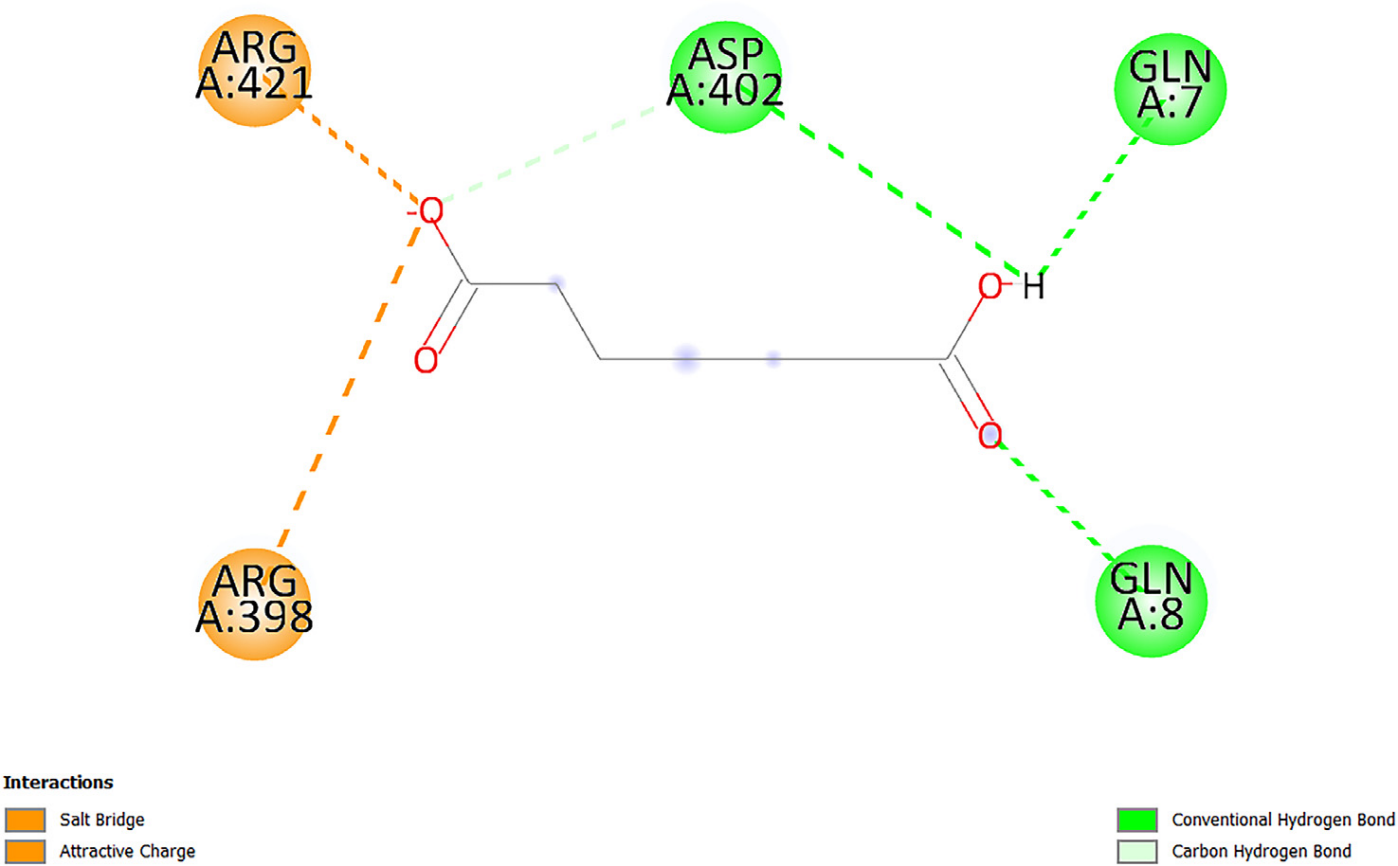
2D interactions of Heptanedioic acid with alpha-amylase (PDB ID: 2QMK). The binding interactions depicted highlight the molecular features that may influence enzymatic activity.

**Fig. 28:**
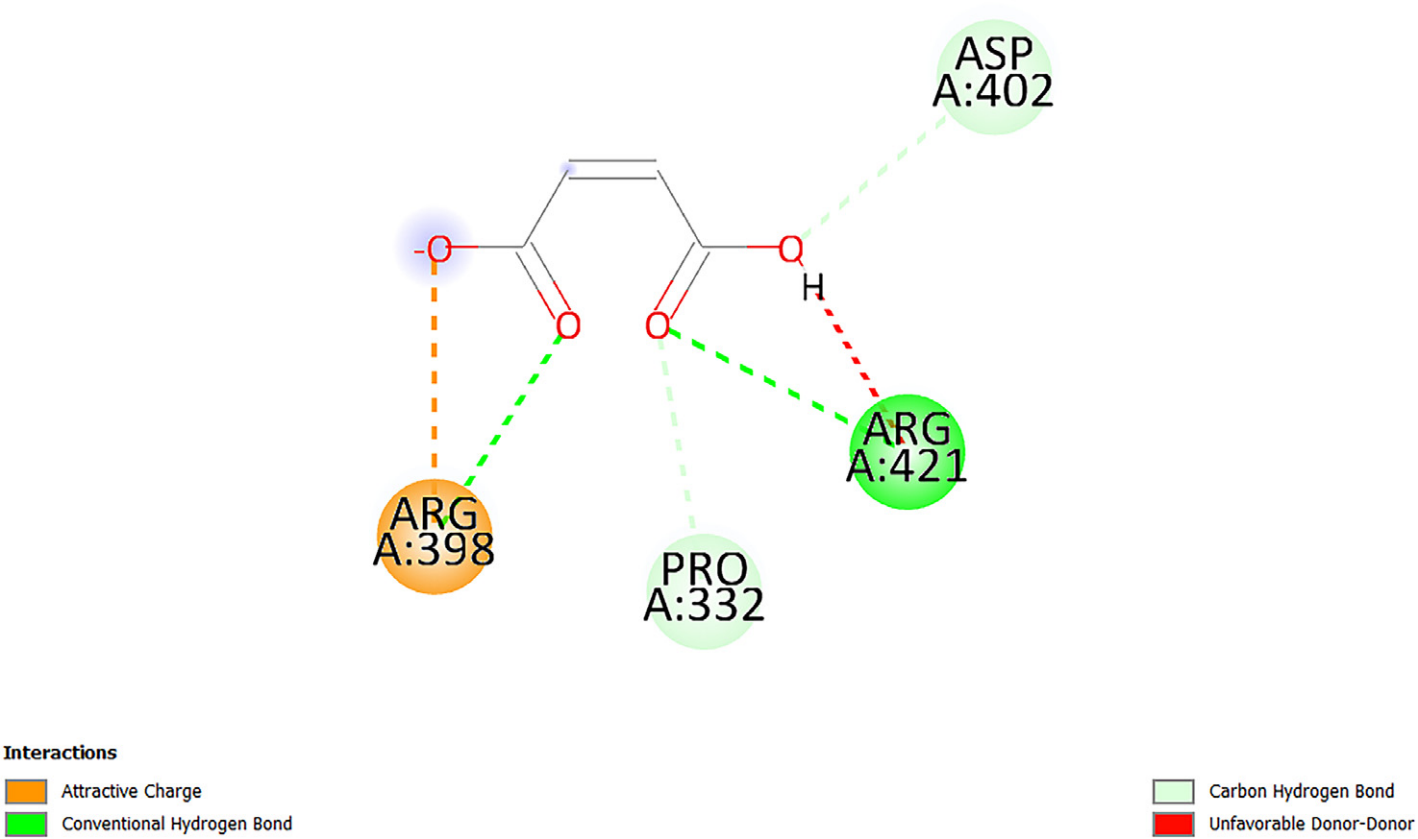
Two-dimensional interaction diagram of 2-Butenedioic acid with alpha-amylase (PDB ID: 2QMK).

**Fig. 29:**
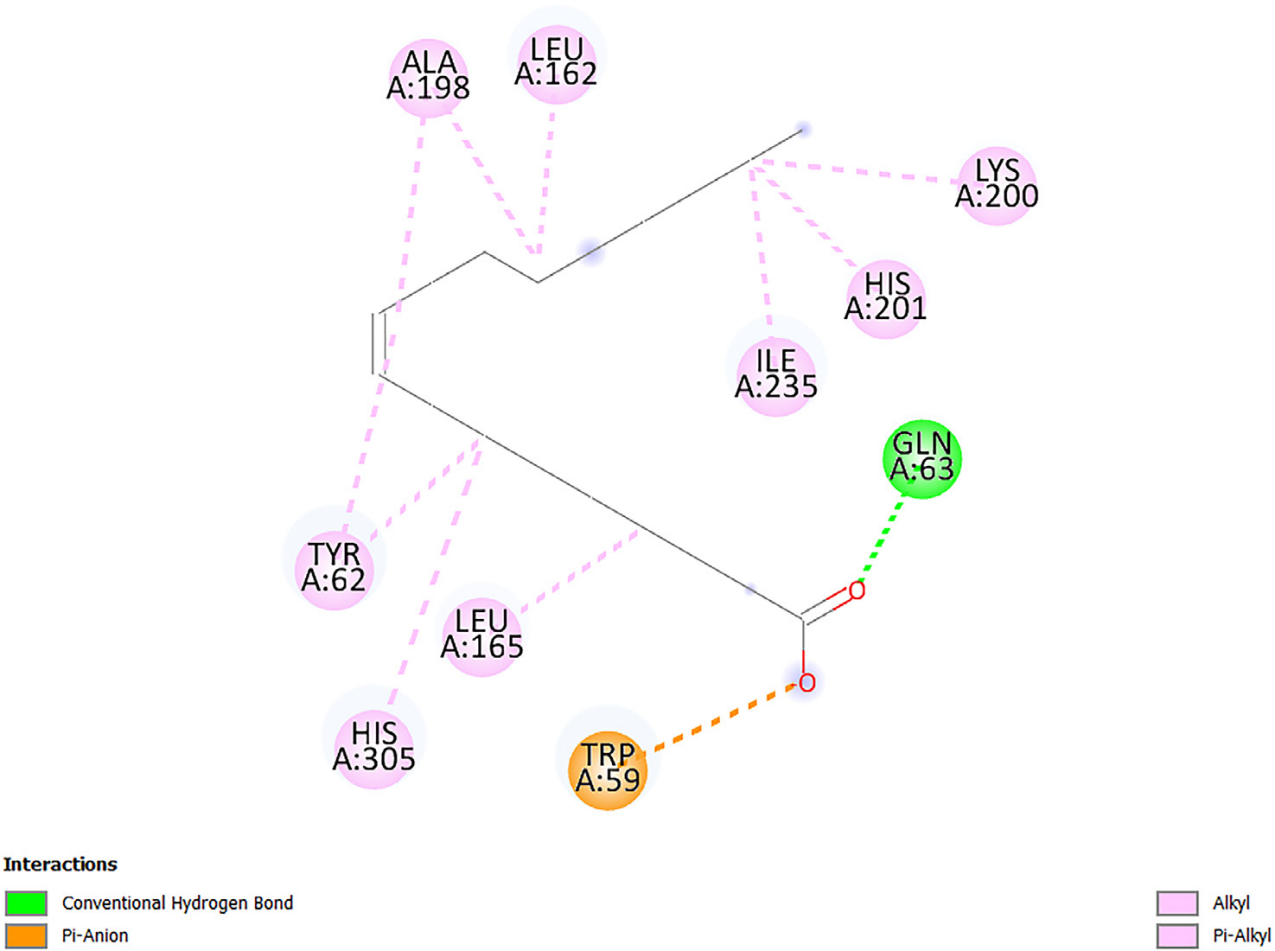
Two-dimensional interaction diagram of 10-Octadecenoic acid with alpha-amylase (PDB ID: 2QMK).

**Fig. 30:**
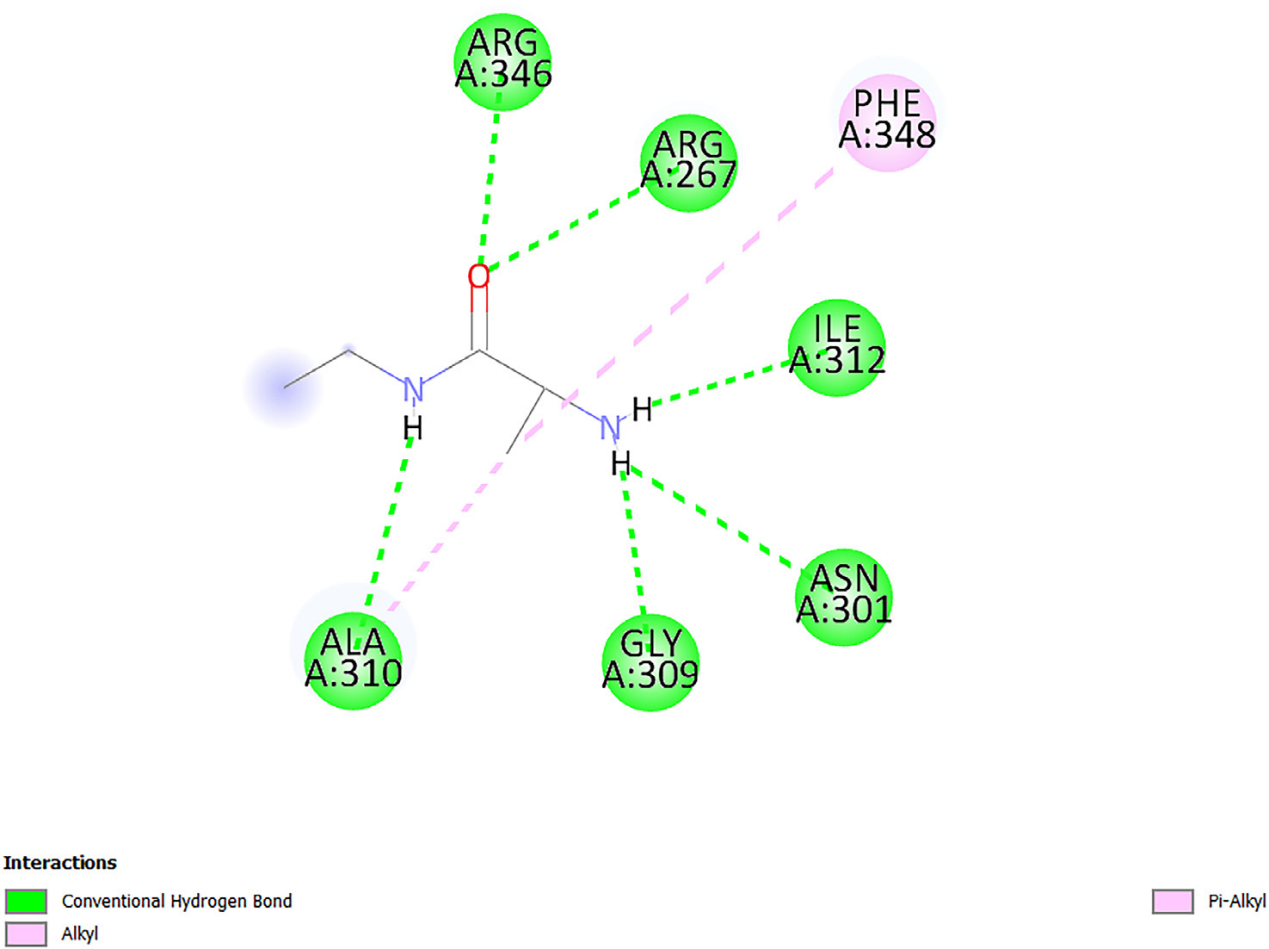
Two-dimensional interaction diagram of L- Alanine ethylamide, (S) with alpha-amylase (PDB ID: 2QMK).

**Fig. 31:**
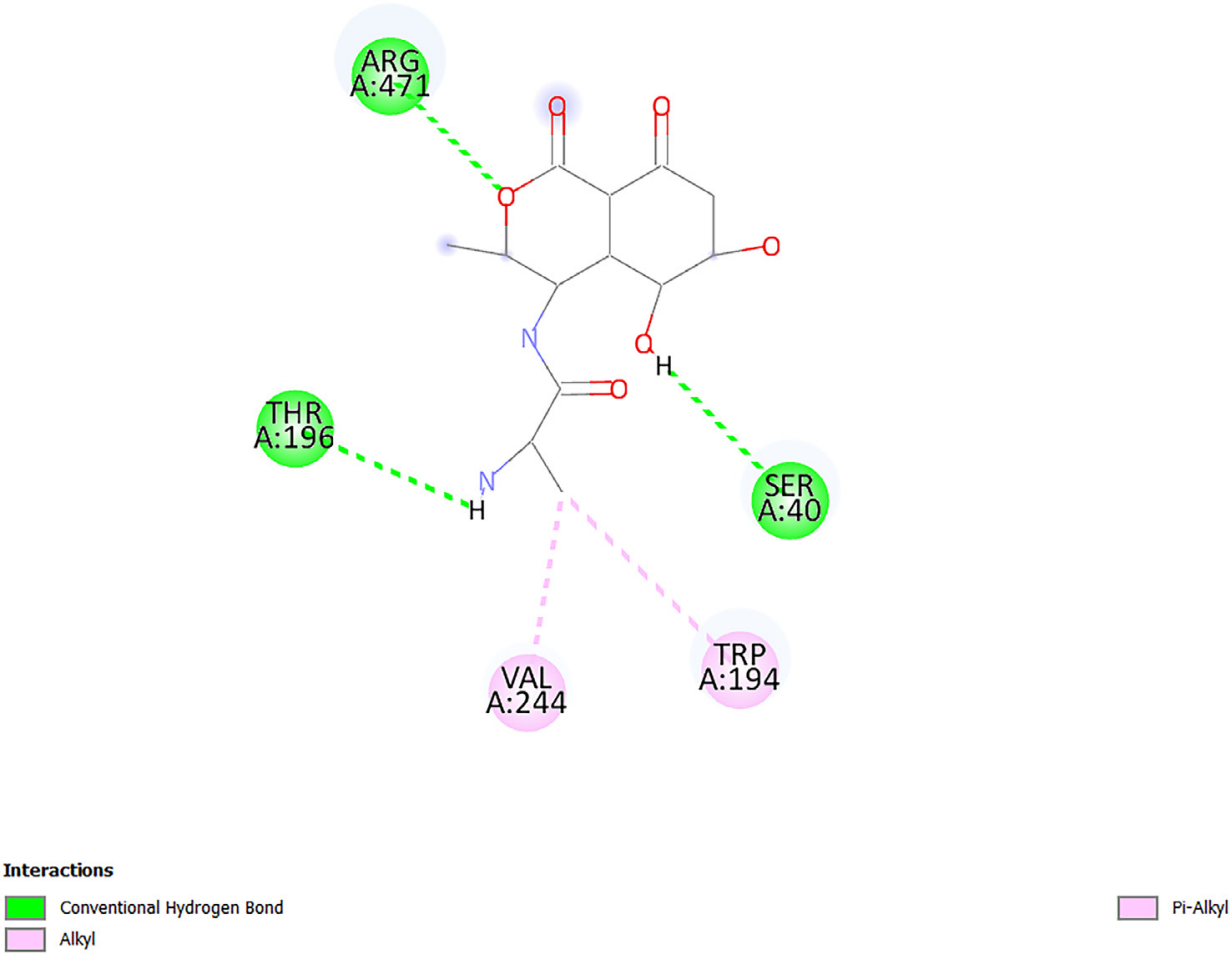
Two-dimensional interaction diagram of Actinobolin with human maltase gluco‑amylase (PDB ID: 2QMJ).

**Fig. 32:**
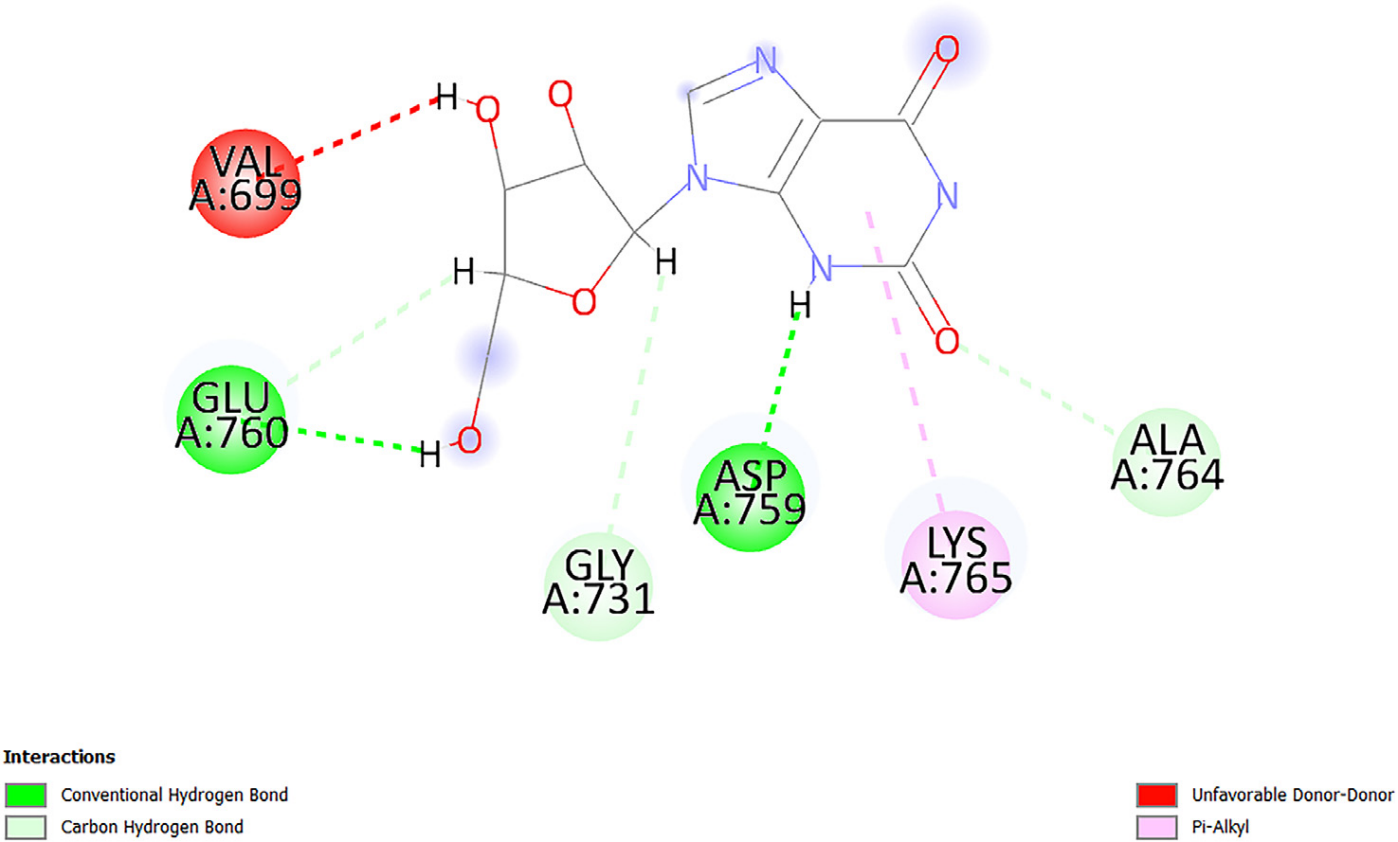
Two-dimensional interaction diagram of xanthosine with human maltase gluco‑amylase (PDB ID: 2QMJ).

**Fig. 33:**
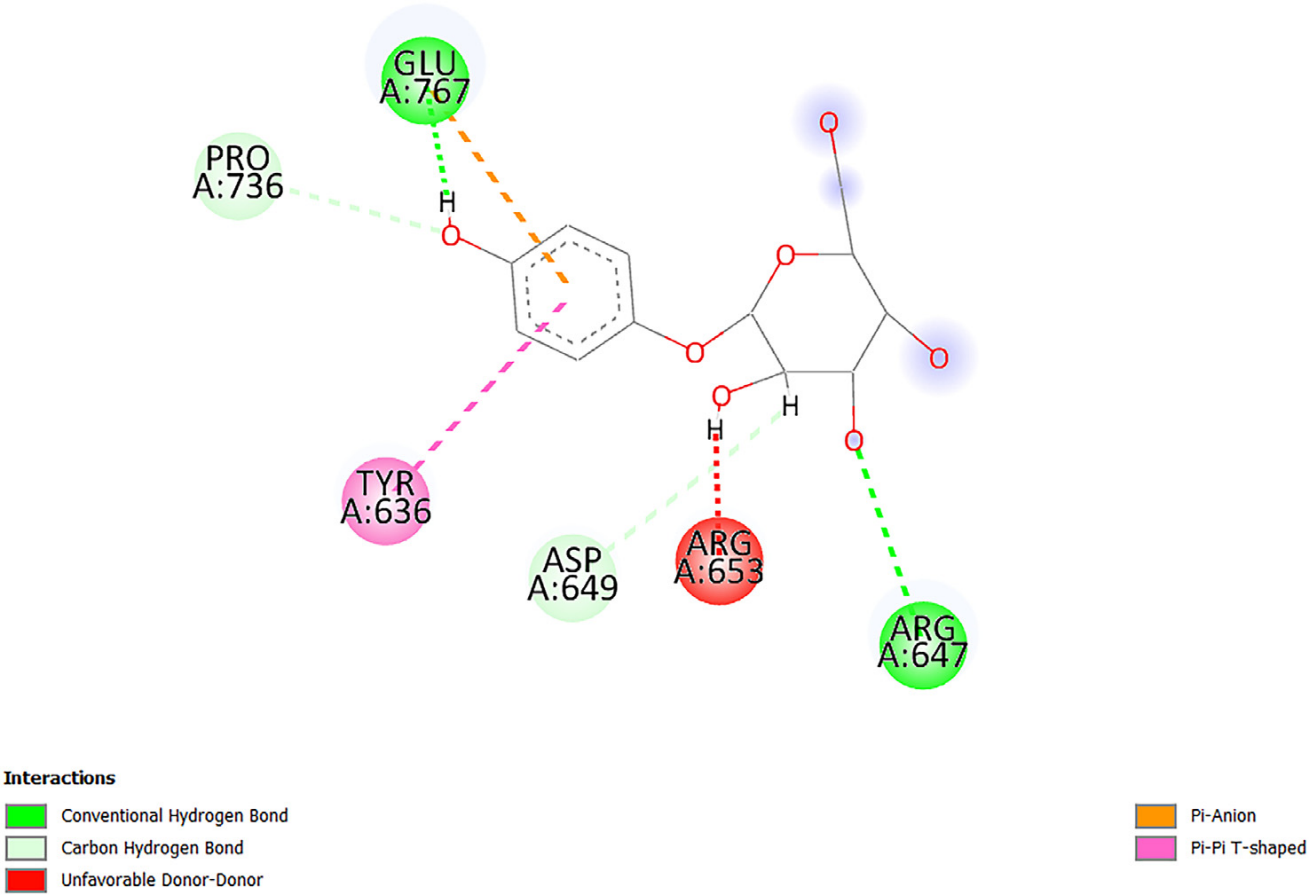
Two-dimensional interaction diagram of Arbutin with human maltase gluco‑amylase (PDB ID: 2QMJ).

**Fig. 34:**
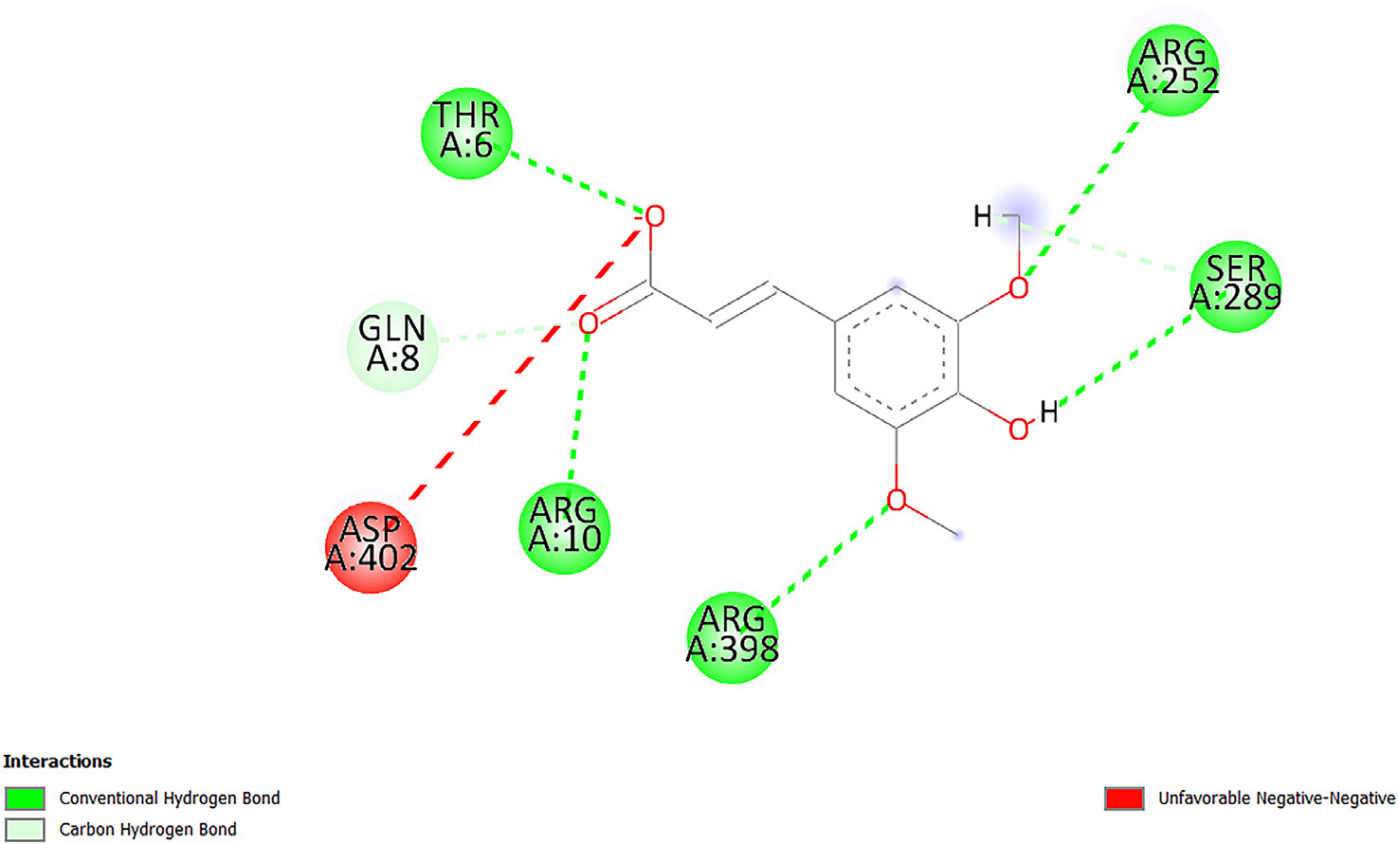
Two-dimensional interaction diagram of Sinapic acid with human maltase gluco‑amylase (PDB ID: 2QMJ).

**Fig. 35:**
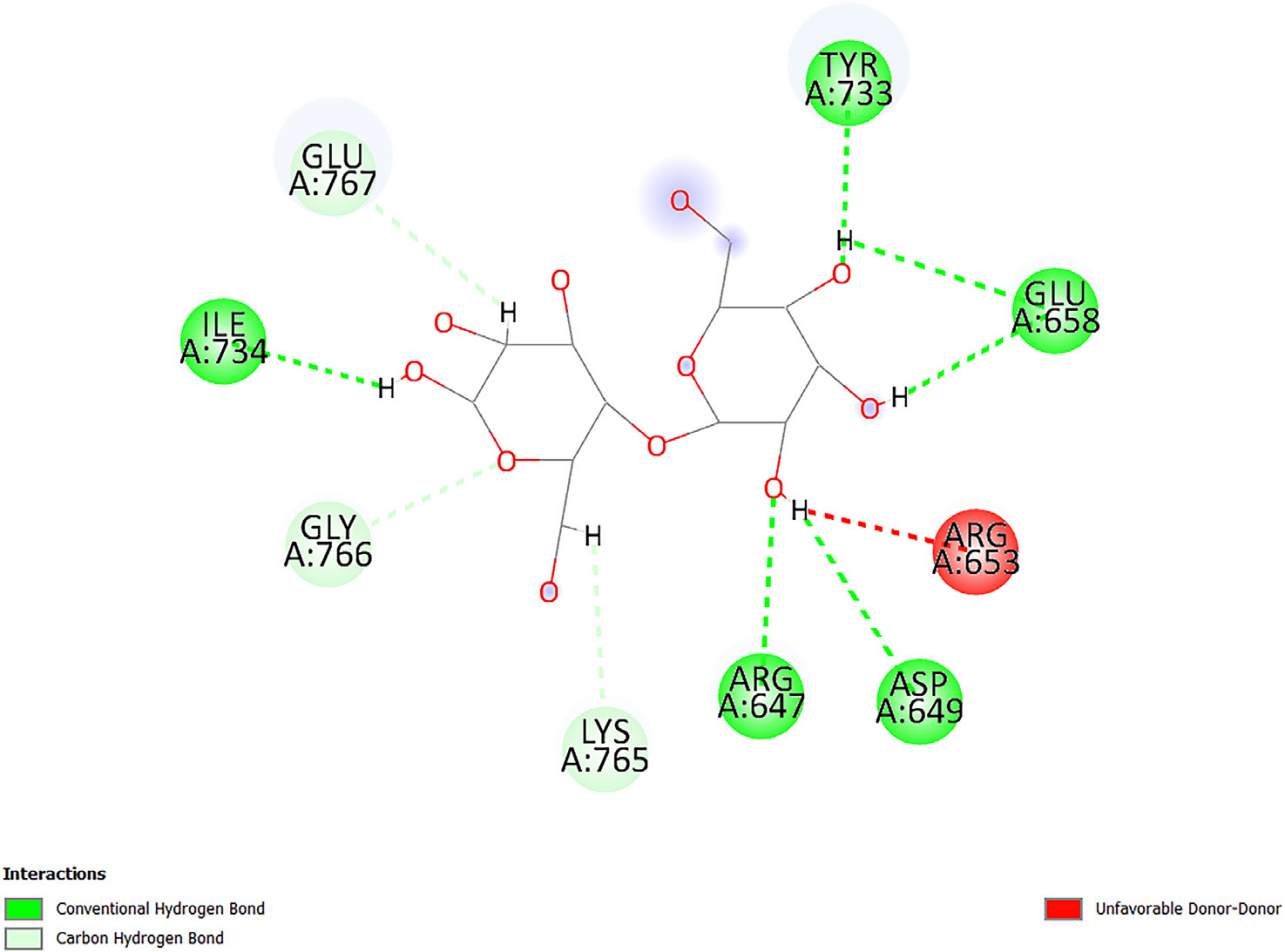
Two-dimensional interaction diagram of Maltose with human maltase gluco‑amylase (PDB ID: 2QMJ).

**Fig. 36:**
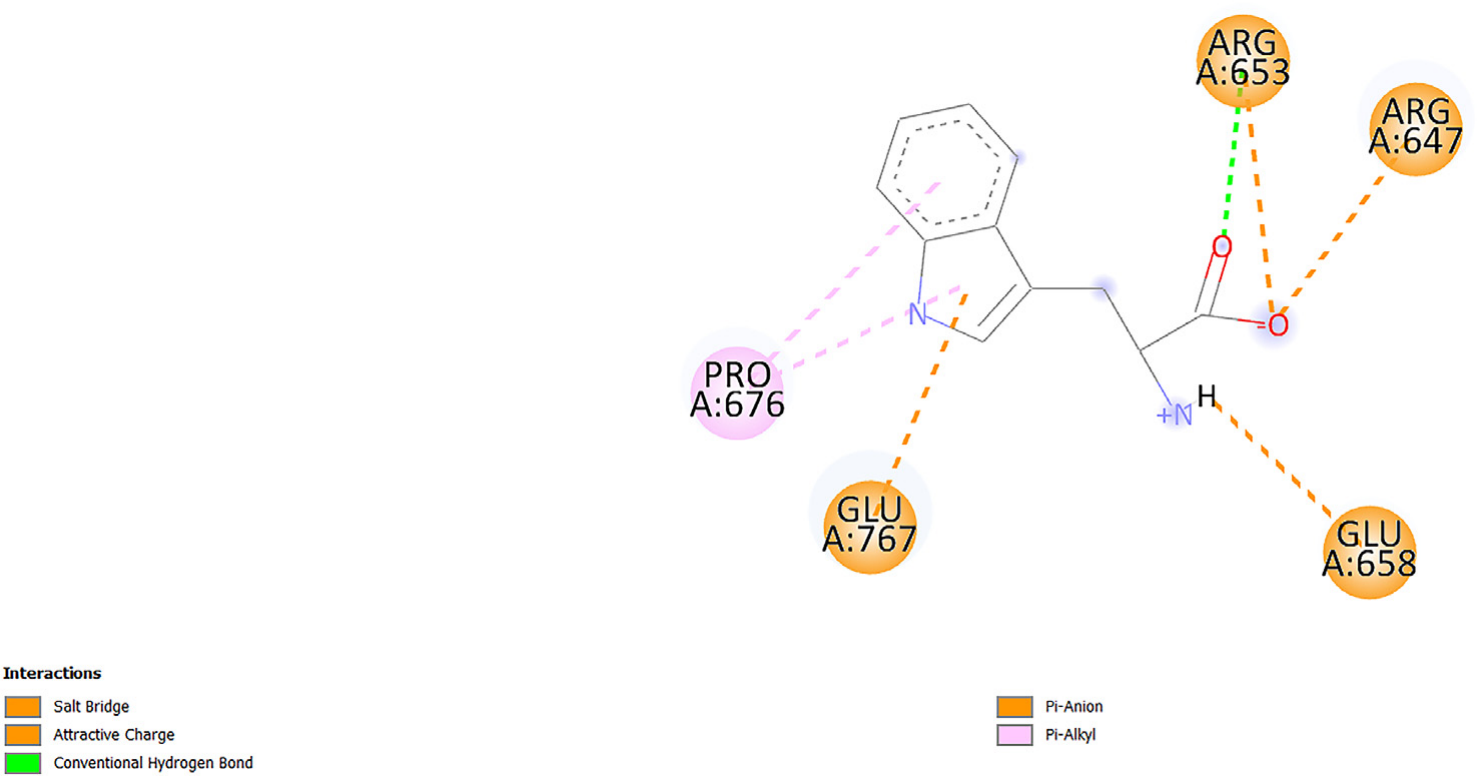
Two-dimensional interaction diagram of L-Tryptophan with human maltase gluco‑amylase (PDB ID: 2QMJ).

**Fig. 37:**
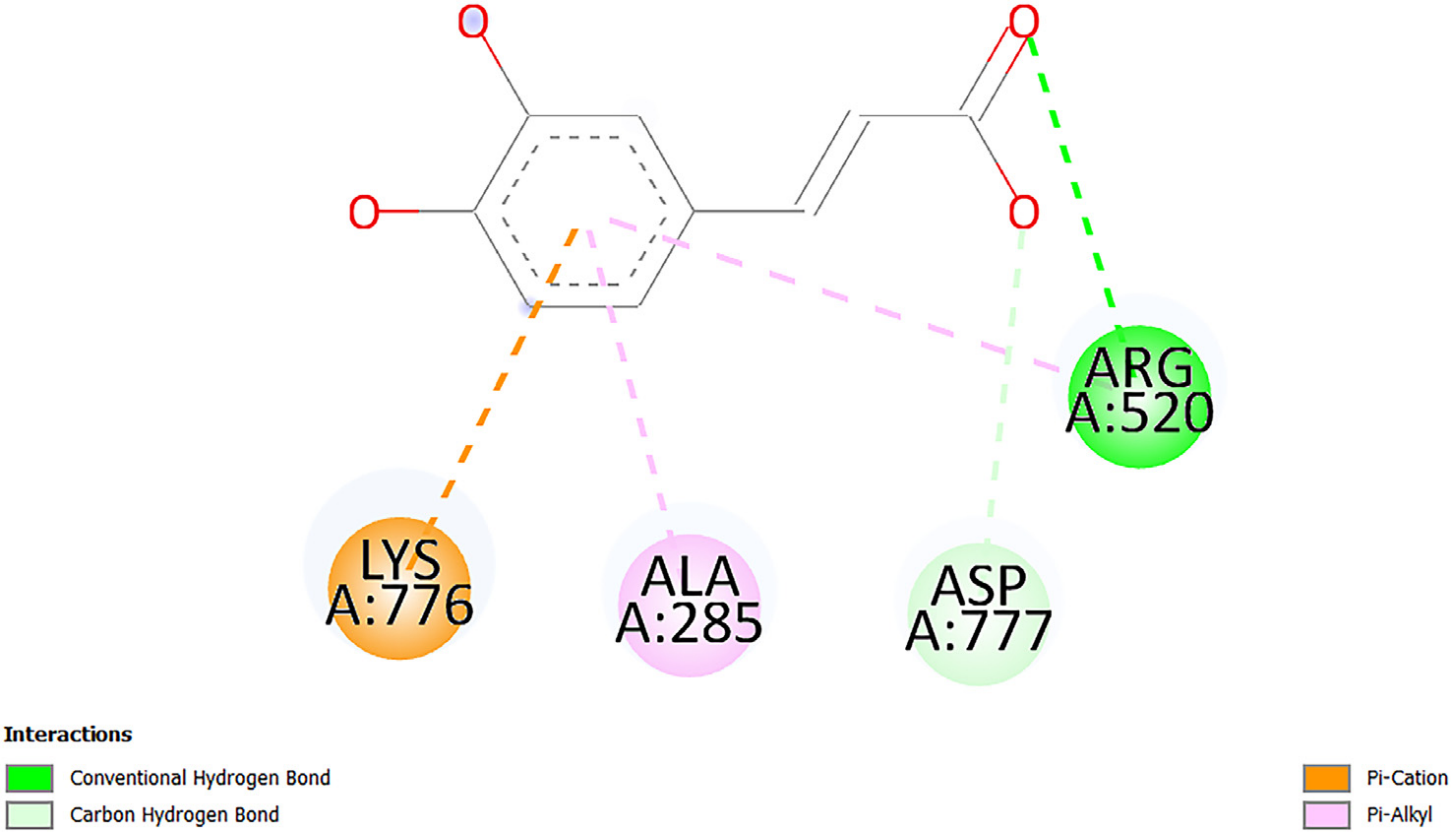
Two-dimensional interaction diagram of Caffeic acid with human maltase gluco‑amylase (PDB ID: 2QMJ).

**Fig. 38:**
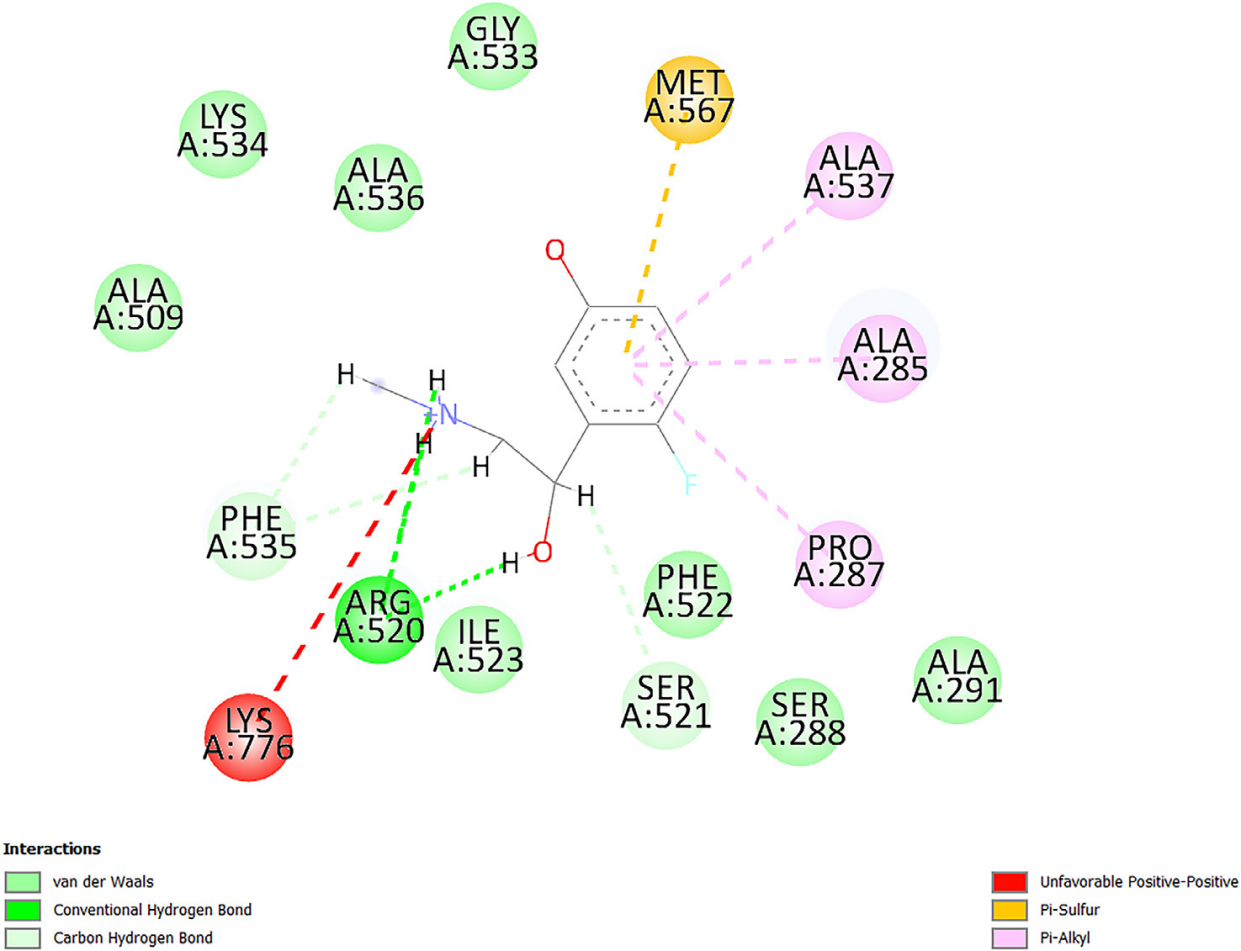
Two-dimensional interaction diagram of Pyrido[3,4-d]imidazole, 1. 6-dicarboxylic acid with human maltase gluco‑amylase (PDB ID: 2QMJ).

**Fig. 39:**
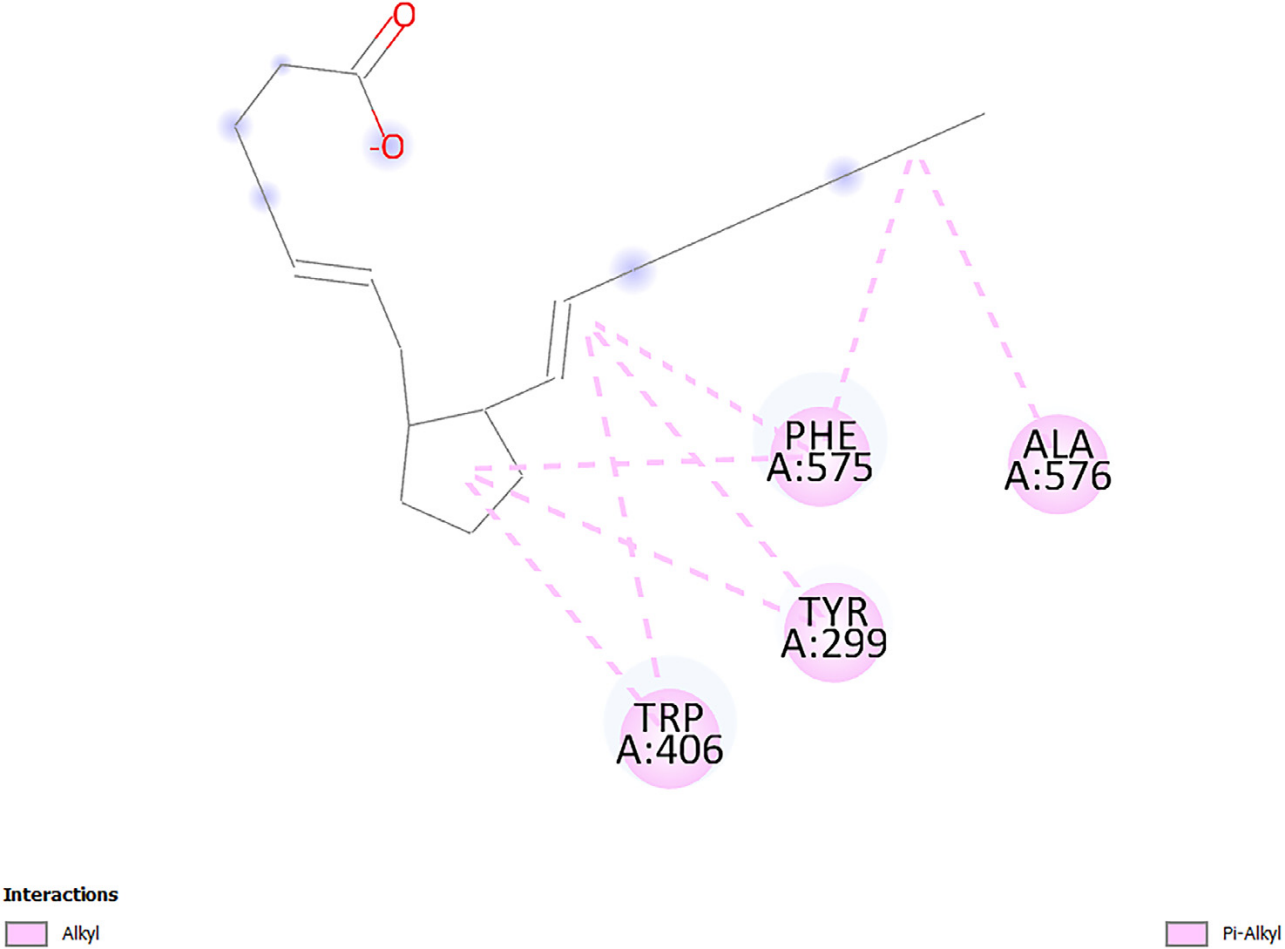
Two-dimensional interaction diagram of Prosta 5,13‑dien-1-oic acid with human maltase gluco‑amylase (PDB ID: 2QMJ). This figure emphasizes the role of hydrogen bonds and hydrophobic contacts in stabilizing the ligand within the binding pocket.

**Fig. 40:**
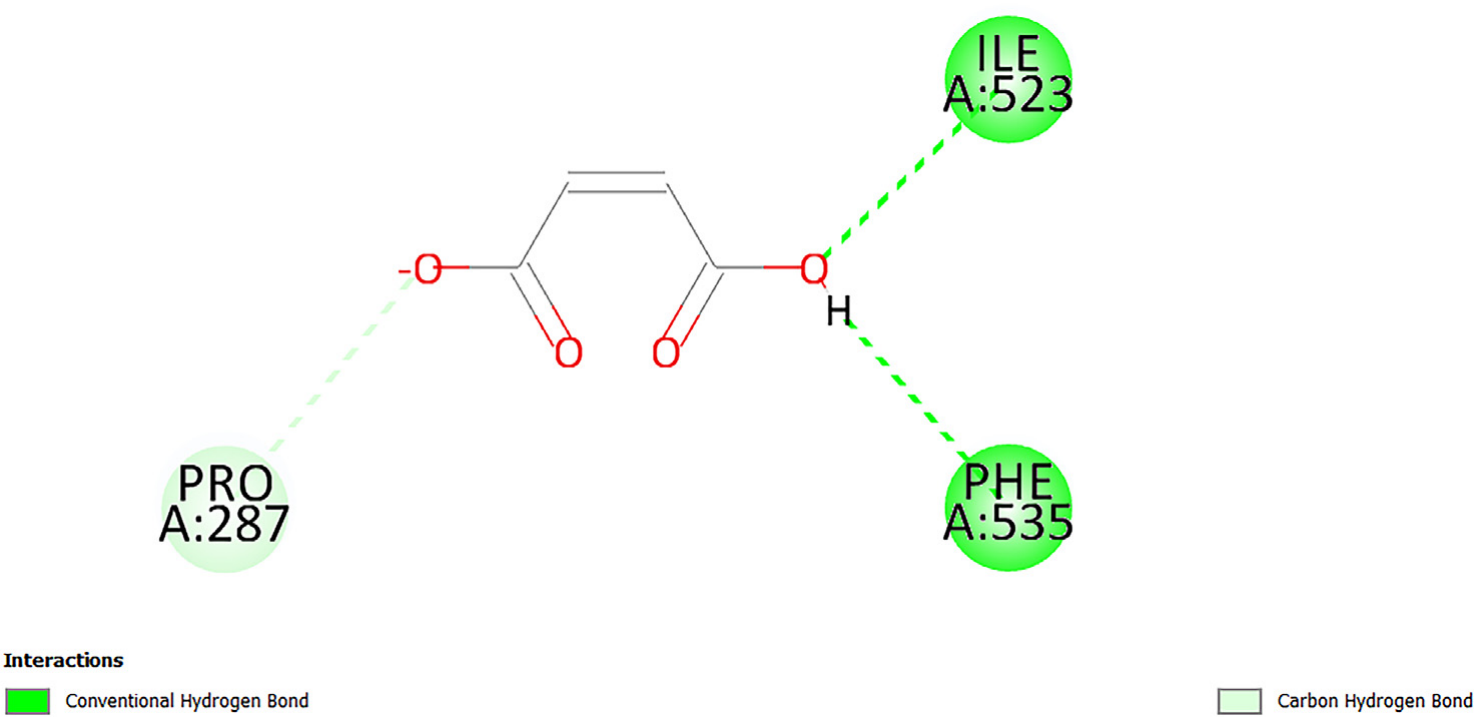
Two-dimensional interaction diagram of L-Tyrosine with human maltase gluco‑amylase (PDB ID: 2QMJ) focusing on the interaction dynamics and their implications for enzyme-ligand affinity.

**Fig. 41:**
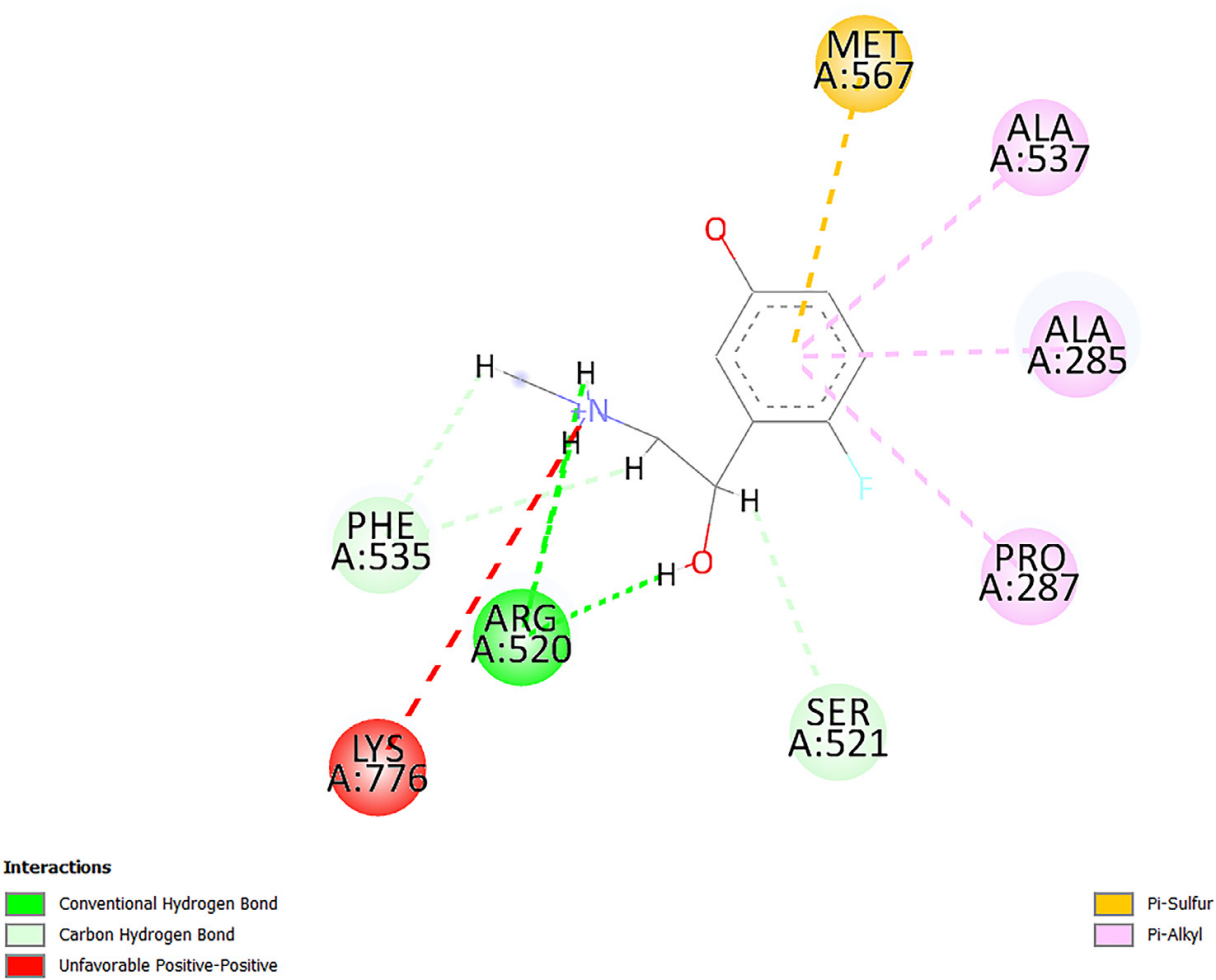
Two-dimensional interaction diagram of 4-Fluoro-3-[1‑hydroxy-2-(methylamino)ethyl] phenol with human maltase gluco‑amylase (PDB ID: 2QMJ), illustrating the molecular binding landscape and key residues involved in the interaction.

**Fig. 42:**
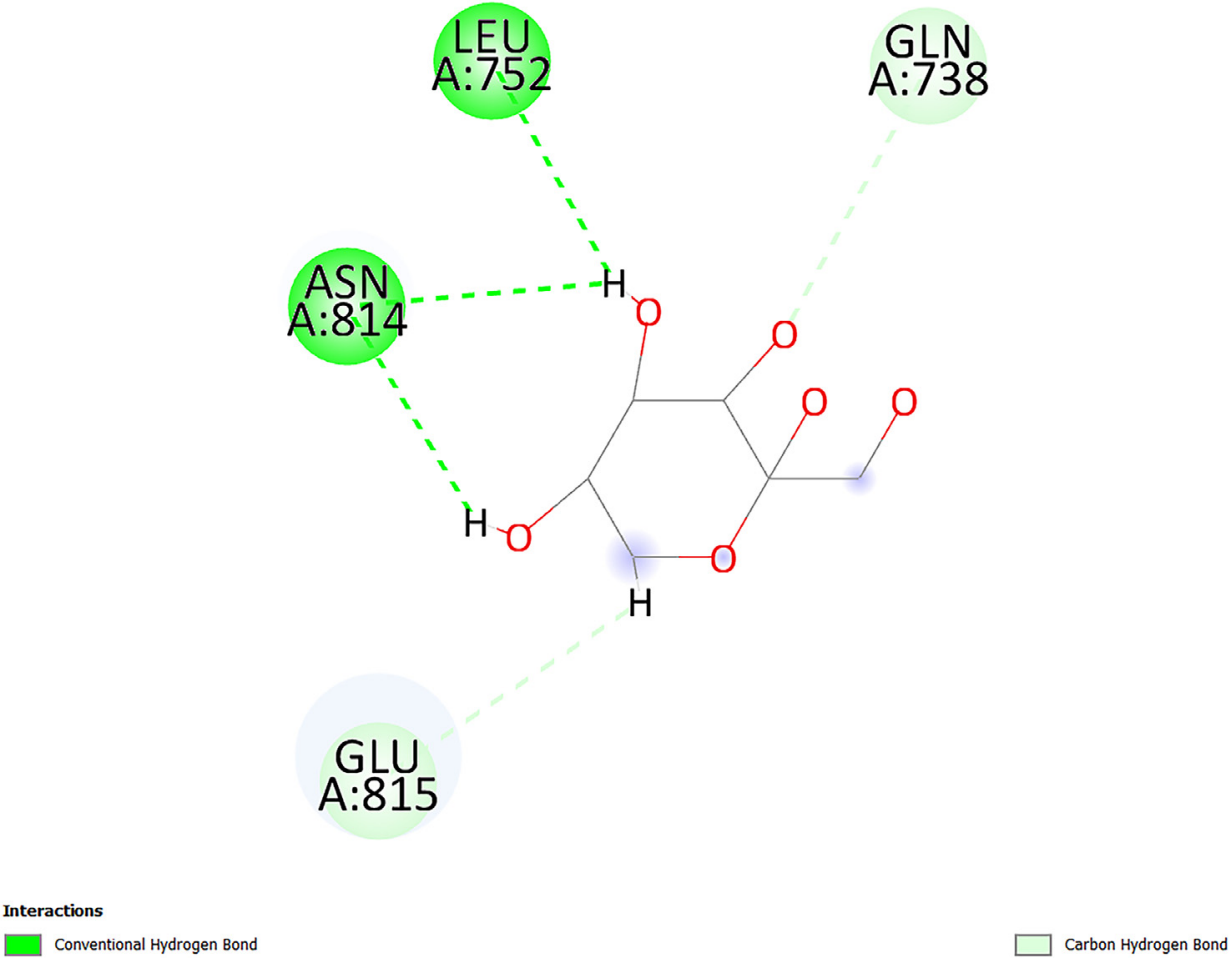
Two-dimensional interaction diagram of D-Fructopyranose with human maltase gluco‑amylase (PDB ID: 2QMJ).

**Fig. 43:**
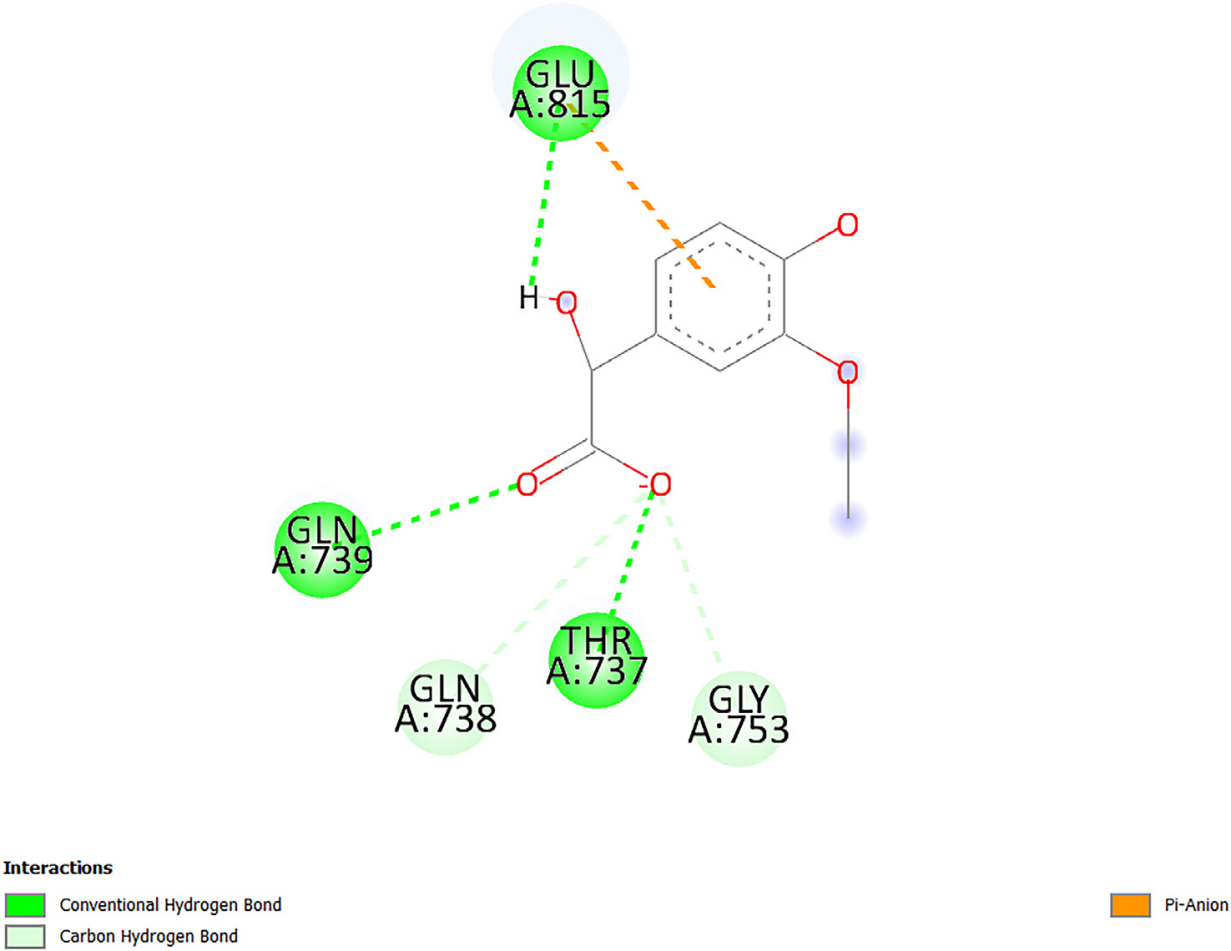
Two-dimensional interaction diagram of 3-Ethoxy-4-hydroxymandelic acid with human maltase gluco‑amylase (PDB ID: 2QMJ).

**Fig. 44:**
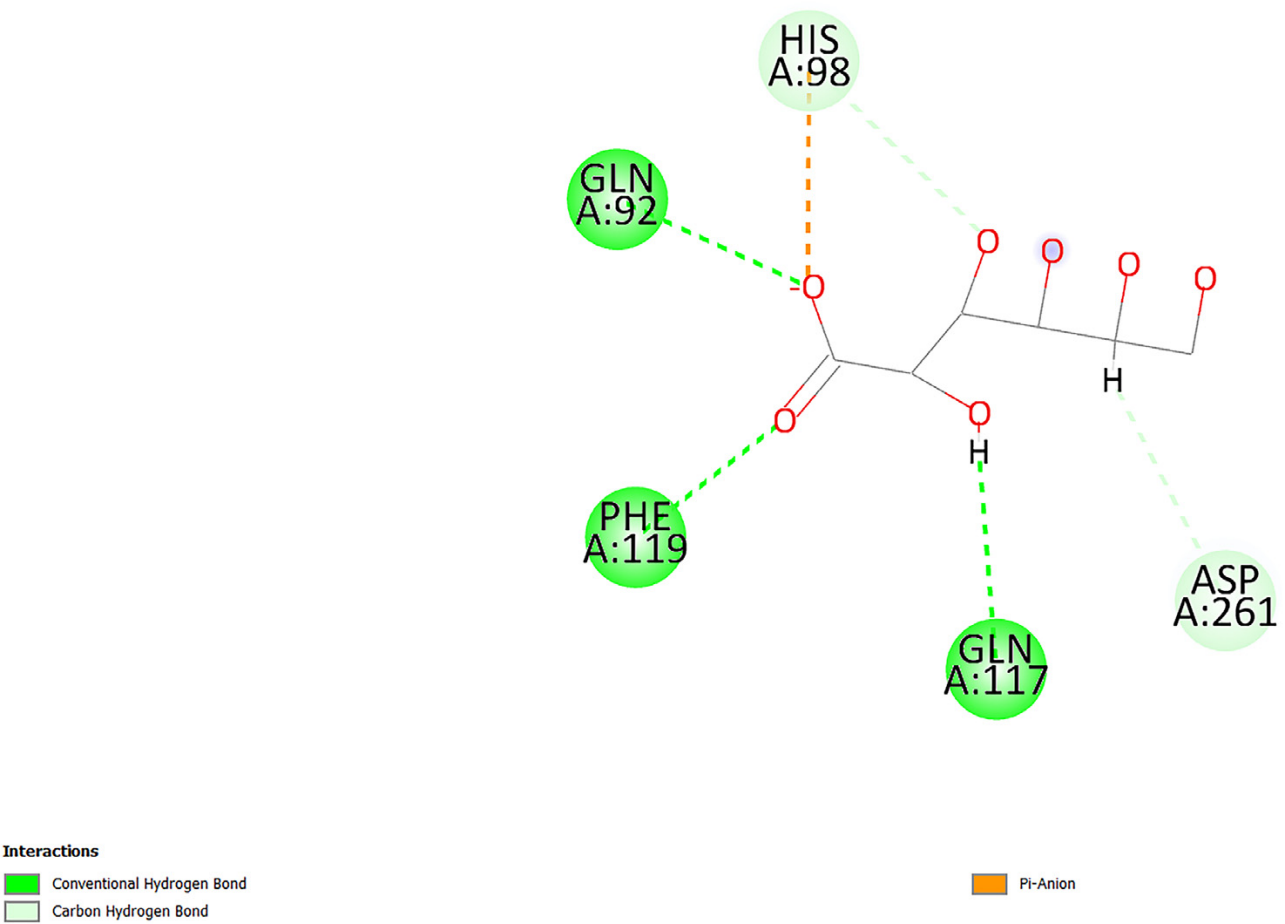
Two-dimensional interaction diagram of Gluconic acid with human maltase gluco‑amylase (PDB ID: 2QMJ).

**Fig. 45:**
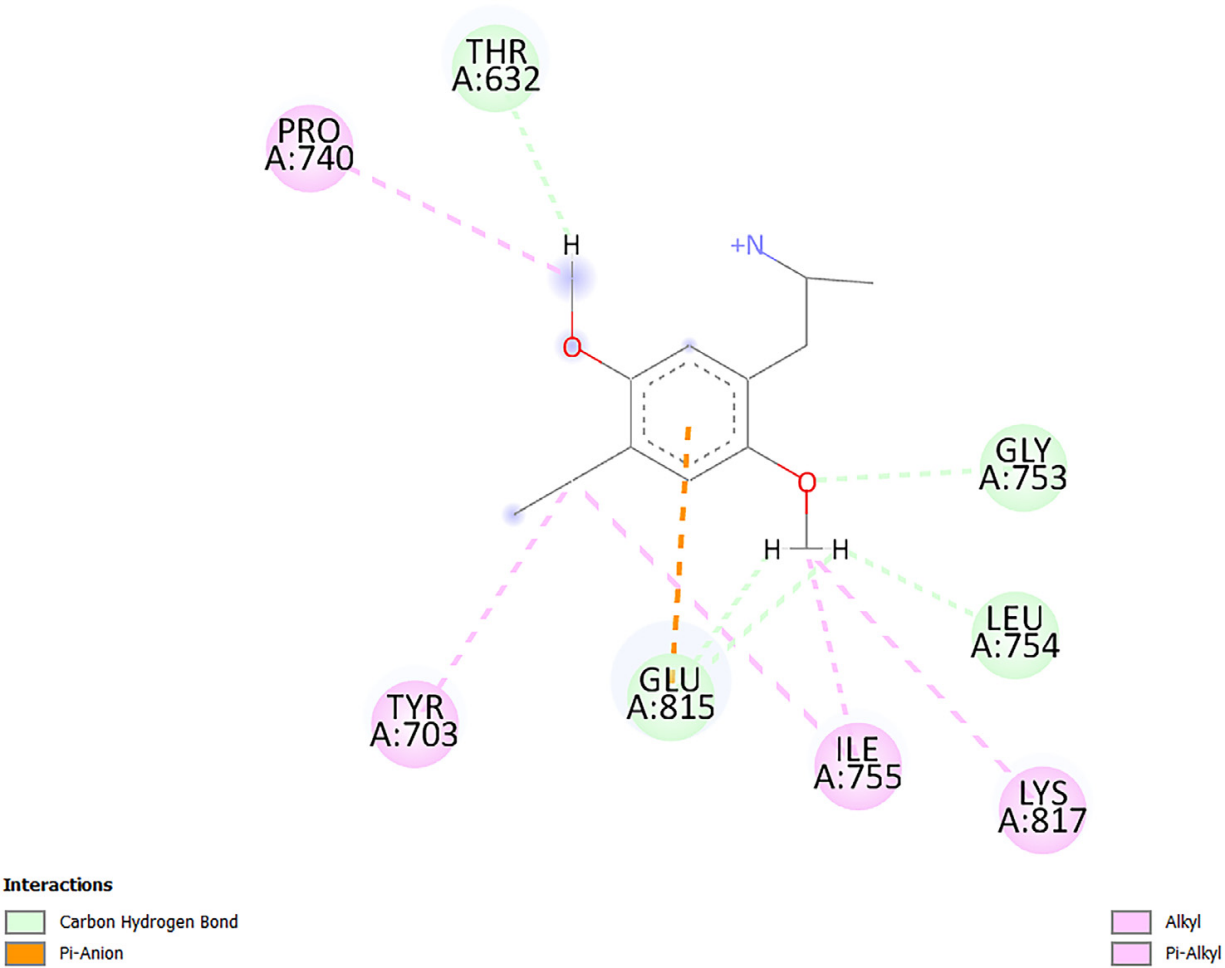
Two-dimensional interaction diagram of 2,5-Dimethoxy-4-ethylamphetamine with human maltase gluco‑amylase (PDB ID: 2QMJ).

**Fig. 46:**
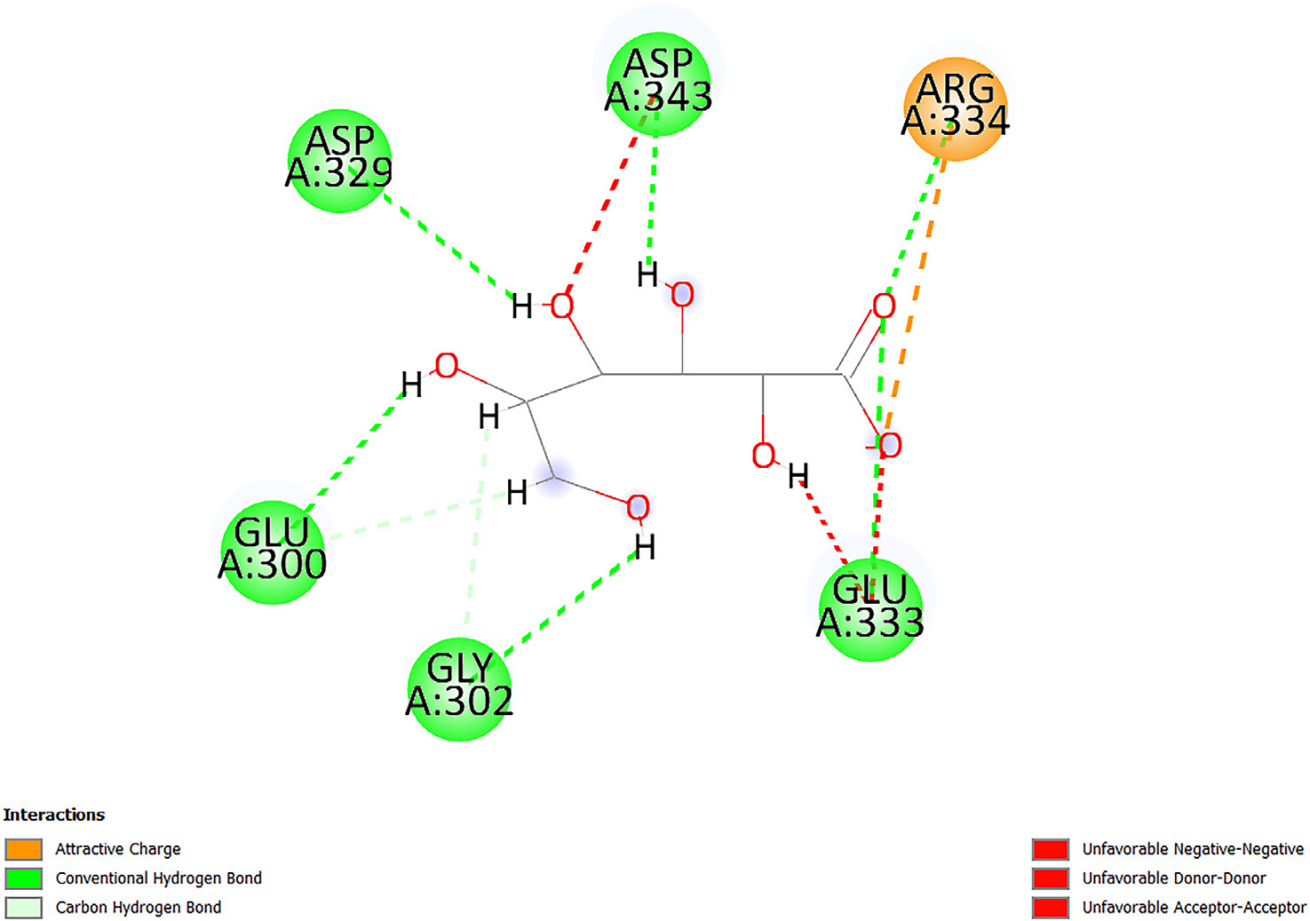
Two-dimensional interaction diagram of Gulonic acid with human maltase gluco‑amylase (PDB ID: 2QMJ), focusing on the interaction dynamics and their implications for enzyme-ligand affinity.

**Fig. 47:**
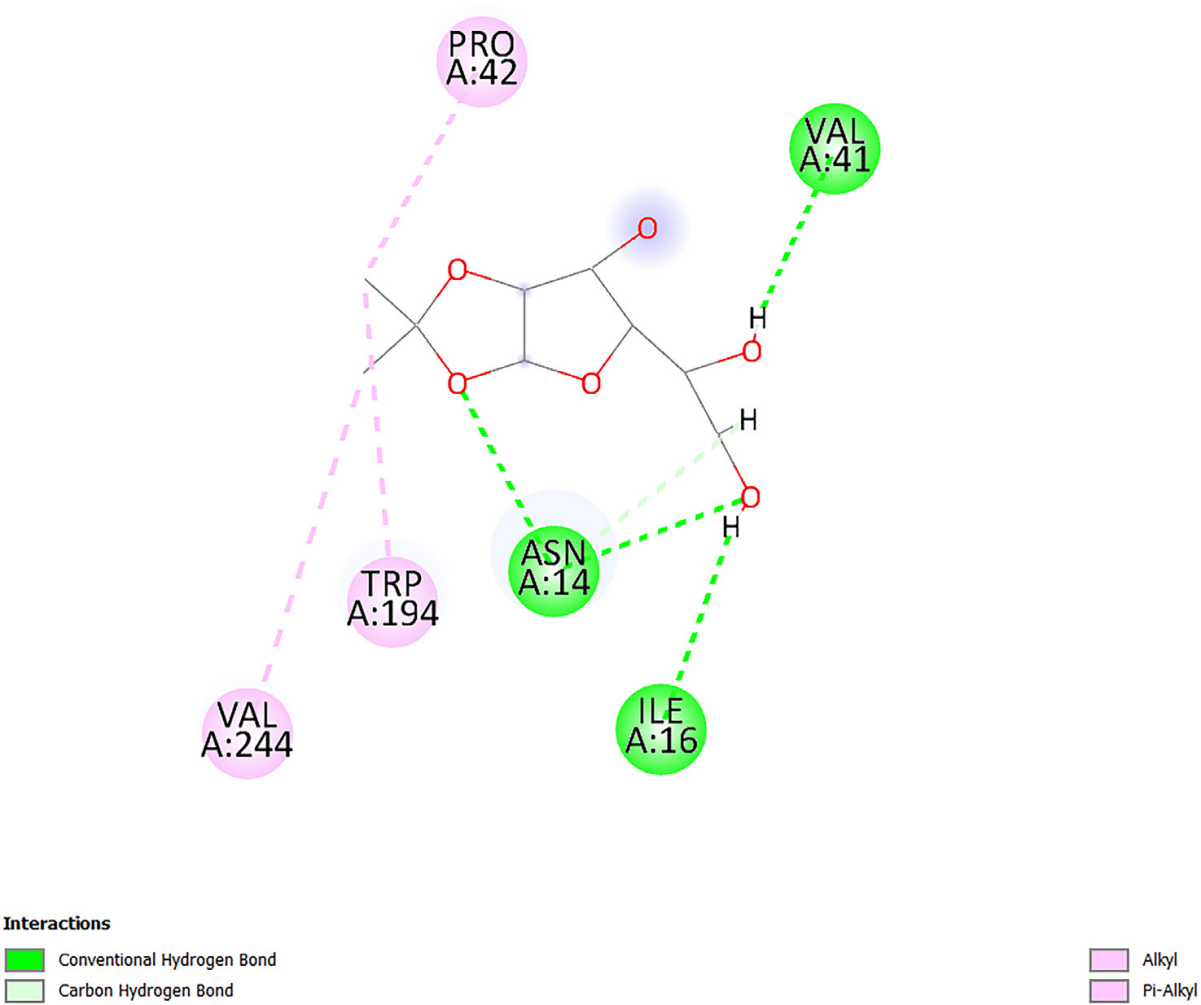
Two-dimensional interaction diagram of 1,2 O-Isopropylidene-alpha-D-glucofuranose with human maltase gluco‑amylase (PDB ID: 2QMJ).

**Fig. 48:**
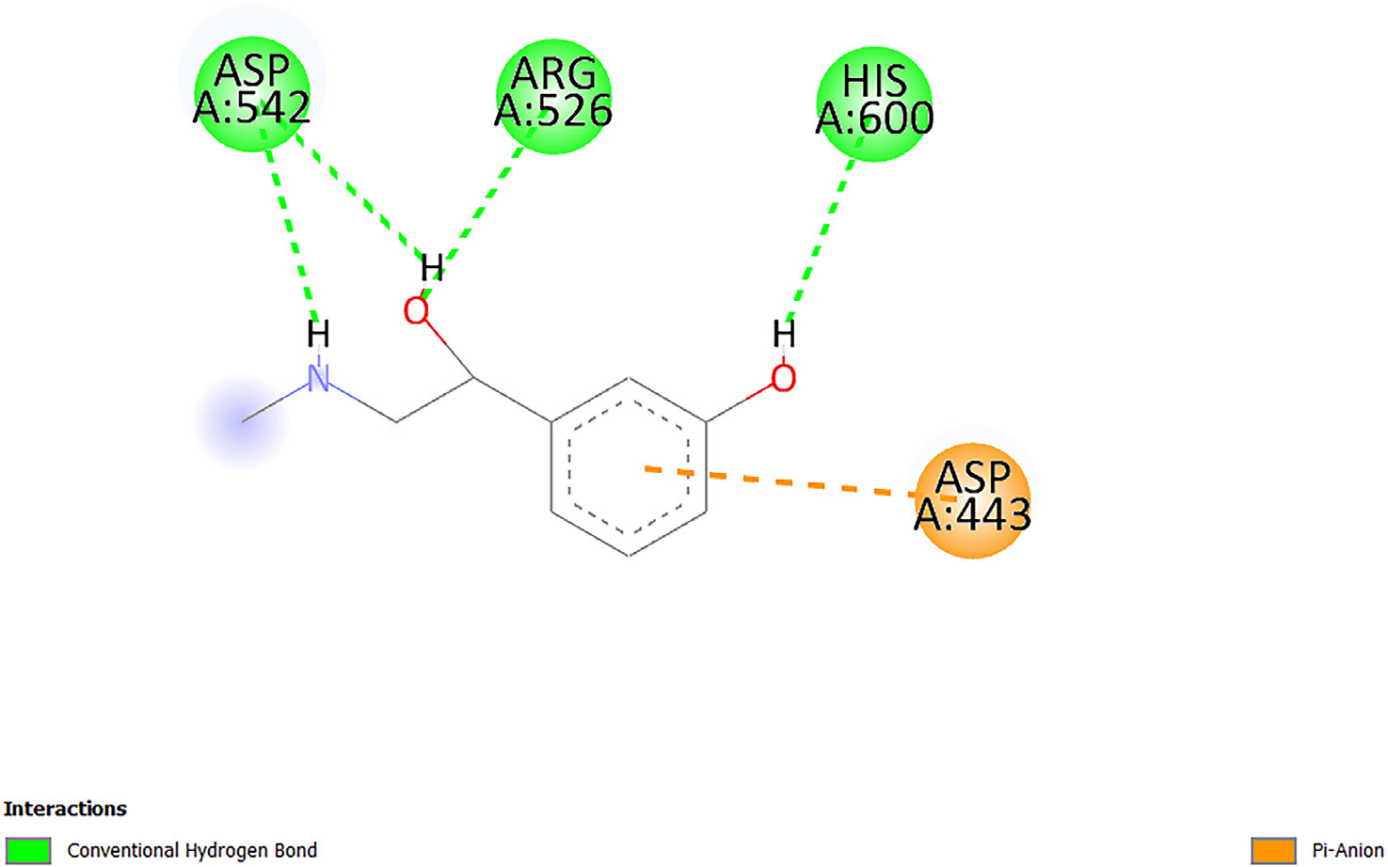
Two-dimensional interaction diagram of Phenylphrine with human maltase gluco‑amylase (PDB ID: 2QMJ).

**Fig. 49:**
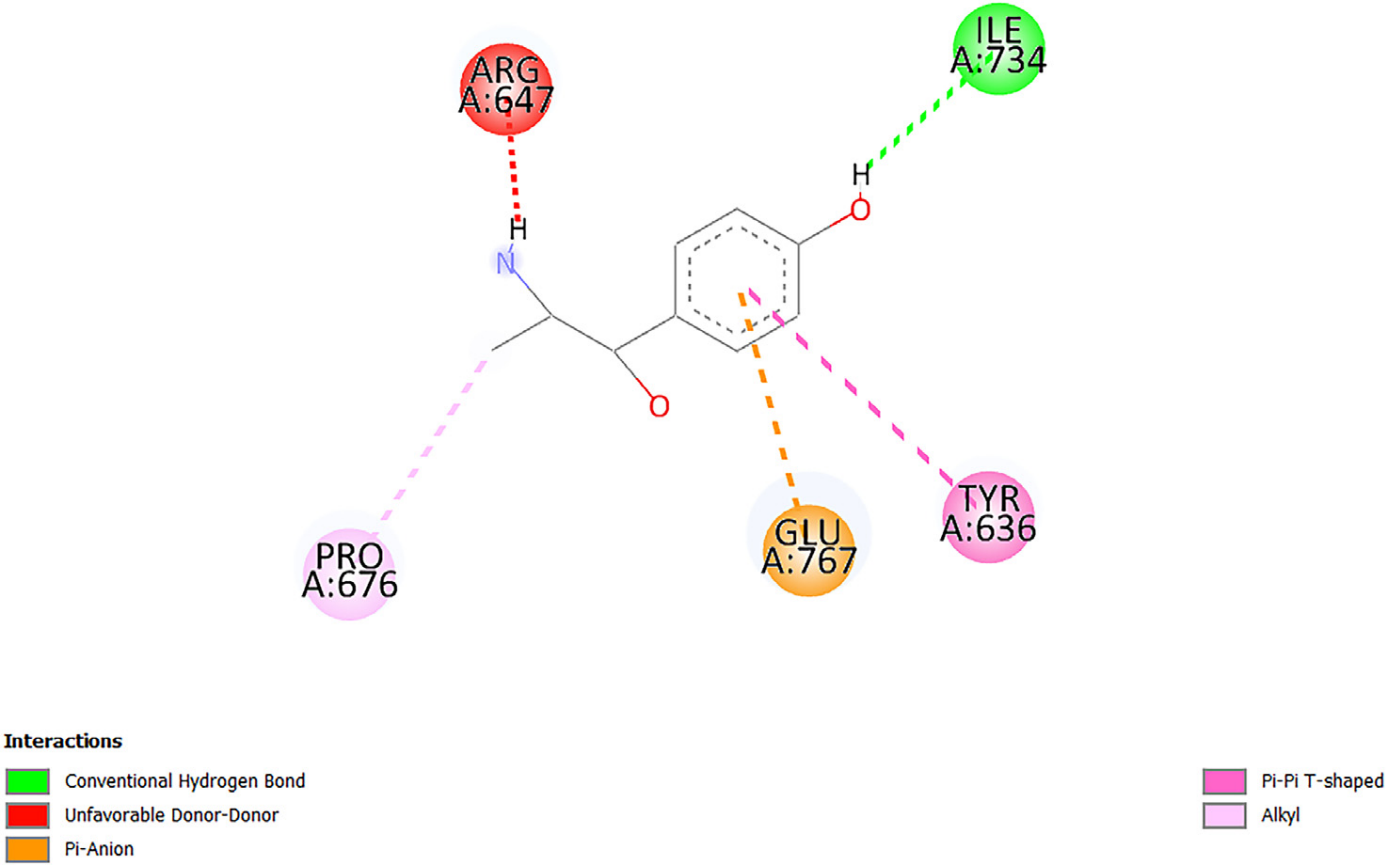
Two-dimensional interaction diagram of p-Hydroxynorephedrine with human maltase gluco‑amylase (PDB ID: 2QMJ).

**Fig. 50:**
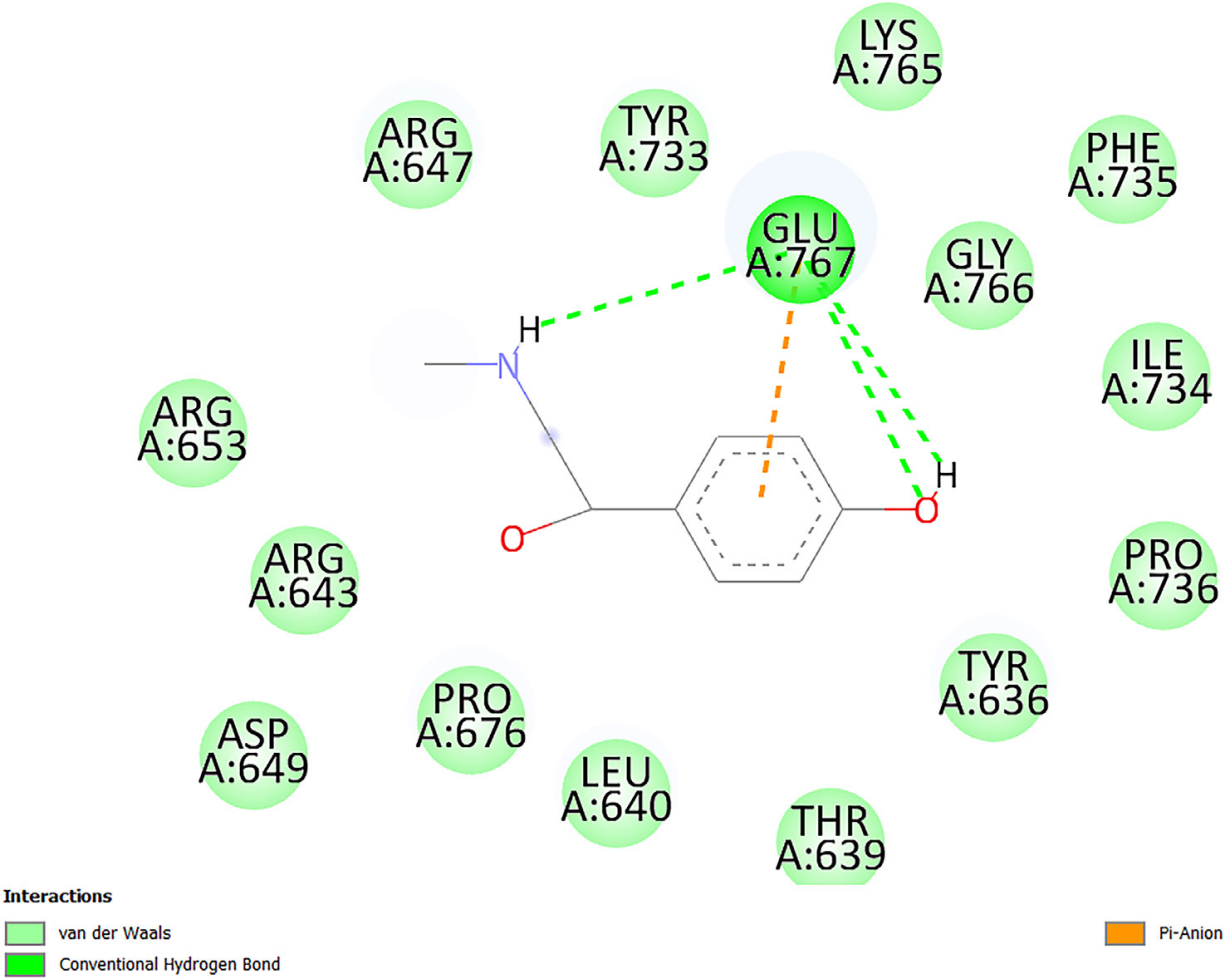
Two-dimensional interaction diagram of Synephrine with human maltase gluco‑amylase (PDB ID: 2QMJ).

**Fig. 51:**
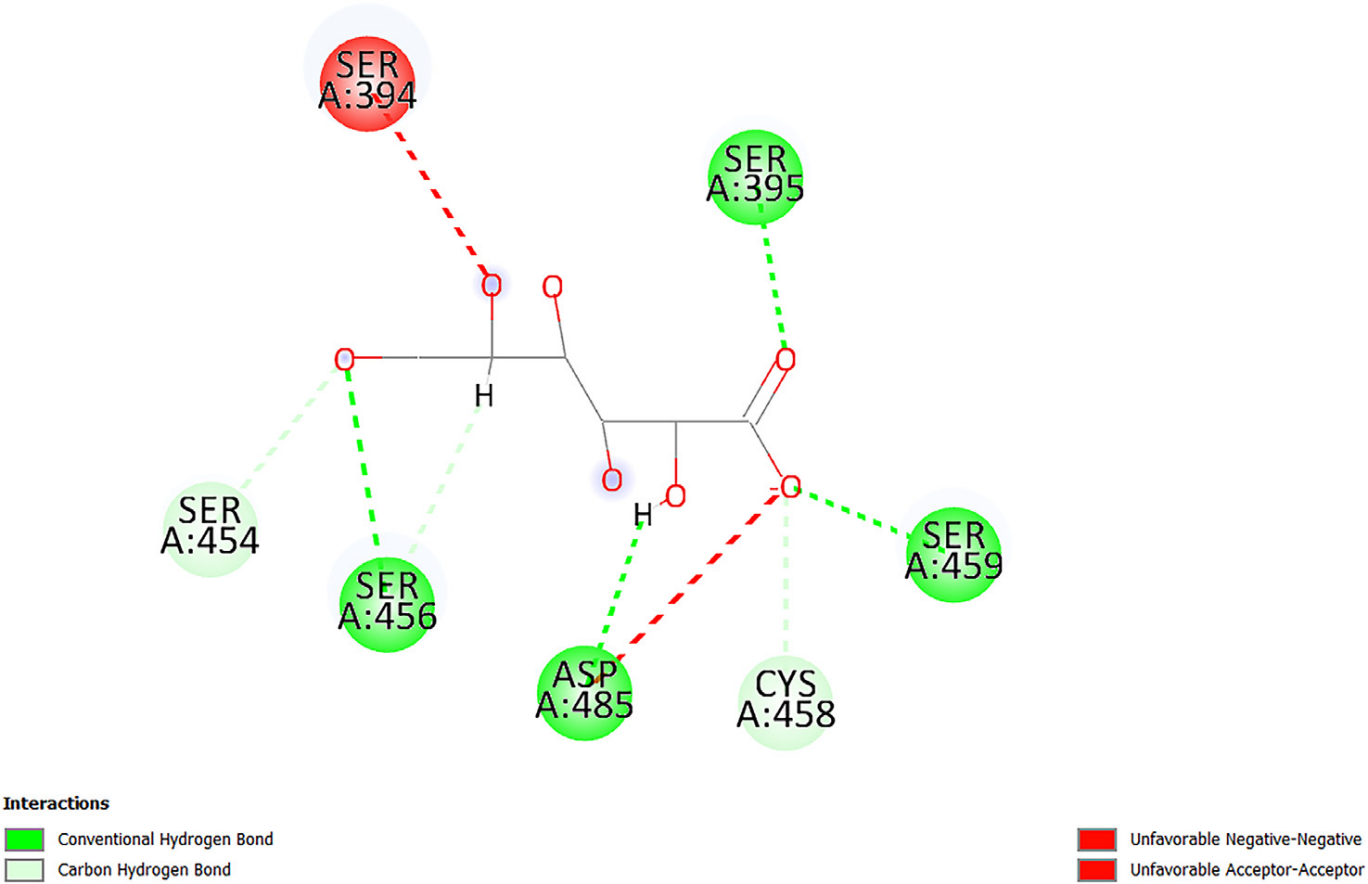
Two-dimensional interaction diagram of Mannoic acid with human maltase gluco‑amylase (PDB ID: 2QMJ).

**Fig. 52:**
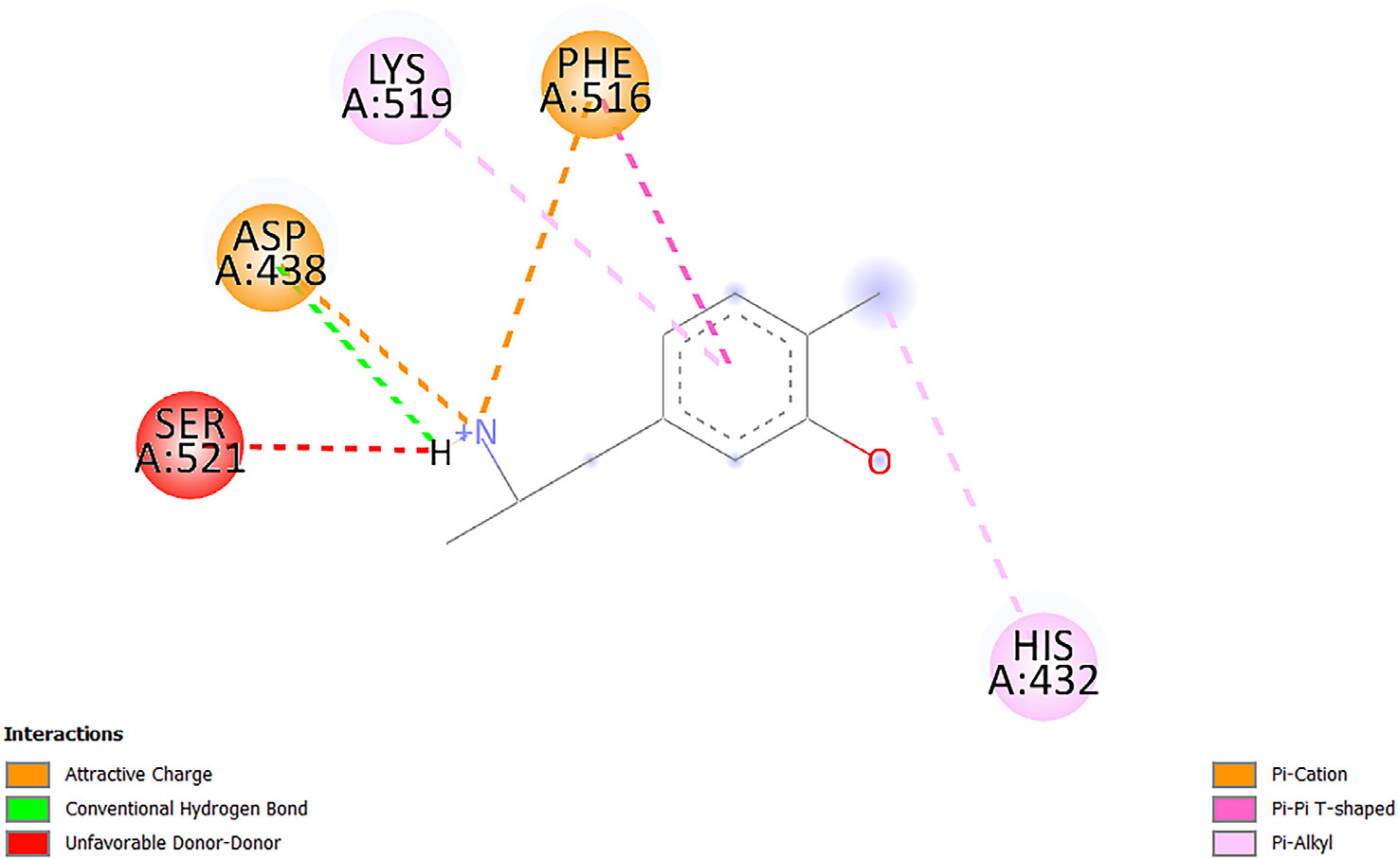
Two-dimensional interaction diagram of 5-(2-Aminopropyl)−2-methylphenol with human maltase gluco‑amylase (PDB ID: 2QMJ).

**Fig. 53:**
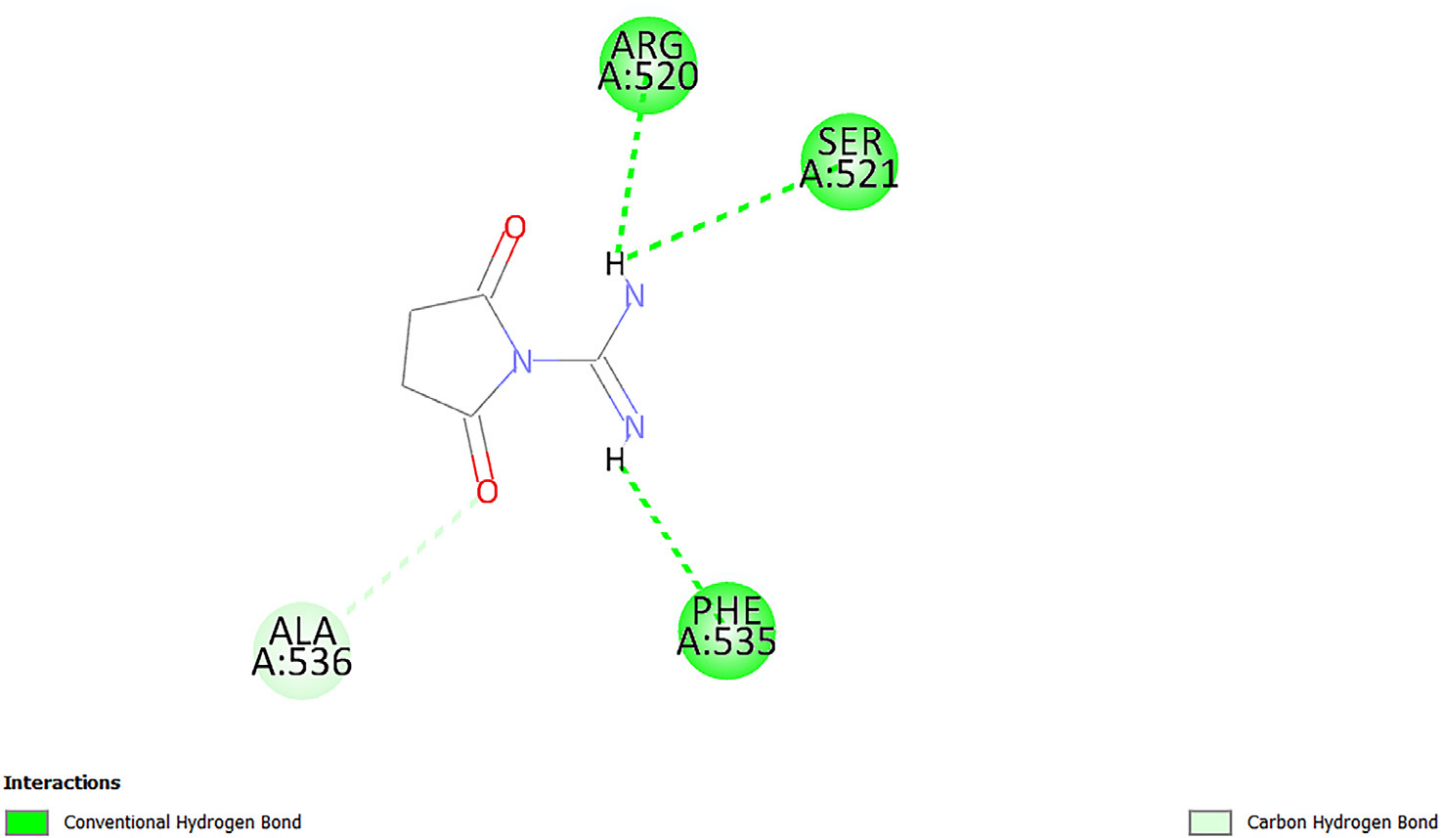
Two-dimensional interaction diagram of Guanidinosuccinimide with human maltase gluco‑amylase (PDB ID: 2QMJ).

**Fig. 54:**
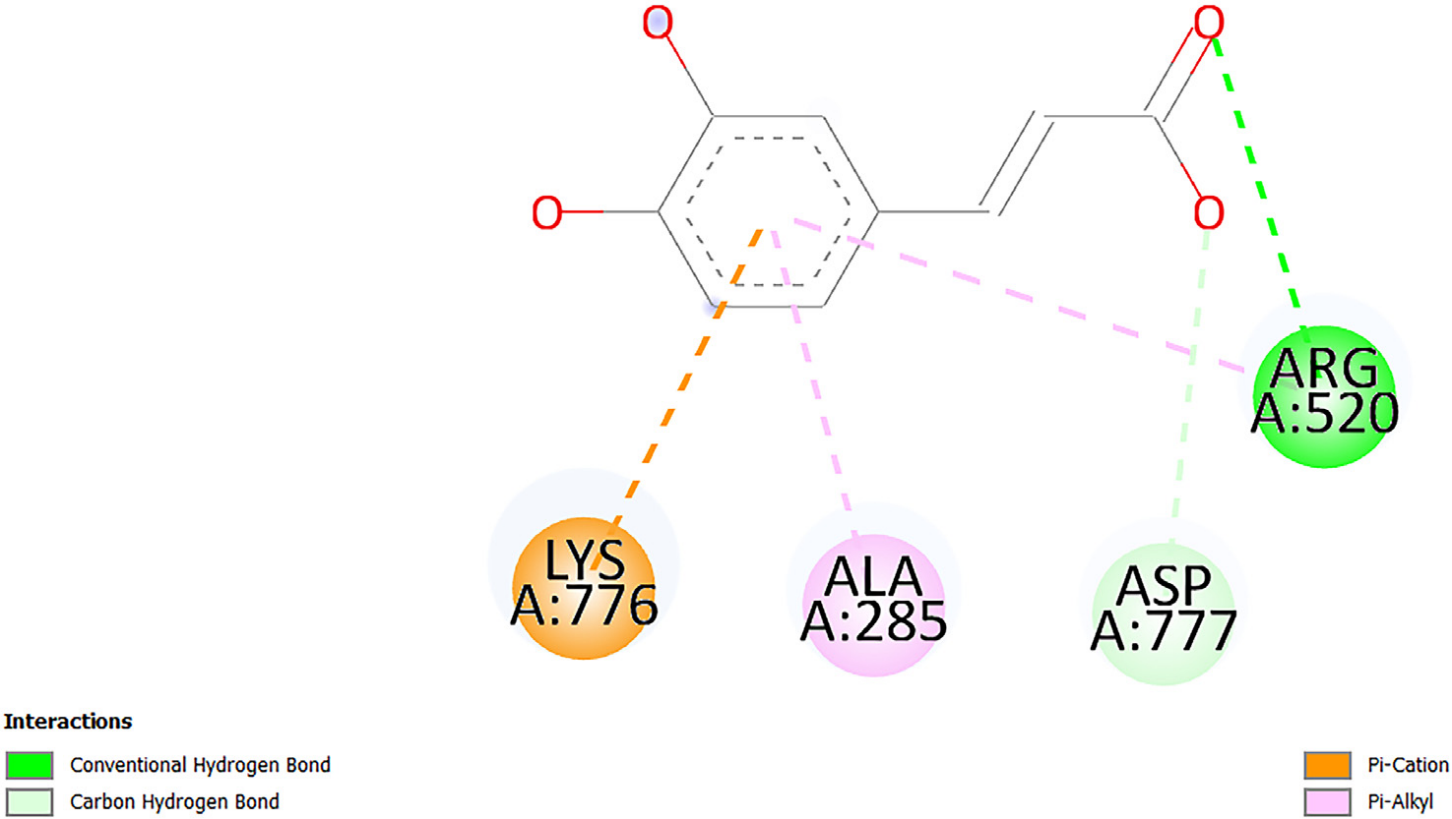
Two-dimensional interaction diagram of Cathine with human maltase gluco‑amylase (PDB ID: 2QMJ).

**Fig. 55:**
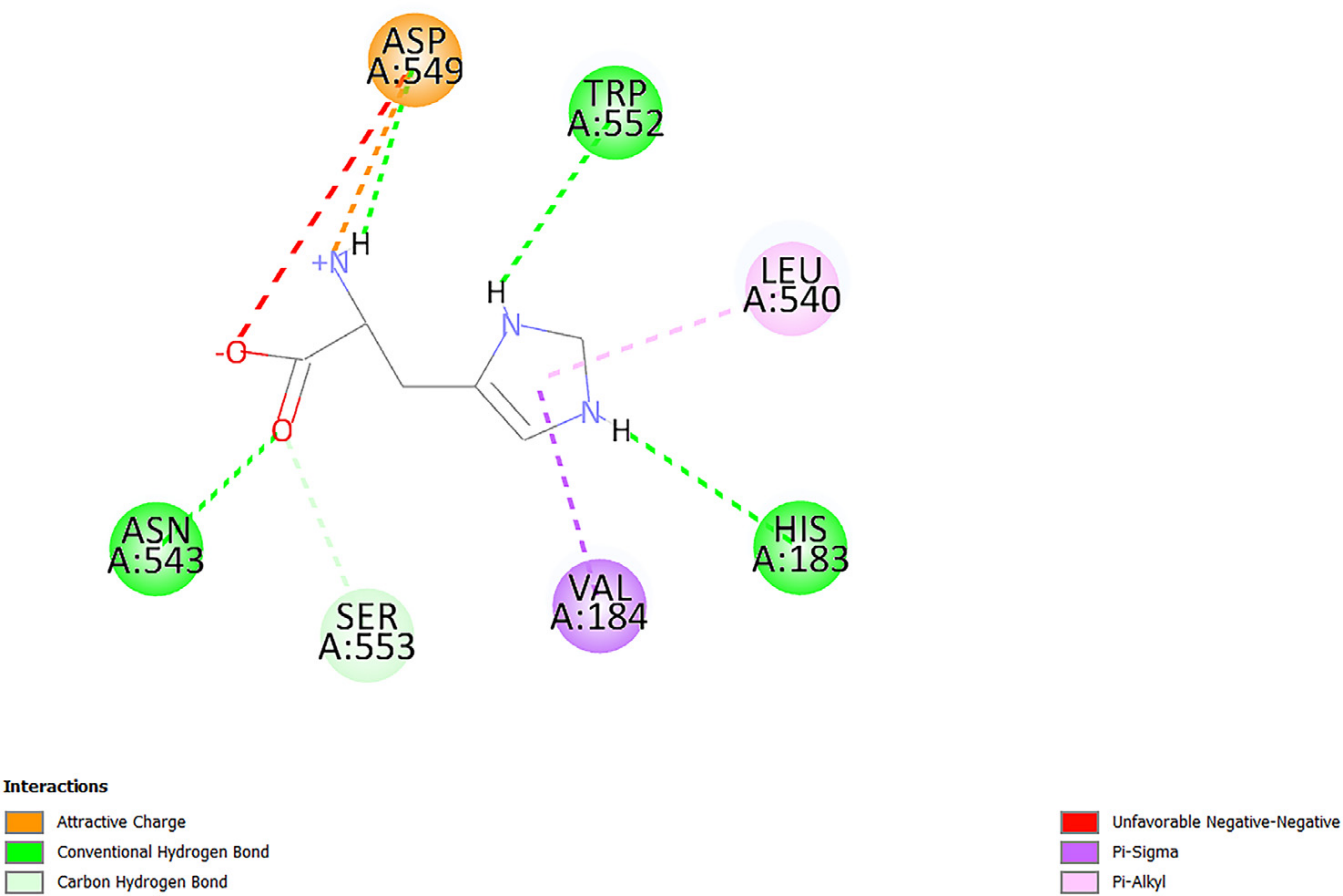
Two-dimensional interaction diagram of l-histidine with human maltase gluco‑amylase (PDB ID: 2QMJ).

**Fig. 56:**
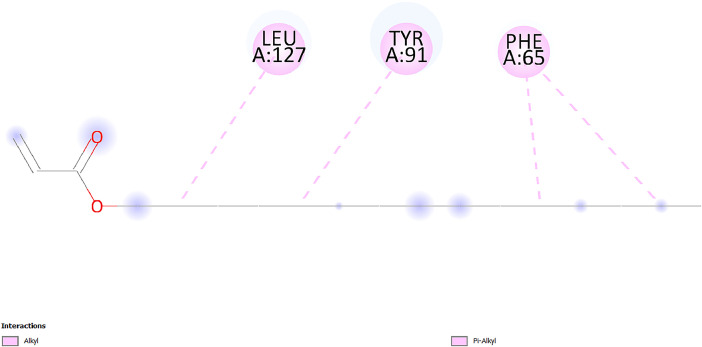
Two-dimensional interaction diagram of 2-Propenoic acid, n-pentadecyl ester with human maltase gluco‑amylase (PDB ID: 2QMJ).

**Fig. 57:**
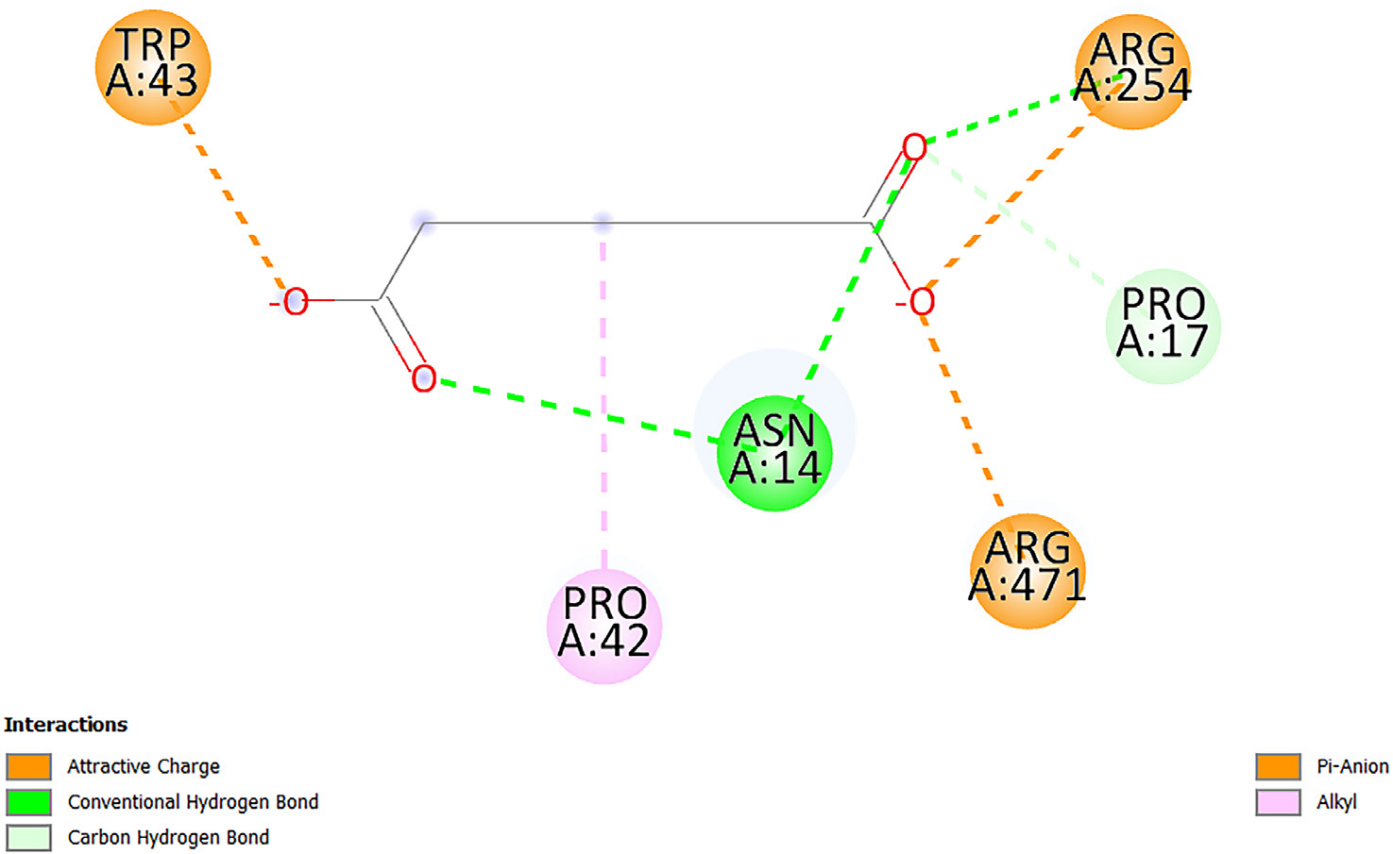
2D interactions of Heptanedioic acid with human maltase gluco‑amylase (PDB ID: 2QMJ).

**Fig. 58:**
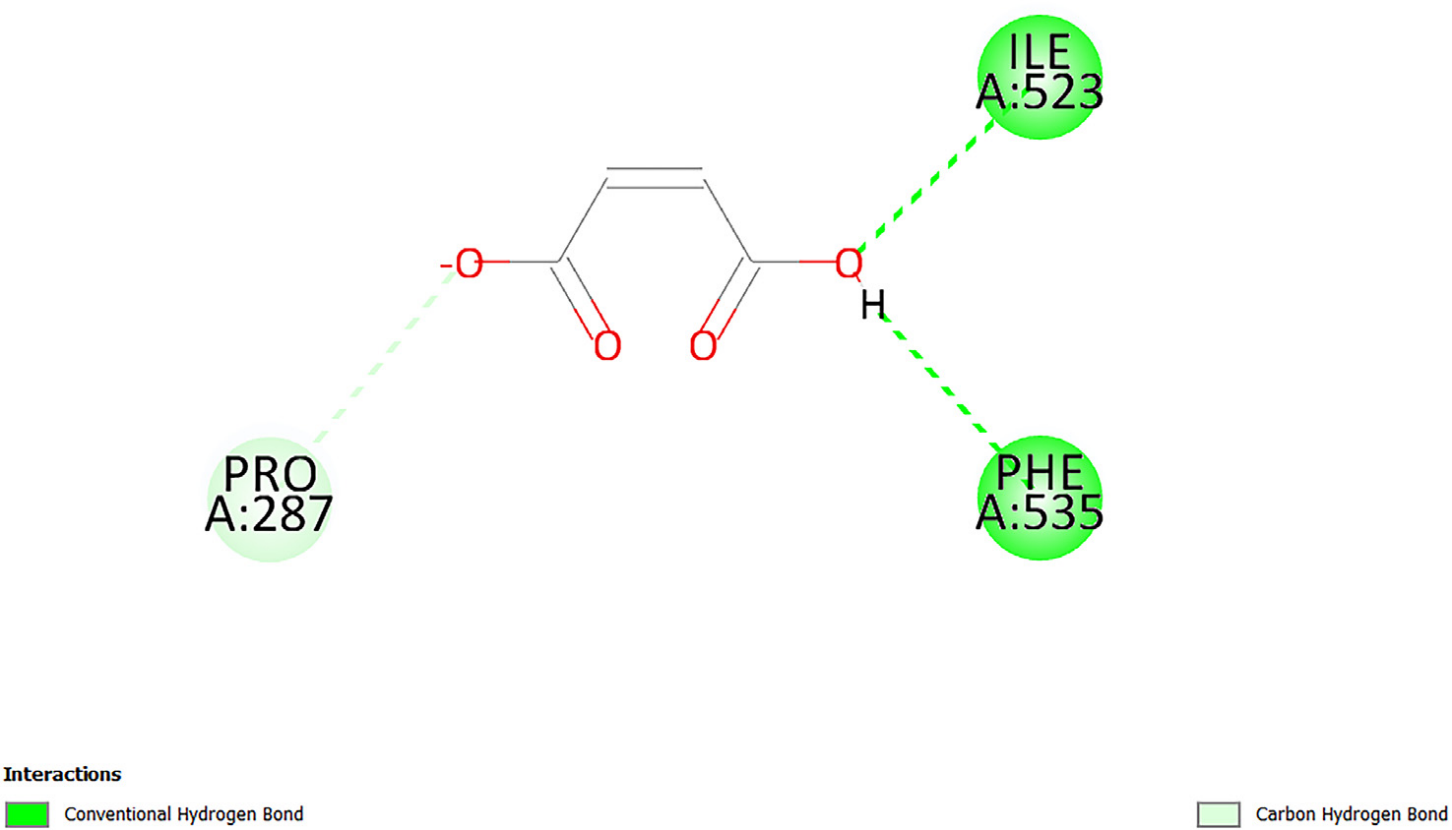
Two-dimensional interaction diagram of 2-Butenedioic acid with human maltase gluco‑amylase (PDB ID: 2QMJ).

**Fig. 59:**
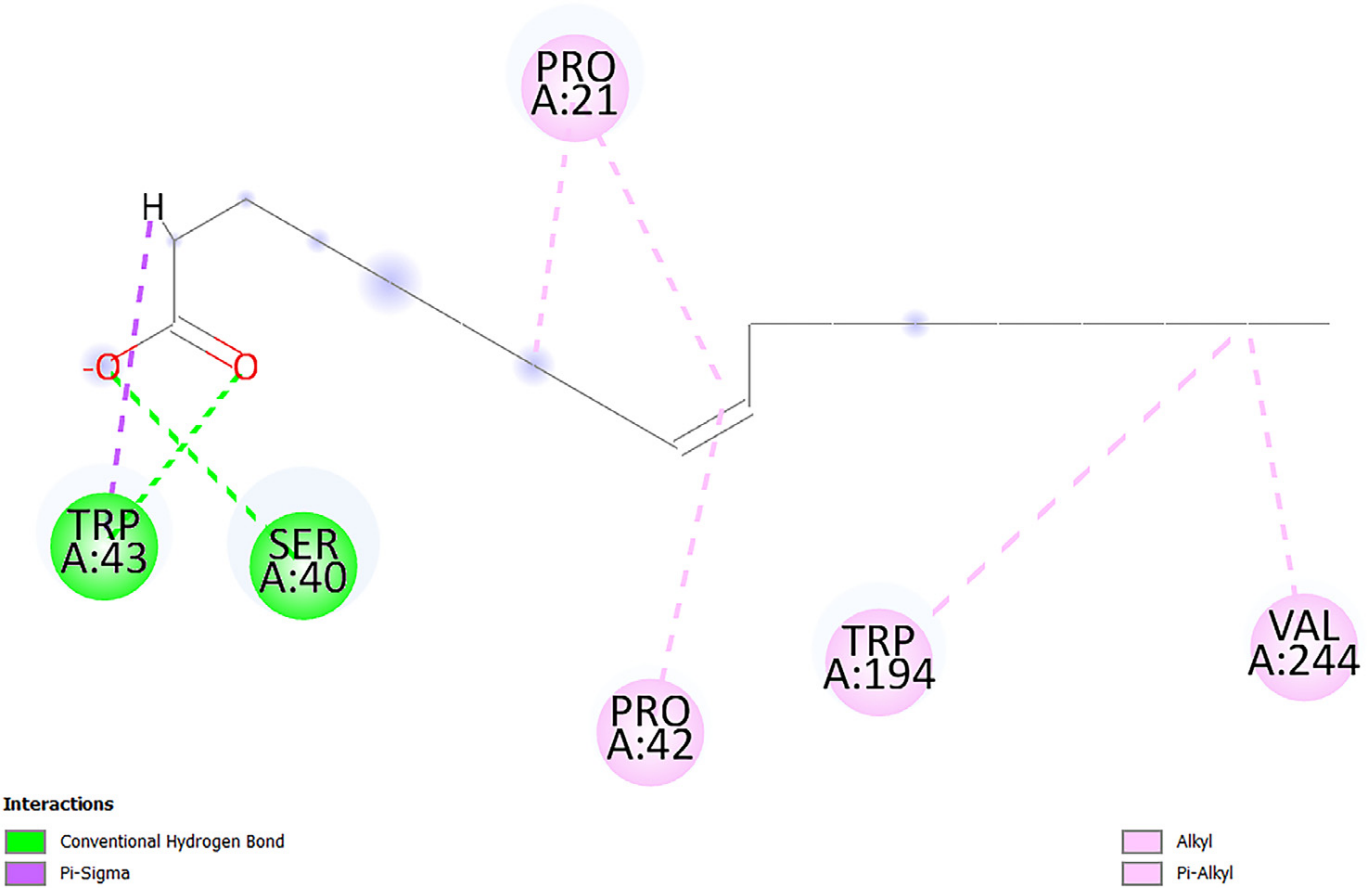
Two-dimensional interaction diagram of 10-Octadecenoic acid with human maltase gluco‑amylase (PDB ID: 2QMJ).

**Fig. 60:**
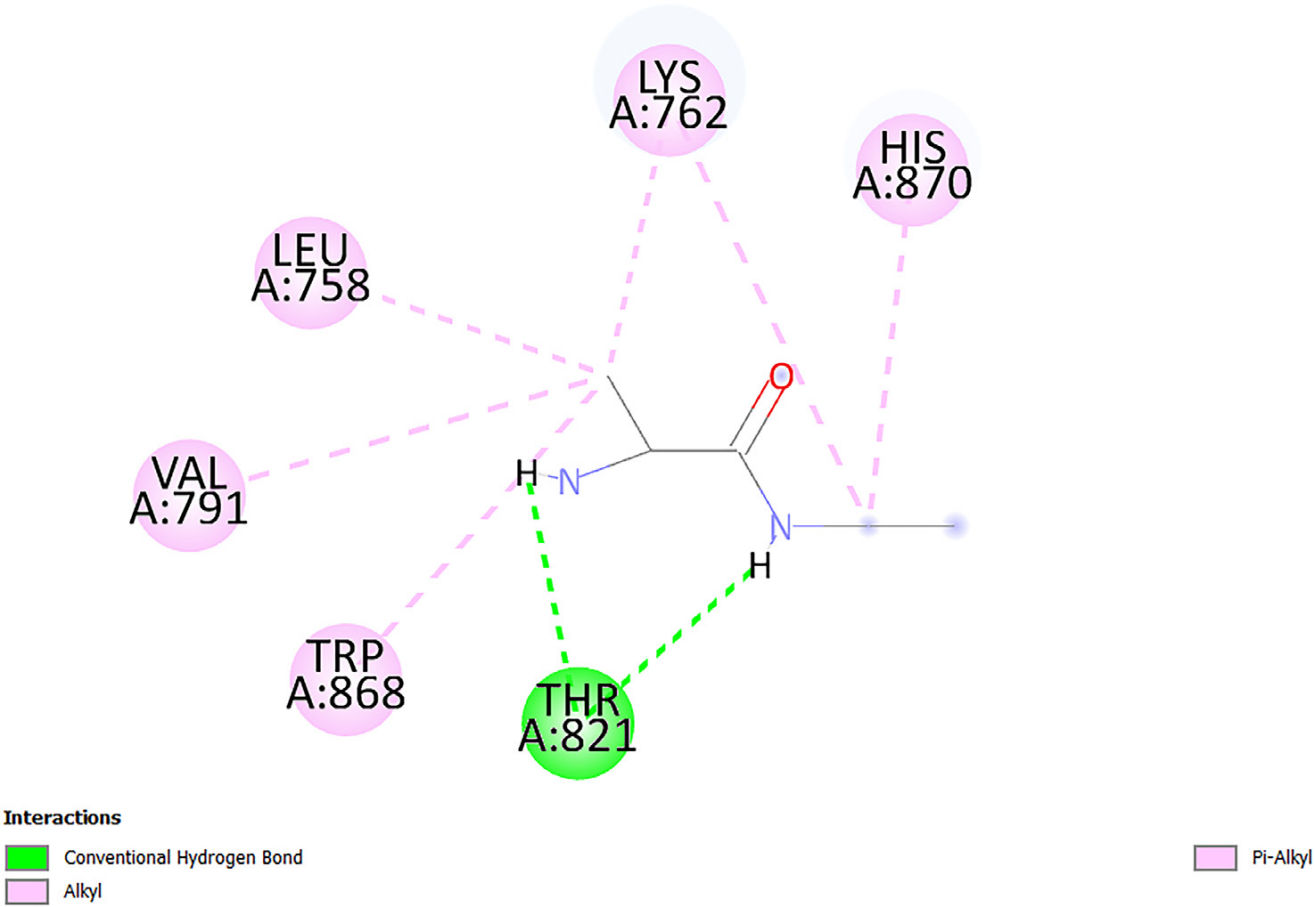
Two-dimensional interaction diagram of L- Alanine ethylamide, (S) with human maltase gluco‑amylase (PDB ID: 2QMJ).

## 4 Experimental Design, Materials and Methods

### 4.1 Protein preparation

In this molecular docking study, which aims to control metabolic disorders, two target proteins were selected: Human maltase-glucoamylase in complex with acarbose (PDB ID: 2QMJ) and human pancreatic alpha-amylase in complex with nitrite (PDB ID: 2QMK). The 3D structures of these proteins were obtained from the RCSB Protein Data Bank (https://www.rcsb.org/) [15]. The details are summarised in Table 1. The preparation was performed using YASARA software (version 19.12.14). The preparation process included the following steps: (i) calculation of Gasteiger charges, (ii) addition of hydrogen atoms and Kolman charges, (iii) removal of water molecules and heteroatom coordinates, and (iv) restoration of missing C-terminal oxygen atoms [1]. To ensure the structural integrity of the protein, preprocessing involved a series of careful steps: calibration of binding parameters, definition of bonds and bond orders, correction of incorrectly assigned elements, reconstruction of missing side chain loops, removal of superfluous chains, capping of uncapped C- and N-termini and formal charges for metal ions for optimal stabilisation [16]. Priority was given to hydrogen bonding in the optimisation strategy, while other factors were reduced by using AMBER14 for the field after hetero-group removal. The quality of the prepared proteins was assessed using the SiteMap application and the files were saved in pdbqt format for further analysis.

### 4.2 Ligands preparation

A total of 25 ligands were selected for this study, the 3D structures of which were taken from the PubChem database (https://pubchem.ncbi.nlm.nih.gov/). Prior to screening, ligand preparation was performed using Maestro software [17], which included several important steps: the creation of 3D geometries, valence recognition and the identification of accessible tautomers. Furthermore, several parameters carefully include minimization of energy, hydrogen atoms addition and torsion angles calculation. The ligand structures were energetically minimised using a gradient of 0.05, assigning the correct bond orders and charges [18]. The geometry was optimized using the steepest gradient method over a total of 100 iterations.

### 4.3 Molecular docking

Molecular docking was performed to identify potential ligands that could serve as inhibitors by targeting the binding sites of the selected proteins to treat metabolic disorders. The identification of binding sites was performed using the online web tool YASARA [19]. In contrast to conventional scoring approaches, this study focused on the interaction between the target receptor proteins and the ligands as determined by the positive binding energy scores. The Clean module was used to process the crystallised ligand coordinates and thus ensure optimal preparation for the subsequent analysis. In addition, Vina generated docking sites that were checked with the AMBER04 force field, which determined the atom types and confirmed their similarity to the original positions. The involvement of specific amino acids and the types of binding that contribute to intermolecular interactions between potent ligands and target proteins were visualised with the AMBER4 force field using ACCELRYS Discovery Studio [20].

## Limitations

No in vitro experiments or animal studies were performed in the present study, which are crucial for validating the molecular docking results and confirming the biological activity of the identified interactions.

## Ethics Statement

The authors have read and follow the ethical requirements for publication in Data in Brief and confirming that the current work does not involve human subjects, animal experiments, or any data collected from social media platforms.

## CRediT Author Statement

The authors declare that they have no known competing financial interests or personal relationships which have or could be perceived to have influenced the work reported in this article.

## Data Availability

Mendeley Datadataset for millet derived compound fight against diabietic protein alpha-amylase and human maltase gluco‑amylase (Original data).

## Appendix Supplementary materials

Image, application 1
