# Supplementary material for: Data on the docking of millet-derived secondary metabolites as multi-target ligands for diabetes
Source: Data Brief. 2025 Jan 11;59:111290. doi: 10.1016/j.dib.2025.111290 (PMC11808624; doi:10.1016/j.dib.2025.111290)
Supplement: Supplementary file 1 [file mmc1.docx]

**Suppl. Table 1: Vander wells interaction of 2QMK with active site of target compound**

| **Bioactive compound** | **Interaction by amino acid residues** |
| --- | --- |
| **Actinobolin** | Gly334, Ser289, His331, Val401, Thr11, Gln404, Ser3, Pro4, Phe335, Asp290 |
| **Xanthosine** | Arg252, Gly403, Ser289, Tyr333, Asp402, Arg421, Gly9, Thr6, Ser3 |
| **Arbutin** | Thr6, Arg10, Thr11, Arg421, Arg398, Pro332, Tyr333, Ser289, Asp290, Arg252, Asp290, Phe335, Ser3 |
| **Sinapic acid** | Gln7, Gly9, Thr11, Arg421, Gly403, Pro332, Tyr333, Gly334, Asp290, Pro4, Phe335 |
| **Maltose** | Asn5, Ser3, Tyr2, Thr336, Arg421, Tyr333, Phe335, Thr11, Asp290, Arg252, Asp402 |
| **L-Tryptophan** | Asp197, Ala198, His101, Leu162, Leu165, Trp59, His305, Trp58, Ile235, His299, Phe256 |
| **Caffeic acid** | Val401, Arg398, Gly334, Ser289, Phe335, Ser3, Pro4, Gln8, Thr11, Gly403 |
| **Pyrido[3,4-d] imidazole, 1,6-dicarboxylic acid** | Tyr333, Ser289, Asp290, Phe235, Pro4, Thr336, Val401, Arg421, Gly403, Tyr333, Ser289 |
| **Prosta 5,13-dien-1-oic acid** | Trp59, His 201, Tyr151, Lys200, Glu233, Arg195, Phe256, Asn298, His299, Asp300, His305, Trp58, Thr163 |
| **Tyrosine** | His101, Leu165, Gln63, Trp59, His305, Trp58, Ile235, Phe256, Asp197, His299, Ala198, Leu162 |
| **4-Fluoro-3-[1-hydroxy-2-(methylamino)ethyl] phenol** | Thr6, Gln7, Arg10, Thr336, Asp402, Gly403, Arg421, Val401, Arg252 |
| **D-Fructopyranose** | Thr336, Gly334, Gln404, Asp402, Val401, Gly403 |
| [**3-Ethoxy-4-hydroxymandelic acid**](https://pubchem.ncbi.nlm.nih.gov/compound/193792) | Arg421, Val401, Gly403, Gln404, Pro332, Gly334, Arg252, Phe335, Pro4, Thr6, Gln7, Arg10 |
| **Gluconic acid** | Thr336, Val401, Gly403, Thr11, Gln8, Phe335 |
| [**2,5-Dimethoxy-4-ethylamphetamine**](https://pubchem.ncbi.nlm.nih.gov/compound/27402) | Gly403, Pro332, Gly334, Arg252, Asp290, Ser3, Tyr2, Gln8, Thr6, Gly9, Gln7, Thr11, Arg398, Val401 |
| **Gulonic acid** | Val401, Thr11, Gly403, Ser289, Tyr333, Phe335, Arg398 |
| **1,2 O-Isopropylidene-alpha-D- glucofuranose** | His331, Arg421, Val401, Gln404, Asp402, Phe335, Arg252, Asp290, Tyr333, Thr336 |
| **L-**[**phenylephrine**](https://pubchem.ncbi.nlm.nih.gov/compound/6041) | Gln7, Ser3, Asn5, Pro4, Arg252, Pro332, Gly9 |
| [**p-hydroxynorephedrine**](https://pubchem.ncbi.nlm.nih.gov/compound/11099) | Asp402, Arg421, Gly403, Thr336, Tyr2, Ser3, Arg252 |
| [**Synephrine**](https://pubchem.ncbi.nlm.nih.gov/compound/7172) | Ser3, Asn5, Gly9, Thr11, Arg398, Gly334, Arg252, Ser289, Pro4 |
| **Mannoic acid** | Gln404, Gly403, Arg421, Asp402, Thr11, His331, Thr336 |
| **5-(2-Aminopropyl)-2-methylphenol** | Ile235, Asn298, His299, Phe256, Arg195, Asp197, Ala198, His101, Leu162, Gln63, Trp59, His305, Trp58 |
| [**I-Guanidinosuccinimide**](https://pubchem.ncbi.nlm.nih.gov/compound/541546) | Phe348, Gly304, Arg346, Gln302, Thr314, Ser311, Ala310, Arg267, Trp269 |
| **Cathine** | Lys322, Thr377 |
| **L-Histidine** | Trp59, His305, Ile235, Phe256, His299,Trp58 |
| **2-Propenoic acid,**  **n-pentadecyl ester** | Ala318, Asp375, Thr377, Cys378 |
| **Heptanedioic acid** | Val401, Pro332, Gly334, Pro4, Thr6, Gly9, Arg10, Phe335, Thr11, Gly403 |
| **2- Butendioic acid** | Phe335, Gly334, Val401, Gly403, Thr11 |
| **10-Octadecenoic acid** | Tyr151, Glu233, Val234, Arg195, His101, Asp197, Asp300, Trp58, Thr163 |
| **L- Alanine ethylamide (S)** | Trp269, Gly304, Arg303, Gln302, Thr314, Asp317 |
| **11-Eicosenoic acid** | Gln7, Thr6, Arg10, Thr11, Pro332, Arg398, Gln404, Val401, Gly334, Arg252, Ser289, Asp290, Gln8 |

**Suppl. Table 2: Vander wells interaction of 2QMJ with active site of target compound**

| **Bioactive compound** | **Interaction by amino acid residues** |
| --- | --- |
| **Actinobolin** | Arg254, Gln246, Asn14, Pro42, Val 41, Trp 43, Cys44, Pro21, Ile16, Pro20, Pro17, Gln19 |
| **Xanthosine** | Arg712, Gly731, Glu763, Ala764, Tyr733, Arg730, Ala768 |
| **Arbutin** | Tyr733, Gly766, Lys765, Phe789, Ile734, Phe735, Thr639, Leu640, Pro676, Arg643, Glu271 |
| **Sinapic acid** | Pro4, Gln7, Gly9, Thr11, Arg421, Gly403, Pro332, Tyr333, Gly334, Asp290, Phe335, Pro4 |
| **Caffeic acid** | Lys534, Phe535, Ala509, Ile523, Leu286, His645, Thr778, Thr775 |
| **Tyrosine** | Phe789, Pro736, Gly766, Phe735, Lys765, Tyr733, Gly765, Arg643, Leu640, Thr639 |
| **Maltose** | Phe735, Pro736, Tyr636, Pro676, Glu271, Arg643, Leu640, Thr639 |
| **4-Fluoro-3-[1-hydroxy-2-(methylamino)ethyl] phenol** | Gly533, Ala536, Lys534, Ala509, Ile523, Phe522, Ser288, Ala291 |
| [**p-hydroxynorephedrine**](https://pubchem.ncbi.nlm.nih.gov/compound/11099) | Arg653, Asp649, Gly765, Arg643, Leu640, Thr639, Pro736, Phe735, Phe789, Gly766, Tyr733, Lys765 |
| **Cathine** | Lys534, Phe535, Ala509, Ile523, Asp777, His645, Thr778, Thr775 |
| **L-Histidine** | Pro570, Pro206, Trp587, Glu182, Phe560, Gly556, Glu559, Pro555, His185, Gln186 |
| **Gluconic acid** | Lys100, Glu90, Ser118, Ile259, His113, Met236, Val111, Glu110, Tyr263 |
| **L-Tryptophan** | Pro736, Tyr636, Thr639, Leu640, Arg643, Asp649, Tyr733, Glu271, Lys765, Gly766, Ile734 |
| **L-**[**Phenylephrine**](https://pubchem.ncbi.nlm.nih.gov/compound/6041) | Thr205, Tyr299, Asp327, Ile328, Ile364, Trp441, Trp406, Trp539, Phe575, Asp203 |
| [**Synephrine**](https://pubchem.ncbi.nlm.nih.gov/compound/7172) | Arg653, Arg643, Asp649, Pro676, Leu640, Thr639, Tyr636, Pro736, Ile734, Gly766, Phe735, Lys765, Tyr733, Arg647 |
| **Pyrido[3,4-d] imidazole, 1,6-dicarboxylic acid** | Ser456, Val455, Ile402, Ser454, Cys483, Ser394 |
| [**2,5-Dimethoxy-4-ethylamphetamine**](https://pubchem.ncbi.nlm.nih.gov/compound/27402) | Thr737, Gln738, Leu752, Gln739, Asn814, Pro751, Ile816 |
| **1,2 O-Isopropylidene-alpha-D- glucofuranose** | Ser40, Thr196, Arg471, Gln19, Pro21, Cys44, Trp43 |
| **Prosta 5,13-dien-1-oic acid** | Thr204, Asp203, Phe450, Met444, Trp539, Asp443, Asp542, Gln603, Tyr605, Gly602, Thr205, Tyr214 |
| [**3-Ethoxy-4-hydroxymandelic acid**](https://pubchem.ncbi.nlm.nih.gov/compound/193792) | Lys817, Leu754, Asn814, Leu752, Pro751, Tyr703, Ile629, Thr632, Ile755 |
| [**I-Guanidinosuccinimide**](https://pubchem.ncbi.nlm.nih.gov/compound/541546) | Met567, Ala537, Gly533, Ala285, Ile523, Leu286, Ser288, Phe255, Ala291, Phe522, Pro287 |
| **D-Fructopyranose** | Pro751, Gln739, Gly753, Thr737, Pro740, Thr632, Ile755 |
| **Gulonic acid** | Thr303, Asp332, As340, Met331, Phe344 |
| **5-(2-Aminopropyl)-2-methylphenol** | Trp290, Arg520, Ser288, Glu774, Lys360, Glu436, Val515, Pro517 |
| **11-Eicosenoic acid** | Tyr605, Arg334, Asp203, Met444, Arg526, Asp542, Asp327, Asp443 |
| **Mannoic acid** | Val455, Ile402, Leu401, Cys483, Gly457, Thr399 |
| **2- Butendioic acid** | Phe641, Pro566, Pro284, Ala285, Gly533, Ala536, Phe522, Ala537, Met567 |
| **Heptanedioic acid** | Trp194, Ser40, Val41, Pro21, Ile16, Thr196, Val244, Thr196 |
| **L- Alanine ethylamide (S)** | Thr792, Arg795, Asn794, Gln793, Ile818, Glu822, Gly820 |
| **10-Octadecenoic acid** | Val39, Ala38, Gln19, Pro17, Ile16, Arg471, Arg254, Arg471, Asn14, Thr196, Arg254 |
| **2-Propenoic acid,**  **n-pentadecyl ester** | Arg145, Tyr129, Gly64, Glu132, Leu58, Ile133, Asn60, Ala123, Gln130, Val131 |
